# Defining the Speciation, Coordination Chemistry, and Lewis Acid Catalysis of Electronically Diverse Zinc Benzoates

**DOI:** 10.1021/acs.organomet.4c00358

**Published:** 2024-12-16

**Authors:** Lydia A. Dunaway, Audrey G. Davis, Victoria J. Carter, Albert K. Korir, Matthias Zeller, John J. Kiernicki

**Affiliations:** a Department of Chemistry, Drury University, Springfield, Missouri 65802, United States; b H.C. Brown Laboratory, Department of Chemistry, Purdue University, West Lafayette, Indiana 47907, United States

## Abstract

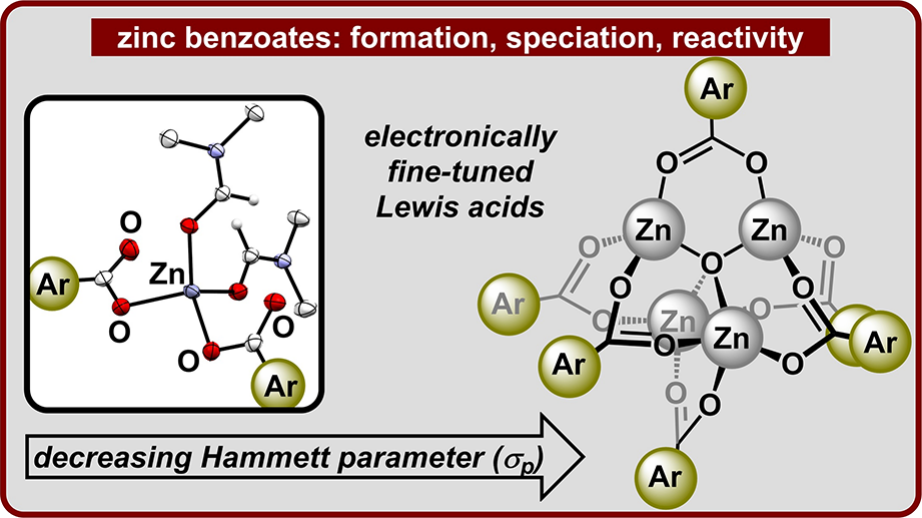

Zinc benzoates may provide an element of tunability that is not available to their ubiquitous acetate analogues. Unfortunately, the synthesis, speciation, and coordination chemistry of zinc benzoates are less developed than the acetates. In this study, we systematically investigate zinc benzoates to understand their propensity to favor solvate (Zn(O_2_CAr)_2_(L)_2_) or cluster (Zn_4_O(O_2_CAr)_6_) formation as well as their utility as metal complex precursors. The zinc benzoates were found to be Lewis acid catalysts comparable to their acetate counterparts for the formation of oxazolines from esters and amino alcohols.

## Introduction

Green chemistry has exploded in recent years with a push to move from precious metal catalysts to earth-abundant metals and/or metal-free catalysts.^1,2^ This movement has demanded the development of new starting materials for catalyst precursors to meet the earth abundant, nontoxic goals that underlie green chemistry. To this end, Lewis acid catalysis has come to the forefront of green chemistry.^3^ In many respects, Lewis acid catalysts and the reactions they catalyze have become as varied and impressive as their precious-metal counterparts.^4,5^ An important element in the realm of Lewis acid catalysis is zinc because of its natural abundance and minimal toxicity.^6^ Further, its redox-innocence and geometric indifference provide both a predictable and tunable primary coordination environment—attributes that allow a blank canvas for catalyst design.

Zinc acetates (e.g., Zn(OAc)_2_(H_2_O)_2_ or Zn(O_2_CCF_3_)_2_(H_2_O)_*x*_) are common, commercially available precursors in zinc coordination chemistry but may exist in polymeric, solvate, and/or cluster forms: [Zn(O_2_CR)_2_)]_n_, Zn(O_2_CR)_2_(L)_2_ (L = neutral ligand), and Zn_4_O(O_2_CR)_6_, respectively. For a given carboxylate, it can be possible to obtain all three distinct forms.^7,8^ Unfortunately, synthetic control and predictability over speciation are generally lacking and are likely influenced by solvent, temperature, carboxylate basicity, and reagents used. While control over speciation may be irrelevant for certain applications, when applied to Lewis acid catalysis, speciation has been identified as a key consideration. For example, the well-defined cluster, Zn_4_O(O_2_CCF_3_)_6_, proved to be a 1.5× more active catalyst than polymeric Zn(O_2_CCF_3_)_2_(H_2_O)_*x*_ for a tandem condensation-cyclization to produce oxazolines directly from esters.^9^ Further predictive control over zinc carboxylate speciation could increase the utility of zinc carboxylates as precursors in coordination chemistry, enable the design of more efficient Lewis acid catalysts, and influence macromolecular design strategies.

**Figure 1:**
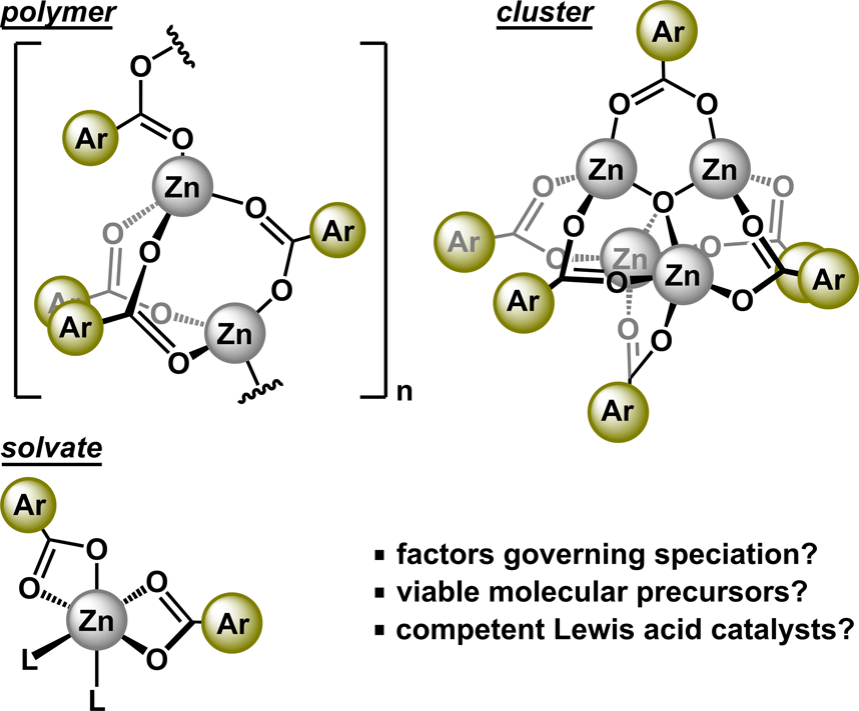
Commonly encountered forms of zinc benzoates and the goals of this study.

Despite having the distinct advantage of wide Hammett-type tunability,^10^ zinc benzoates are not as commonly employed as their acetate analogues as coordination chemistry precursors or as Lewis acid catalysts. Within the macromolecular assembly arena, the Zn_4_O moiety of the Zn_4_O(O_2_CAr)_6_ cluster is a ubiquitous node while the aryl substituents provide nearly endless possibilities for linker design.^11−13^ Our laboratory required well-defined, electronically diverse zinc benzoate precursors and identified polymeric species, [Zn(O_2_CAr)_2_]_n_, as desirable targets. Unfortunately, the literature protocol did not clearly delineate which factors favored the generation of polymeric, solvated, or cluster forms of the benzoates and whether these forms varied as a function of purification/crystallization. For example, intimately related forms of the parent zinc benzoate [Zn(O_2_CPh)_2_]_*n*_, Zn_4_O(O_2_CPh)_6_, Zn(O_2_CPh)_2_(H_2_O)_2_, [Zn_2_(O_2_CPh)_3_(OH)]_*n*_, and Zn_4_O(O_2_CPh)_6_(H_2_O)(THF) all share related synthetic protocol,^7,14−18^ obfuscating a general route to furnish solely [Zn(O_2_CAr)_2_]_*n*_ across a wide range of benzoates.^19^ Therefore, we sought to (1) develop a robust synthesis for polymeric [Zn(O_2_CAr)_2_]_*n*_, (2) define the factors governing their speciation (polymeric vs cluster), and (3) establish their utility in coordination chemistry and Lewis acid catalysis in analogy to their zinc acetate counterparts (Figure 1).

## Results and Discussion

We hypothesized that zinc benzoates would provide a new avenue to generate coordination chemistry precursors and Lewis acid catalysts with an element of precise electronic tuning. Studies were initiated by developing a simple reaction protocol to generate electronically distinct zinc benzoate species. Treating an aqueous solution of ZnSO_4_·7H_2_O with in situ generated sodium benzoate resulted in immediate precipitation of a white solid suspected to be polymeric [Zn(O_2_CPh)_2_]_n_ (**1-H**). The linear zinc benzoate polymer is well-documented and has been studied structurally and spectroscopically.^7,14,15^ Repeating this protocol with *para*-substituted benzoic acids (**1-R**: *p-*NO_2_, *p*-CF_3_, *p-*Br, *p*-CH_2_Cl *p-*H, *p-*CH_3_, *p-*OMe, and *p-*NMe_2_) resulted in analogous results. The protocol was successfully extended to aliphatic carboxylates (pivalic acid),^7^ but failed for aromatic dicarboxylic acids (isophthalic acid).^20^ Each **1-R** species was investigated by ^1^H NMR and ^13^C NMR spectroscopies (DMSO-*d*_6_; 25 °C) and revealed simple spectra containing a single set of resonances for the benzoate. The diagnostic ^13^C NMR carbonyl resonances (*C*=O) in **1-R** trends with the identity of the *para*-substituent, with the most electron donating *p*-OMe shifted furthest downfield (172.29 ppm) and the most electron withdrawing (*p*-NO_2_) furthest upfield (169.53 ppm; Figure S18).^21^ The polymeric zinc benzoates have poor solubility; strong Lewis bases and/or coordinating solvents are known to break apart the polymeric form into discrete species of the type Zn(O_2_CR)_2_(L)_2_ (L = Lewis base) and in some cases, this can be reversible.^22^ The NMR analysis above in DMSO-*d*_6_, therefore, is likely of species of the type Zn(O_2_CR)_2_(DMSO)_*n*_ rather than of the polymer.^23,24^

Complexes **1-R** were investigated further by infrared spectroscopy in an effort to understand their structural makeup (ATR, neat). Previously, Straughan and co-workers described detailed infrared analysis of the discrete forms of [Zn(O_2_CPh)_2_]_*n*_ (**1-H**) and Zn_4_O(O_2_CPh)_6_ (**2-H**) as a means to distinguish between the polymeric and cluster forms of the zinc benzoates.^7^ Importantly, none of the species **1-R** show an O–H absorption that is diagnostic of the hydrate (i.e., Zn(O_2_CPh)_2_(H_2_O)_2_; Figure S32). Our analysis of **1-H** matches the data previously reported for the linear polymer.^25^

**Figure 2:**
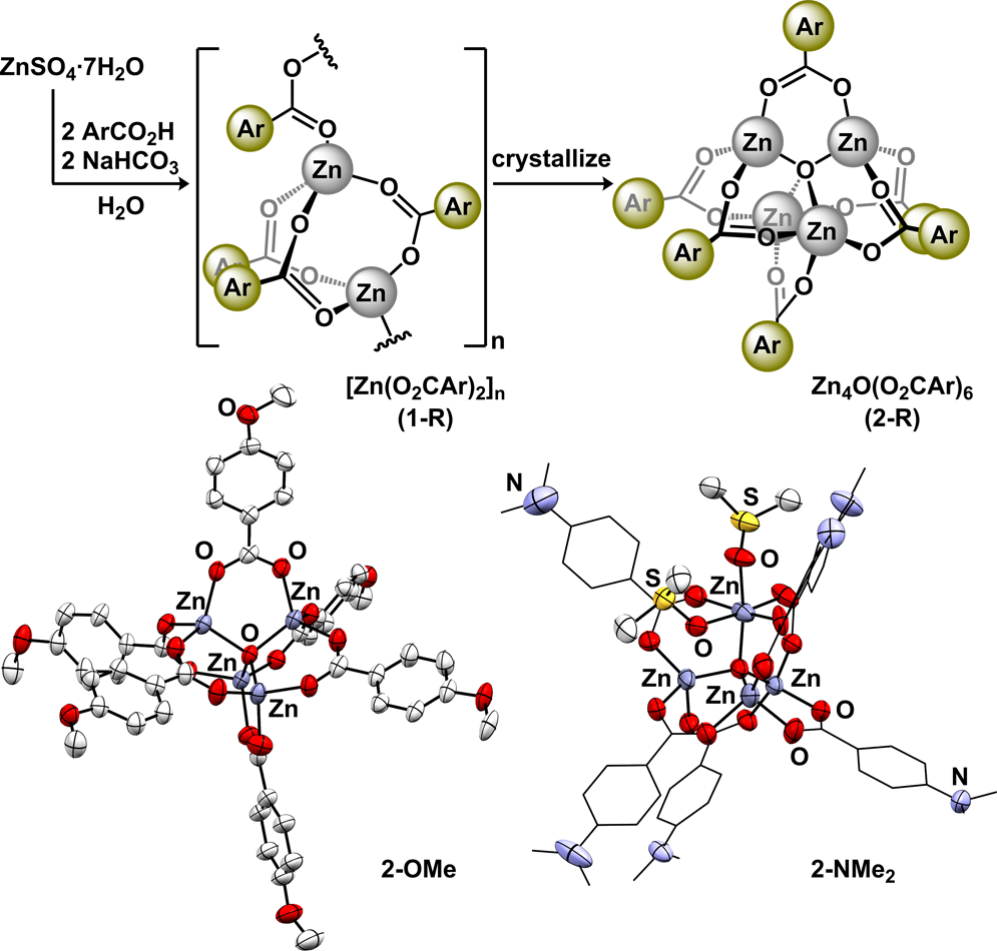
Synthesis of compound **1-R**. Molecular structures of **2-OMe** and **2-NMe**_**2**_ displayed with 50% probability ellipsoids. All H atoms are omitted. In **2-NMe**_**2**_, the aromatic rings are displayed in wireframe for improved clarity.

We sought to glean structural information about complexes **1-R** through X-ray diffraction experiments. We analyzed the two zinc species bearing the most electron-donating benzoates: *p-*OMe and *p*-NMe_2_. Single, X-ray quality crystals were obtained by diffusion of hexanes into a toluene solution of **1-OMe** at room temperature. Data collection and refinement revealed the cluster form, Zn_4_O(O_2_CAr)_6_ (**2-OMe**; Figure 2).^26^ The structure is defined by pseudo tetrahedral symmetry about a central μ_4_-oxo (Zn–O 1.935(5)-1.954(5) Å). Each of the six benzoate ligands spans two zinc atoms with a narrow range of Zn–O bond distances (1.935(5)–1.955(5) Å). The geometry of each zinc atom is identical and is best described as tetrahedral (τ_4_ = 0.97).^27^ The structure of **2-OMe** is similar to those of previously reported complexes (see Table S1 for literature complexes).

Whereas single crystals of **2-OMe** were obtained from a noncoordinating solvent, single crystals of **2-NMe**_**2**_ obtained from a dimethyl sulfoxide solution revealed a doubly solvated version of the cluster, Zn_4_O(O_2_CAr)_6_(DMSO)_2_.^28^ The solvate of **2-NMe**_**2**_ contains four zinc atoms bridged by six benzoate ligands with a central μ_4_-oxo. The solvation by DMSO renders decreased molecular symmetry (C_s_), where three zinc atoms are tetrahedral (τ_4_ = 0.82–0.94), and a lone zinc is octahedral (Figure 2). The two O-bound DMSO ligands (Zn–O = 2.047(3), 2.184(3) Å) are cis (86.34(12)°). The three tetrahedral zinc atoms display shorter bond distances to the central μ_4_-oxo (Zn–O_ave_ = 1.909(6) Å) than to the octahedral zinc (Zn–O = 1.988(3) Å). Similar solvated clusters of the type Zn_4_O(O_2_CAr)_6_(L)_2_ have been observed for L = H_2_O, THF, and DMSO.^18,29^ We were unable to crystallize any other forms of zinc benzoate species derived from **1-OMe** and **1-NMe**_**2**_ (i.e., Zn(O_2_CAr)_2_(L)_2_ or [Zn(O_2_CAr)_2_]_*n*_).

**Figure 3:**
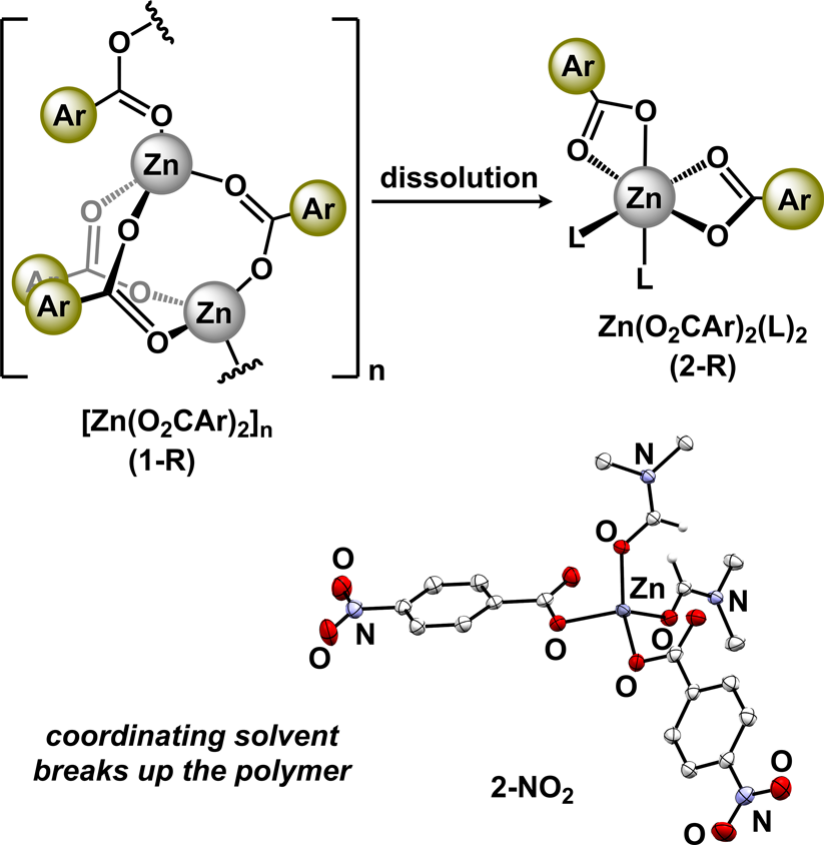
Solvation of **1-NO**_**2**_ with coordinating solvents generates Zn(O_2_CAr)_2_(dmf)_2_ (**2-NO**_**2**_). Molecular structure displayed with 50% probability ellipsoids. All H atoms, except those attached to carbonyl moieties, are omitted for clarity.

Monomeric units of the type Zn(O_2_CAr)_2_(L)_2_ are established to be obtainable from polymeric zinc benzoates by treatment with stoichiometric equivalents of strong Lewis bases. Our attempts to structurally characterize **1-NO**_**2**_, which contains the least Lewis basic benzoate of the series, resulted in the isolation of the monomeric disolvate. Analysis of single crystals of **1-NO**_**2**_ obtained from a *N*,*N*-dimethylformamide (DMF) solution revealed Zn(O_2_CAr)_2_(DMF)_2_ (**2-NO**_**2**_, Figure 3) that is reminiscent of the previously reported dihydrate structure, Zn(O_2_CAr)_2_(H_2_O)_2_.^30^ Each benzoate ligand displays a short (1.9647(8) Å) and a long (2.725 Å) contact with zinc and can be considered either a tetrahedral (τ_4_ = 0.83) or distorted octahedral geometry. The DMF ligands display Zn–O distances (1.9681(7) Å) that are shorter than those observed in similar halide complexes, (DMF)_2_ZnX_2_.^31,32^ Despite repeated attempts, we were unable to crystallize any other forms of zinc benzoate species (i.e., Zn_4_O(O_2_CAr)_6_ or [Zn(O_2_CAr)_2_]_n_) derived from **1-NO**_**2**_.

**Figure 4:**
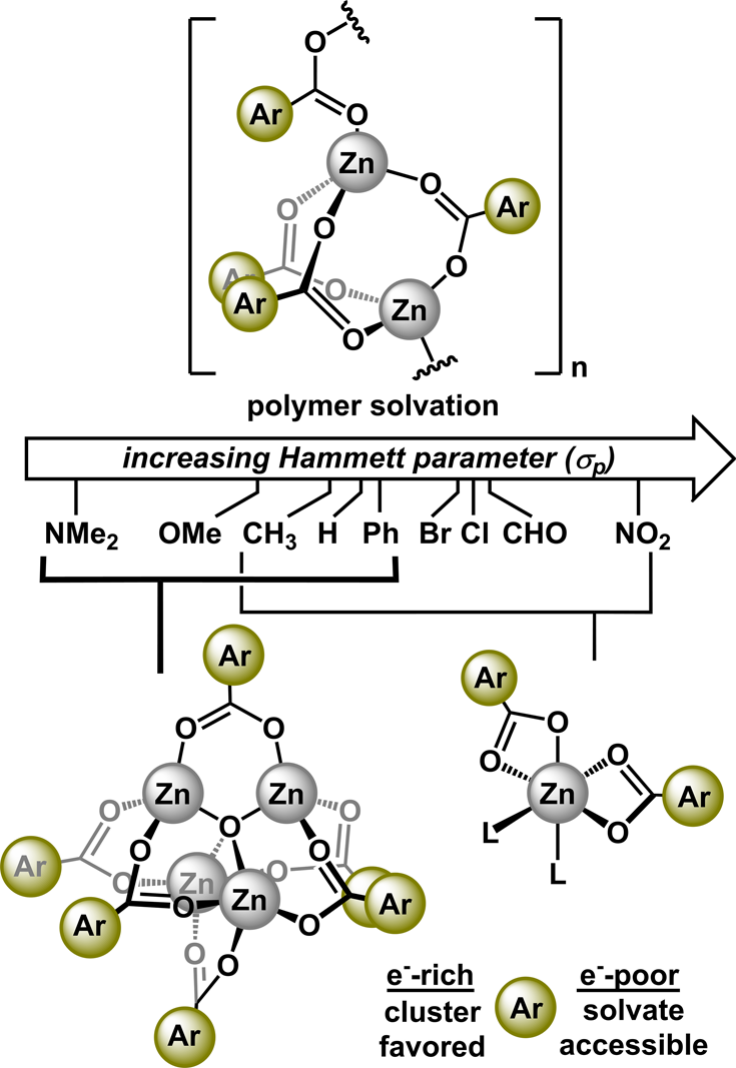
Relationship between Hammett parameter (σ_p_) and favorability of forming the cluster versus monomeric solvate.

**Figure 5:**
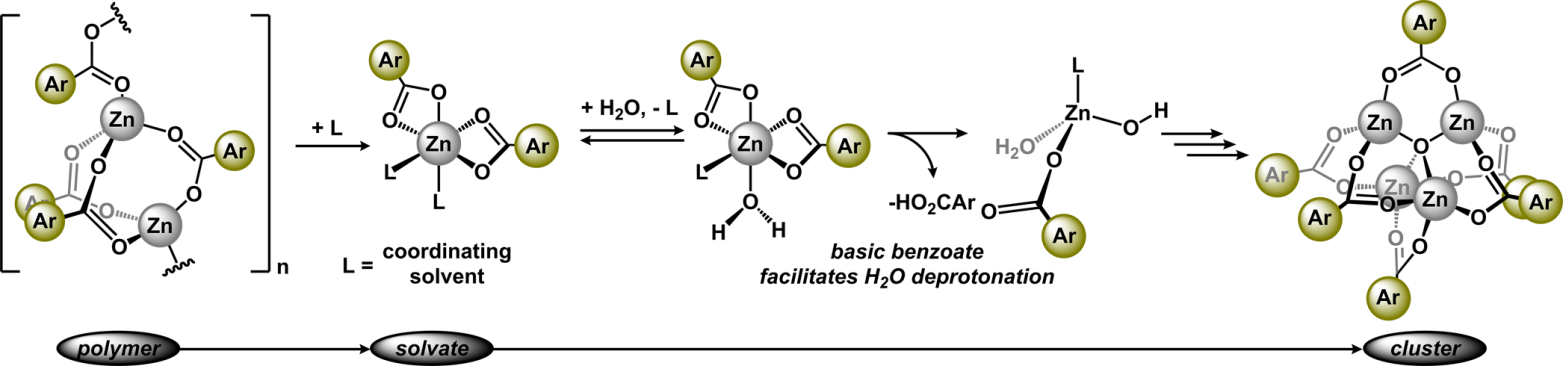
Proposed formation of clusters, Zn_4_O(O_2_CAr)_6_, from polymeric [Zn(O_2_CAr)_2_]_*n*_.

Our analyses suggest that the zinc benzoates formed under our reaction conditions are of the polymeric form, [Zn(O_2_CAr)_2_]_*n*_. Disparate structural analyses between **2-NO**_**2**_ and **2-OMe/NMe**_**2**_ suggest that, when dissolved, the polymeric species can generate two species, the mononuclear solvate, Zn(O_2_CAr)_2_(L)_2_, and/or the cluster, Zn_4_O(O_2_CAr)_6_. Qualitatively, the cluster is favored when the benzoate is strongly electron-donating (*p*-OMe, *p*-NMe_2_), and the mononuclear solvate is formed when the benzoate is electron-withdrawing (*p*-NO_2_). While the mononuclear species can be explained by simple solvation, the Zn_4_O(O_2_CAr)_6_ cluster must be derived from acid/base reactivity involving H_2_O. Figure 5 details a plausible reaction pathway to generate the cluster. The key step for the generation of the μ_4_-oxo involves deprotonation of water by the benzoate. This reversible deprotonation will be facilitated by increasingly basic benzoates (i.e., *p*-OMe, *p*-NMe_2_). Therefore, as the benzoate becomes more electron-donating, the propensity to generate the Zn_4_O(O_2_CAr)_6_ cluster should increase. To substantiate this hypothesis, we tabulated structurally characterized examples of Zn(O_2_CAr)_2_(H_2_O)_2_ and Zn_4_O(O_2_CAr)_6_ (only *para*-substituted benzoates were considered) as a function of their Hammett parameter (σ_p_) (Figure 4, Table S1).^10^ From this analysis, we observed a clear trend where the Zn_4_O(O_2_CAr)_6_ cluster has only been reported for benzoates with σ_p_ ranging from +0.06 to −0.83.^7,33,34^ The mononuclear solvate has been reported over a range of σ_p_ = −0.17 to +0.78.^14,29,30,35−40^ These data clearly indicate that when σ_p_ is positive, stable formation of the hydrate, Zn(O_2_CAr)_2_(H_2_O)_2_, can persist, whereas, if σ_p_ is negative, the tetranuclear cluster is accessible. This simple Hammett trend enables predictability with respect to speciation and can be a useful tool for guiding macromolecular assemblies.

**Figure 6:**
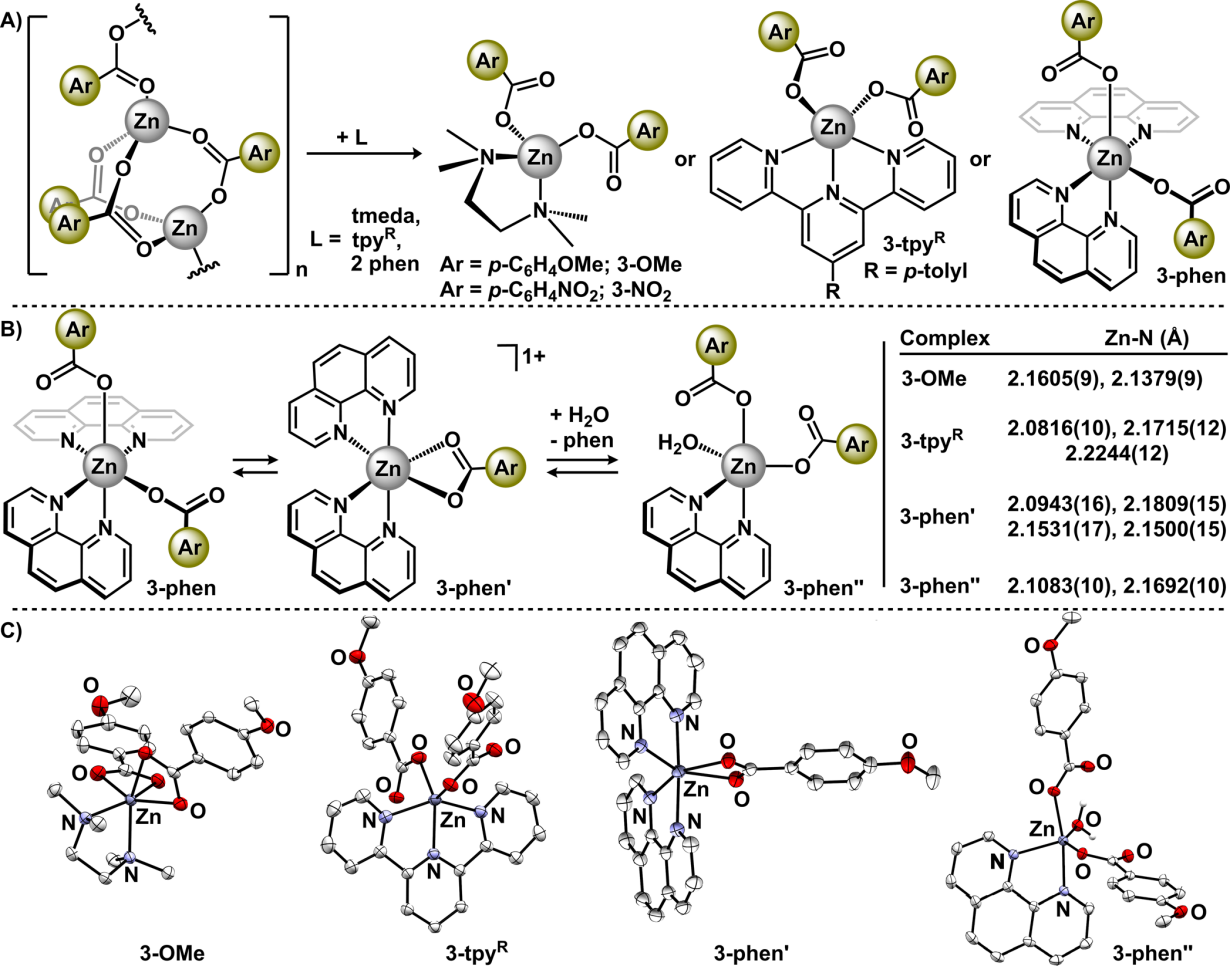
Utility of zinc benzoates as precursors for four-, five-, and six-coordinate complexes. (A) Synthesis of **3-OMe**, **3-NO2**, **3-tpy**^**R**^, and **3-phen**. (B) Proposed dynamic behavior of **3-phen** and comparison of Zn–N bond distances. (C) Molecular structures of **3-OMe**, **3-tpy**^**R**^, **3-phen′**, and **3-phen″** displayed with 50% probability ellipsoids. For clarity, all H atoms are omitted unless they are connected to a heteroatom.

With an understanding of how each zinc benzoate species forms, we wanted to discern whether any reactivity differences would arise for electron-donating versus electron-withdrawing benzoates. Our initial efforts focused on the coordination chemistry of **1-NO**_**2**_ and **1-OMe** using ubiquitous inorganic/organometallic ancillary ligands. Treating **1-NO**_**2**_ or **1-OMe** with a single equivalent of *N*,*N*,*N*′,*N*′-tetramethylethylenediamine (tmeda) resulted in the formation of (tmeda)Zn(O_2_CAr)_2_ (**3-NO**_**2**_ and **3-OMe**) in high isolated yields (Figure 6A). The two species display vastly different solubility in common solvents: **3-OMe** is soluble while **3-NO**_**2**_ is not soluble in common NMR solvents (CDCl_3_, DMSO-*d*_6_) for routine analysis. **3-OMe** displays a ^1^H NMR spectrum consistent with a 2:1 ratio of benzoate:tmeda. Single crystal X-ray diffraction studies of each revealed six-coordinate zinc complexes where the benzoate ligands are κ^2^-coordinated and display both a short and a long Zn–O contact for each ligand (**3-OMe** is displayed in Figure 6C; 3**-NO**_**2**_ is displayed in Figure S53). The Zn–O distances in **3-OMe** display a relatively narrow range (2.0918(9)–2.2573(9) Å) but are markedly different than related (en)Zn(O_2_CAr)_2_ (1.939–2.782 Å; Ar = *p*-C_6_H_5_OMe; en = ethane-1,2-diamine).^41^ When only considering the short Zn–O contact of each benzoate, the complexes are best described as pseudotetrahedral (τ_4_: **3-NO**_**2**_ = 0.77; **3-OMe** = 0.72). These data suggest that **1-R** is a suitable precursor for coordination complexes regardless of the donor properties of the benzoate ligand.

To systematically assess the utility of **1-R** as a molecular precursor, we targeted four-, five- and six-coordinate complexes from **1-OMe** due to its higher solubility. Treating methanol solutions of **1-OMe** with one equivalent of 4′-(4-methylphenyl)-2,2’:6′,2″-terpyridine (tpy^R^) or two equivalents of 1,10-phenanthroline (phen) produced (tpy^R^)Zn(O_2_CAr)_2_ (**3-tpy**^**R**^) and (phen)_2_Zn(O_2_CAr)_2_ (**3-phen**), respectively. ^1^H NMR spectroscopy (CDCl_3_, 25 °C) was consistent with the proposed formulas as **3-tpy**^**R**^ revealed a 1:2 ratio of tpy^R^:benzoate, whereas, **3-phen** revealed a 1:1 ratio of phen:benzoate. Structural confirmation of **3-tpy**^**R**^ was achieved by the analysis of single crystals obtained by diffusing hexanes into a CH_2_Cl_2_ solution at room temperature (Figure 6C). Data refinement revealed a five-coordinate zinc complex in a distorted square planar geometry imparted by the tpy^R^ ligand (τ_5_ = 0.31) with each benzoate ligand best described as κ^1^ (Zn–O = 1.9831(10) Å).^42^ The structure of **3-tpy**^**R**^ is comparable to (tpy^Ph^)Zn(O_2_CPh)_2_ (tpy^Ph^ = 4′-phenyl-2,2’:6′,2″-terpyridine) derived from hydrothermal synthesis.^16^ Crystal growth of **3-phen** (1,2-C_2_H_4_Cl_2_/hexanes, room temperature) afforded two different crystalline materials that were analyzed in separate experiments: [(phen)_2_Zn(O_2_CAr)]^+^ (**3-phen**′****) and (phen)Zn(O_2_CAr)_2_(H_2_O) (**3-phen**′**′******). The two species, **3-phen**′**** and **3-phen**′**′******, are likely a function of the crystallization conditions and can be ascribed to the coordination indifference of d^10^-zinc, particularly in the presence of ambidentate benzoate ligands.^43,44^Figure 6B provides a plausible pathway through which each species can form from **3-phen**. A related compound, (phen)Zn(O_2_CPh)_2_ has been crystallographically characterized and demonstrates that both benzoate ligands can display κ^2^ coordination modes in the absence of trace H_2_O.^45^ Unfortunately, we have been unable to obtain single X-ray-quality crystals under different conditions.

The diffraction experiment of **3-phen**′**** revealed two asymmetric molecules per unit cell. While structurally similar, the Zn–O bond distances vary considerably, where one molecule displays one short and one long distance (2.0227(14) and 2.457(2) Å); the other molecule shows nearly identical Zn–O bonds (2.2202(13) and 2.1672(12) Å). These observations provide further support of the lability of the benzoate ligands in **3-phen** and their propensity to exchange between κ^1^- and κ^2^-coordination modes. Benzoate ligand lability in Zn_4_O(O_2_CPh)_6_ has previously been described during metal–organic framework formation,^46^ and polydentate pyridyl ligands in six-coordinate zinc complexes are known to be labile.^47−49^ The structure of **3-phen”** is formally derived via displacement of phen with a water molecule (Zn–OH_2_ = 2.0297(9) Å) to generate a 5-coordinate complex (τ_5_ = 0.31). A related structure, (phen)Zn(O_2_CPh)_2_(H_2_O), was obtained by heating a water/alcohol mixture of the reaction components.^50^ The stability of **3-phen**′**′****** is likely derived from a moderate-strength hydrogen bond between the H_2_O ligand and benzoate oxygen (O_benzoate_–O_H2O_ = 2.577 Å).^51^

The generation of complexes **3** demonstrated that **1-R** is a viable precursor to coordination complexes, in analogy to their acetate analogues; however, an additional utility of simple zinc acetates is their ability to act as Lewis acid catalysts. Whereas zinc acetates are generally limited to acetate or trifluoroacetate, enhanced precision for electronic tuning is available to benzoates. Electronic tuning is an important parameter for Lewis acid-induced reactivity^52^ and has been demonstrated in catalytic studies employing Zn(O_2_CCH_3_)_2_ and Zn(O_2_CCF_3_)_2_.^9^ To the best of our knowledge, simple zinc benzoates, such as **1-R**, have yet to be employed for Lewis acid catalysis.

**Figure 7:**
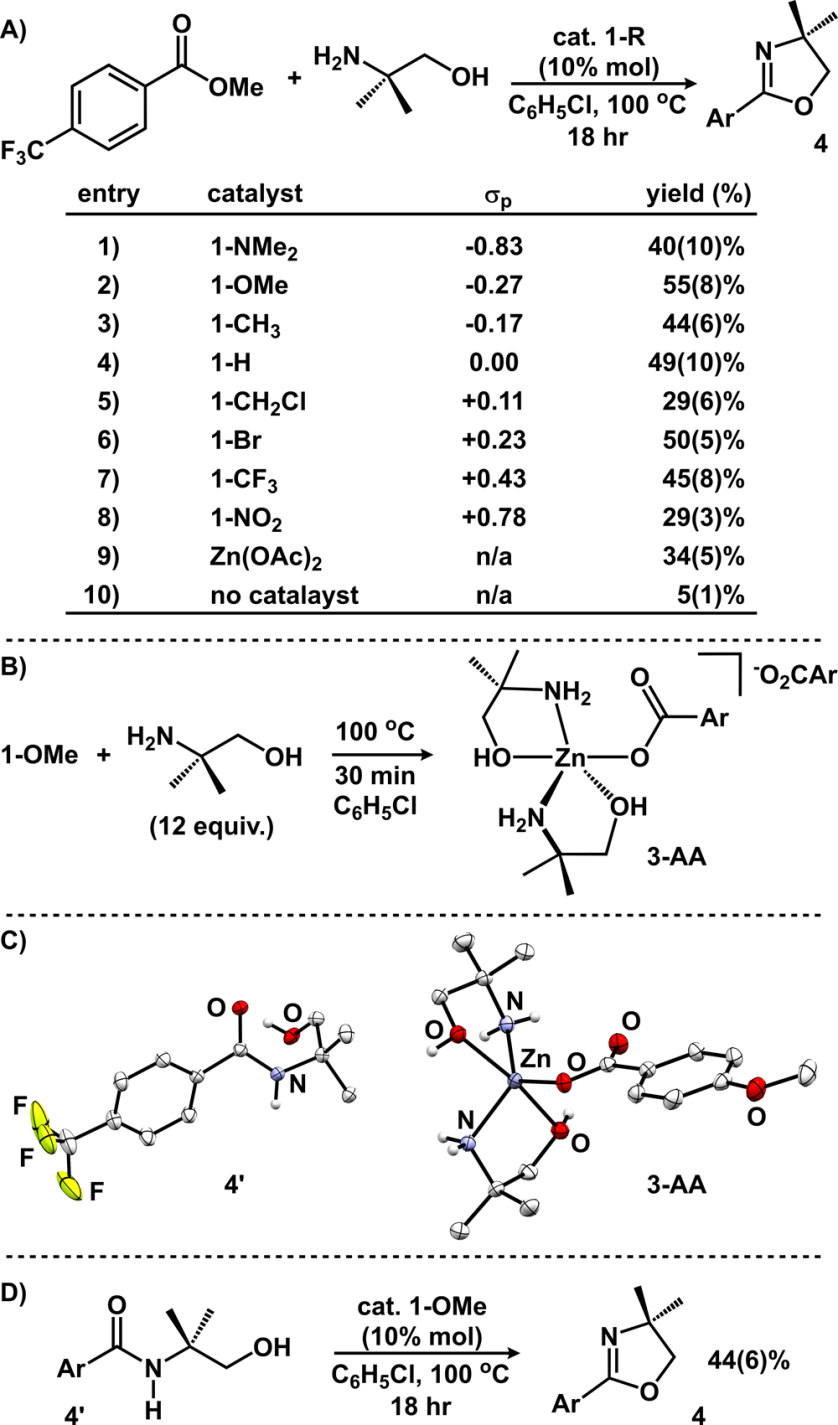
(A) Summary of catalytic trials to form oxazoline **4** with catalysts **1-R**. Yields of **4** were determined by ^19^F NMR spectroscopy. Values in parentheses are standard deviations. (B) Generation of **3-AA** from **1-OMe**. (C) Molecular structures of **4**′**** and **3-AA** displayed with 50% probability ellipsoids. All H atoms except those attached to heteroatoms have been omitted for clarity. In **3-AA**, the benzoate counteranion is omitted for clarity. (D) Catalytic cyclization of **4**′**** to generate **4**.

To assess the viability of **1-R** as Lewis acid catalysts, we targeted oxazoline formation through a tandem condensation-cyclodehydration reaction that has been previously established for acetate variants.^9,53^ We hypothesized three potential outcomes: (1) catalytic success would trend with the benzoate Hammett parameters, (2) a stark difference would be observed for species capable of forming clusters (σ_p_ < +0.07) versus species that cannot (σ_p_ ≥ +0.07), or (3) catalytic performance would depend on the inherent solubility properties of **1-R**. Catalytic trials were unoptimized and mirrored reaction conditions employed by Ohshima and co-workers: methyl 4-(trifluoromethyl)benzoate and 2-amino-2-methyl-1-propanol were reacted with 10 mol % catalyst (**1-R**) in C_6_H_5_Cl at 100 °C.^9^ The yield of 4,4′-dimethyl-2-(4-trifluoromethyl)phenyl-4,5-dihydrooxazole (**4**) was assessed by ^19^F NMR. The results, displayed in Figure 7A, reveal modest catalysis with yields of **4** ranging from 29 to 55%. The yields for this reaction are comparable to those previously observed for ZnO, ZnX_2_ (X = Cl, OAc, O_2_CCF_3_), and Cd(O_2_CCF_3_)_2_.^9^ Importantly, all catalysts displayed improved competency for the formation of oxazoline **4** compared with the control reactions without catalyst (5% yield; entry 10). Additionally, we assessed commercially available anhydrous zinc acetate (CAS 557–34–6; entry 9), which is established to adopt a polymeric structure of the type [Zn(OAc)_2_]_n_ in analogy to **1-R**, and found that it performed similarly to the zinc benzoates for the formation of **4**.^54,55^

The modest reaction yields for all catalysts are attributed to the inefficiency of these species to affect the intramolecular ring closing of the intermediate β-hydroxy amide: upon cooling catalytic trials to room temperature, the gradual precipitation of *N*-(2-hydroxy-1,1-dimethylethyl)-4-(trifluoromethyl)benzamide (**4**′****) was observed and identified by ^1^H NMR spectroscopy^56^ and X-ray diffraction studies (Figure 7C). Importantly, **4**′**** was observed for every catalytic reaction but was not observed for the control reaction (entry 10). Subjecting isolated **4**′**** to intramolecular ring closing with **1-OMe** under analogous catalytic conditions produced oxazoline **4** in 44% yield (Figure 7D).

In no instances were catalysts **1-R** observed to fully dissolve *prior to heating*. This is consistent with the polymeric nature of these species particularly in noncoordinating chlorobenzene solvent. Upon heating, the catalysts dissolve presumably due to interactions with the Lewis basic reaction components (i.e., ester, amino alcohol) in analogy to the syntheses of compounds **3**, suggesting a homogeneous reaction.^57^ We probed this by performing control experiments with the individual reaction components: **1-OMe** was separately treated with ester and amino alcohol. While there was no evidence of the ester irreversibly interacting with the catalyst in solution, ^1^H NMR spectroscopy suggested that the amino alcohol is capable of interacting with the catalyst—and presumably facilitates dissolution. Separate experiments with *tert*-butylamine and *n*-propyl alcohol revealed that *both* Lewis basic components of the amino alcohol, the amine and the alcohol, can interact with the zinc catalyst. We were able to isolate single, X-ray quality crystals from the reaction between **1-OMe** and the amino alcohol (Figure 7B). Data refinement revealed a cationic five-coordinate (τ_5_ = 0.68) zinc coordinated to two amino alcohol ligands, [(κ-N,O-NH_2_C(CH_3_)_2_CH_2_OH)_2_Zn(O_2_CAr)][O_2_CAr] (**3-AA**; Figure 7C). In the mononuclear complex, each amino alcohol is κ^2^-coordinated through the alcohol and the amine while the benzoate ligand is best described as monodentate (Zn–O = 1.9570(9) vs 2.894 Å).^58^ The outer sphere benzoate engages in intermolecular H-bonding interactions and does not interact with zinc. The molecular structure of **3-AA** provides unique insight into the potential binding modes that impart high stereoselectivity in addition reactions employing dialkylzinc and chiral amino alcohol additives.^59−62^

In the catalytic formation of **4**, the structure of **3-AA** suggests that a mononuclear pathway cannot be discounted. Irrespective of nuclearity, the reaction likely follows typical Lewis acid catalyzed pathways: activation of the ester is followed by nucleophilic attack by the amino alcohol to form **4**′****. This model for Lewis acid-catalyzed addition reactions has previously been invoked for polynuclear zinc clusters^22^ and other multimetallic systems.^63,64^ Whereas catalysts **1-R** are inefficient in affecting the intramolecular cyclization of β-hydroxy amide **4**′****, highly efficient catalysts are known.^65−67^ Despite the modest catalytic success under these conditions, these data highlight that zinc benzoates can be viable Lewis acid catalysts.

## Conclusions

We described a robust synthesis for electronically diverse polymeric zinc benzoates of the type [Zn(O_2_CAr)_2_]_*n*_. When dissolved, these species can form mononuclear solvates, Zn(O_2_CAr)_2_(H_2_O)_2_, or tetranuclear clusters, Zn_4_O(O_2_CAr)_6_. The propensity for solvate versus cluster formation is dictated by the electron donor properties of the benzoate and shows a correlation with their Hammett parameter (σ_p_). Regardless of their electronic identity, these zinc benzoates can be readily utilized as precursors for 4-, 5-, and 6-coordinate metal complexes. Comparable to their acetate analogues, the zinc benzoates are competent Lewis acid catalysts and this has been demonstrated for the conversion of esters and amino alcohols to oxazolines. These studies lay the groundwork for elevating zinc benzoates to their acetate counterparts, and future work is aimed at implementing these species into systems where electronic fine-tuning is a necessity.

## Experimental Section

### General Synthesis: Formation of [Zn(O_2_CAr)_2_]_*n*_ from ZnSO_4_·7H_2_O, NaHCO_3_, and ArCO_2_H

A 250 mL Erlenmeyer flask was charged with a benzoic acid (34.779 mmol), a stir bar, and 50 mL of deionized water. While stirring, a solution of sodium bicarbonate (2.921 g, 34.770 mmol) in deionized water (15 mL) was added, resulting in gas evolution. After 30 min, the mixture was filtered via vacuum filtration to remove the insoluble particulates. The filtrate was slowly added to a stirring solution of ZnSO_4_·7H_2_O (5.000 g, 17.389 mmol) in deionized water (25 mL), resulting in the rapid precipitation of a white powder. The reaction was cooled to 0 °C and stirred for an additional 30 min. The solid was collected by vacuum filtration, washed with 100 mL of deionized water, and dried in vacuo at room temperature until the mass of the material no longer changed (8–24 h).

### General Catalytic Procedure for Synthesis of 4,4′-Dimethyl-2-(4-trifluoromethyl)phenyl-4,5-dihydrooxazole

All catalytic trials were set up open to the air. A 20 mL scintillation vial was charged with a catalyst (0.060 mmol; 10 mol %) and a stir bar. Via volumetric pipets, methyl 4-(trifluoromethyl)benzoate (0.500 mL of a 1.196 M stock solution in C_6_H_5_Cl; 0.598 mmol) and 2-amino-2-methyl-1-propanol (0.500 mL of a 1.439 M stock solution in C_6_H_5_Cl; 0.720 mmol; 1.2 equiv) were added. The vials were sealed with a Teflon-lined cap and stirred in an aluminum heating block at 100 °C for 18 h. The samples were cooled to RT and α,α,α,-trifluoroanisole (0.500 mL of a 1.196 M stock solution in C_6_H_5_Cl, 0.598 mmol) was added via a volumetric pipet as an internal standard. The solution was filtered to remove insoluble colorless/white particulates and analyzed by ^19^F NMR. A minimum of four trials were performed for each catalyst.

## Supporting Information Available

Full experimental procedures and spectroscopic characterization of all species and crystal structure data (PDF)
