# Supplementary material for: Defining the Speciation, Coordination Chemistry, and Lewis Acid Catalysis of Electronically Diverse Zinc Benzoates
Source: Organometallics. 2024 Dec 16;44(1):46–53. doi: 10.1021/acs.organomet.4c00358 (PMC11734107; doi:10.1021/acs.organomet.4c00358)
Supplement: Supplementary file 1 — om4c00358_si_001.pdf [file om4c00358_si_001.pdf]

Supporting Information for:

Defining the Speciation, Coordination Chemistry, and Lewis Acid Catalysis of Electronically Diverse Zinc Benzoates

*Lydia A. Dunaway,[a] Audrey G. Davis,[a] Victoria J. Carter,[a] Albert K. Korir,[a] Matthias Zeller,[b] and John J. Kiernicki[a]**

[a]Department of Chemistry, Drury University, Springfield, Missouri 65802, United States

[b]H.C. Brown Laboratory, Department of Chemistry, Purdue University, West Lafayette, Indiana 47907, United States

General Considerations S4-S5
Experimental Procedures S5-S10
Table S1 Literature comparison of $(L)_2Zn(O_2CAr)_2$ and $Zn_4O(O_2CAr)_6$ S11
Scheme S1 Literature protocol for synthesis of zinc benzoates S11
Figure S1 ^{1}H NMR spectrum of $[Zn(O_2CAr)_2]_n$ (Ar = C_6H_5) S12
Figure S2 ^{13}C{^{1}H} NMR spectrum of $[Zn(O_2CAr)_2]_n$ (Ar = C_6H_5) S12
Figure S3 ^{1}H NMR spectrum of $[Zn(O_2CAr)_2]_n$ (Ar = *p*-$C_6H_4NO_2$) S13
Figure S4 ^{13}C{^{1}H} NMR spectrum of $[Zn(O_2CAr)_2]_n$ (Ar = *p*-$C_6H_4NO_2$) S13
Figure S5 ^{1}H NMR spectrum of $[Zn(O_2CAr)_2]_n$ (Ar = *p*-C_6H_4Br) S14
Figure S6 ^{13}C{^{1}H} NMR spectrum of $[Zn(O_2CAr)_2]_n$ (Ar = *p*-C_6H_4Br) S14
Figure S7 ^{1}H NMR spectrum of $[Zn(O_2CAr)_2]_n$ (Ar = *p*-$C_6H_4CH_3$) S15
Figure S8 ^{13}C{^{1}H} NMR spectrum of $[Zn(O_2CAr)_2]_n$ (Ar = *p*-$C_6H_4CH_3$) S15
Figure S9 ^{1}H NMR spectrum of $[Zn(O_2CAr)_2]_n$ (Ar = *p*-C_6H_4OMe) in $CDCl_3$ S16
Figure S10 ^{13}C{^{1}H} NMR spectrum of $[Zn(O_2CAr)_2]_n$ (Ar = *p*-C_6H_4OMe) in $CDCl_3$ S16
Figure S11 ^{1}H NMR spectrum of $[Zn(O_2CAr)_2]_n$ (Ar = *p*-C_6H_4OMe) in dmso-d_6 .... S17
Figure S12 ^{13}C{^{1}H} NMR spectrum of $[Zn(O_2CAr)_2]_n$ (Ar = *p*-C_6H_4OMe) in dmso-d_6 .... S17
Figure S13 ^{1}H NMR spectrum of $[Zn(O_2CAr)_2]_n$ (Ar = *p*-$C_6H_4NMe_2$) S18
Figure S14 ^{1}H NMR spectrum of $[Zn(O_2CAr)_2]_n$ (Ar = *p*-$C_6H_4CF_3$) S18
Figure S15 ^{13}C{^{1}H} NMR spectrum of $[Zn(O_2CAr)_2]_n$ (Ar = *p*-$C_6H_4CF_3$) S19
Figure S16 ^{1}H NMR spectrum of $[Zn(O_2CAr)_2]_n$ (Ar = *p*-$C_6H_4CH_2Cl$) S19
Figure S17 ^{13}C{^{1}H} NMR spectrum of $[Zn(O_2CAr)_2]_n$ (Ar = *p*-$C_6H_4CH_2Cl$) S20
Figure S18 Overlay of ^{13}C{^{1}H} NMR spectra of $[Zn(O_2CAr)_2]_n$ S20
Figure S19 ^{1}H NMR spectrum of $(tpy^R)Zn(O_2CAr)_2$ (Ar = *p*-C_6H_4OMe) S21
Figure S20 ^{1}H NMR spectrum of $(phen)_2Zn(O_2CAr)_2$ (Ar = *p*-C_6H_4OMe) S21
Figure S21 ^{13}C{^{1}H} NMR spectrum of $(phen)_2Zn(O_2CAr)_2$ (Ar = *p*-C_6H_4OMe) S22
Figure S22 ^{1}H-^{1}H COSY spectrum of $(phen)_2Zn(O_2CAr)_2$ (Ar = *p*-C_6H_4OMe) S22
Figure S23 ^{1}H NMR spectrum of $(tmeda)Zn(O_2CAr)_2$ (Ar = *p*-C_6H_4OMe) S23
Figure S24 ^{13}C{^{1}H} NMR spectrum of $(tmeda)Zn(O_2CAr)_2$ (Ar = *p*-C_6H_4OMe) S23
Figure S25 ^{1}H NMR spectrum of 4,4′-dimethyl-2-(4-trifluoromethyl)phenyl-4,5-dihydrooxazole S24
Figure S26 ^{13}C{^{1}H} NMR spectrum of 4,4′-dimethyl-2-(4-trifluoromethyl)phenyl-4,5-dihydrooxazole .. S24
Figure S27 ^{19}F NMR spectrum of 4,4′-dimethyl-2-(4-trifluoromethyl)phenyl-4,5-dihydrooxazole S25
Figure S28 ^{1}H NMR spectrum of benzamide **4’** in $CDCl_3$ S25
Figure S29 ^{1}H NMR spectrum of benzamide **4’** in dmso-d_6 .... S26
Figure S30 ^{13}C{^{1}H} NMR spectrum of N-(2-hydroxy-1,1-dimethylethyl)-4-(trifluoromethyl)benzamide S26
Figure S31 ^{1}H NMR spectra overlay of benzamide **4’** from catalytic reaction and authentic S27
Figure S32 Infrared spectra of $[Zn(O_2CAr)_2]_n$ S27
Figure S33 Infrared spectrum of $(tpy^R)Zn(O_2CAr)_2$ (Ar = *p*-C_6H_4OMe) S28
Figure S34 Infrared spectrum of $(phen)_2Zn(O_2CAr)_2$ (Ar = *p*-C_6H_4OMe) S28
Figure S35 Infrared spectra of $(tmeda)Zn(O_2CAr)_2$ S29
Figure S36 Infrared spectrum of 4,4′-dimethyl-2-(4-trifluoromethyl)phenyl-4,5-dihydrooxazole S29
Figure S37 Infrared spectrum of N-(2-hydroxy-1,1-dimethylethyl)-4-(trifluoromethyl)benzamide S30
Figure S38 Infrared spectrum of $[Zn(O_2C^tBu)_2]_n$ with literature comparison S30
Table S2 Summary of catalytic reactions S31
Figure S39 Images from catalytic trials at different time points S32
Figure S40 ^{1}H NMR spectra overlay of catalyst with amino alcohol S34
Figure S41 ^{1}H NMR spectra overlay of catalyst with amino alcohol S35
Figure S42 ^{1}H NMR spectra overlay of components of catalytic reaction S36
Figure S43 ^{1}H NMR spectra overlay of catalyst with benzamide **4’** S37

Figure S44 ^{1}H NMR spectra overlay of catalyst with ester S38
Figure S45 ^{1}H NMR spectra overlay of catalyst with *tert*-butylamine S39
Figure S46 ^{1}H NMR spectra overlay of catalyst with *n*-propyl alcohol S40
Figure S47 Infrared spectra (ATR) overlay of catalyst before and after heating S41
Figure S48 Infrared spectra (C_6H_5Cl) overlay of catalyst with and without heating S41
Crystallographic Details S42-S66
Table S3 Experimental parameters for $Zn_4O(O_2CAr)_6$ (Ar = *p*-$C_6H_4OCH_3$) S42
Figure S49 Molecular structure of $Zn_4O(O_2CAr)_6$ (Ar = *p*-$C_6H_4OCH_3$) S43
Table S4 Experimental parameters for $Zn_4O(O_2CAr)_6(dmso)_2$ (Ar = *p*-$C_6H_4NMe_2$) S44
Figure S50 Molecular structure of $Zn_4O(O_2CAr)_6(dmso)_2$ (Ar = *p*-$C_6H_4NMe_2$) S45
Table S5 Experimental parameters for $Zn(O_2CAr)_2(dmf)_2$ (Ar = *p*-$C_6H_4NO_2$) S46
Figure S51 Molecular structure of $Zn(O_2CAr)_2(dmf)_2$ (Ar = *p*-$C_6H_4NO_2$) S47
Table S6 Experimental parameters for (tmeda)$Zn(O_2CAr)_2$ (Ar = *p*-C_6H_4OMe) S48
Figure S52 Molecular structure of (tmeda)$Zn(O_2CAr)_2$ (Ar = *p*-C_6H_4OMe) S49
Table S7 Experimental parameters for (tmeda)$Zn(O_2CAr)_2$ (Ar = *p*-$C_6H_4NO_2$) S50
Figure S53 Molecular structure of (tmeda)$Zn(O_2CAr)_2$ (Ar = *p*-$C_6H_4NO_2$) S51
Table S8 Experimental parameters for $(tpy^R)Zn(O_2CAr)_2$ (Ar = *p*-C_6H_4OMe) S52
Figure S54 Molecular structure of $(tpy^R)Zn(O_2CAr)_2$ (Ar = *p*-C_6H_4OMe) S53
Table S9 Experimental parameters for (phen)$Zn(O_2CAr)_2(H_2O)$ (Ar = *p*-C_6H_4OMe) S54
Figure S55 Molecular structure of (phen)$Zn(O_2CAr)_2(H_2O)$ (Ar = *p*-C_6H_4OMe) S55
Table S10 Experimental parameters for $[(phen)_2Zn(O_2CAr)]^+$ (Ar = *p*-C_6H_4OMe) S56
Figure S56 Molecular structure of $[(phen)_2Zn(O_2CAr)]^+$ (Ar = *p*-C_6H_4OMe) S57
Table S11 Experimental parameters for benzamide **4'** S58
Figure S57 Molecular structure of benzamide **4'** S59
Figure S58 Extended structure of benzamide **4'** S59
Table S12 Experimental parameters for $[\{\kappa\text{-}N,O\text{-}NH_2C(CH_3)_2CH_2OH\}_2Zn(O_2CAr)][O_2CAr]$ S60
Figure S59 Molecular structure of $[\{\kappa\text{-}N,O\text{-}NH_2C(CH_3)_2CH_2OH\}_2Zn(O_2CAr)][O_2CAr]$ S61
Table S13 Metrical parameters for $Zn_4O(O_2CAr)_6$ (Ar = *p*-$C_6H_4OCH_3$) S62-S63
Table S14 Metrical parameters for $Zn_4O(O_2CAr)_6(dmso)_2$ (Ar = *p*-$C_6H_4NMe_2$) S63
Table S15 Metrical parameters for $Zn(O_2CAr)_2(dmf)_2$ (Ar = *p*-$C_6H_4NO_2$) S63
Table S16 Metrical parameters for (tmeda)$Zn(O_2CAr)_2$ compounds S64
Table S17 Metrical parameters for $(tpy^R)Zn(O_2CAr)_2$ (Ar = *p*-C_6H_4OMe) S64
Table S18 Metrical parameters for $[(phen)_2Zn(O_2CAr)]^+$ (Ar = *p*-C_6H_4OMe) S65
Table S19 Metrical parameters for (phen)$Zn(O_2CAr)_2(H_2O)$ (Ar = *p*-C_6H_4OMe) S65
Table S20 Metrical parameters for $[\{\kappa\text{-}N,O\text{-}NH_2C(CH_3)_2CH_2OH\}_2Zn(O_2CAr)][O_2CAr]$ S66
References S67

General Considerations. Unless specifically noted, all manipulations were performed open to atmospheric air on the benchtop. Any air- and moisture-sensitive manipulations are specifically noted and were performed using standard Schlenk techniques or in an inert atmosphere drybox with an atmosphere of purified nitrogen.

Chloroform-*d* and DMSO-d_6 were purchased from Sigma Aldrich or Cambridge Isotope Laboratories and used as received. Substituted benzoic acids, sodium bicarbonate, zinc sulfate heptahydrate, 1,10-phenanthroline, N,N,N',N'-tetramethylethylenediamine, methyl 4-(trifluoromethyl)benzoate, 2-amino-2-methyl-1-propanol, α,α,α,-trifluoroanisole, 4-(trifluoromethyl)benzaldehyde, N-bromosuccinimide, triethylamine and 4-(trifluoromethyl)benzoyl chloride were purchased from commercial suppliers and used as received. Anhydrous zinc acetate was purchased from Oakwood Chemical (CAS 557-34-6; item number 494138) and stored in a nitrogen-filled glovebox until use. General solvents were used without purification. The following was prepared by literature procedure: 4'-(4-methylphenyl)-2,2':6',2''-terpyridine.[1]

NMR spectra were recorded on a Magritek Spinsolve 60 Carbon spectrometer. ^{1}H and ^{13}C chemical shifts are reported in parts per million (ppm) relative to tetramethylsilane and referenced internally to the residual solvent peak. ^{19}F chemical shifts were referenced internally to a sealed capillary of α,α,α,-trifluoroanisole (-63.72 ppm vs $BF_3{\cdot}OEt_2$). Multiplicities are reported as follows: singlet (s), doublet (d), triplet (t), quartet (q), pentet (p). Infrared spectra were recorded using a PerkinElmer Spectrum 3 FT-IR spectrometer. Samples were either diluted into dry KBr and recorded as pellets or as neat solids by ATR.

Single crystals of $(dmf)_2Zn(O_2CAr)_2$ (Ar = *p*-$C_6H_4NO_2$), $Zn_4O(O_2CAr)_6$ (Ar = *p*-C_6H_4OMe), $(phen)Zn(H_2O)(O_2CAr)_2$ (Ar = *p*-C_6H_4OMe), $[(phen)_2Zn(O_2CAr)_2][X']$ (Ar = *p*-C_6H_4OMe), $(tmeda)Zn(O_2CAr)_2$ (Ar = *p*-$C_6H_4NO_2$), $(tmeda)Zn(O_2CAr)_2$ (Ar = *p*-C_6H_4OMe), and *N*-(2-hydroxy-1,1-dimethylethyl)-4-(trifluoromethyl)benzamide suitable for X-ray diffraction were coated with poly(isobutylene) oil and quickly transferred to the goniometer head of a Bruker AXS D8 Quest diffractometer with a fixed chi angle, a sealed tube fine-focus X-ray tube, single crystal curved graphite incident beam monochromator, and a Photon100 or a Photon2 CMOS area detector. Examination and data collection were performed with Mo Kα radiation (λ = 0.71073 Å). Single crystals of $Zn_4O(O_2CAr)_6(dmso)_2$ (Ar = *p*-$C_6H_4NMe_2$), $(tpy^R)Zn(O_2CAr)_2$ (Ar = *p*-C_6H_4OMe), and $[\{κ\text{-N,O-}NH_2C(CH_3)_2CH_2OH\}_2Zn(O_2CAr)][O_2CAr]$ (Ar = *p*-C_6H_4OMe) suitable for X-ray diffraction were coated with poly(isobutylene) oil and quickly transferred to the goniometer head of a Bruker AXS D8 Quest diffractometer with kappa geometry, an I-μ-S microsource X-ray tube, laterally graded multilayer (Goebel) mirror for monochromatization, a Photon2 or a Photon3 CMOS area detector, and an Oxford Cryosystems low temperature device. Examination and data collection were performed with Cu Kα radiation (λ = 1.54184 Å). Data were collected, reflections were indexed and processed, and the files scaled and corrected for absorption using APEX3, SAINT and SADABS.[2] For all samples, the space groups were assigned using XPREP within the SHELXTL suite of programs,[3] and the structures were solved by direct methods using ShelXS-97[4] and refined by full matrix least squares against F^2 with all reflections using Shelxl2018[5] using the graphical interface Shelxle.[6] If not specified otherwise, H atoms attached to carbon atoms were positioned geometrically and constrained to ride on their parent atoms, with carbon hydrogen bond distances of 0.95 Å for an aromatic C-H, 1.00, 0.99 and 0.98 Å for aliphatic C-H, CH_2, and CH_3 moieties, respectively. Methyl H atoms were allowed to rotate but not to tip to best fit the experimental electron density. $U_{iso}(H)$ values were set to a multiple of $U_{eq}(C)$ with 1.5 for CH_3, and 1.2 for CH_2, and C-H units, respectively. Additional data collection and refinement details, including description of disorder (where present) can be found with the individual structure descriptions below. Complete

crystallographic data, in CIF format, have been deposited with the Cambridge Crystallographic Data Centre. CCDC 2342373-2342381 and 2394928 contain the supplementary crystallographic data for this paper. These data can be obtained free of charge from The Cambridge Crystallographic Data Centre via www.ccdc.cam.ac.uk/data_request/cif.

General Synthesis: Reaction between $ZnSO_4$▪$7H_2O$, $NaHCO_3$, and $ArCO_2H$. A 250 mL Erlenmeyer flask was charged with a benzoic acid (34.779 mmol), a stir bar, and 50 mL of deionized water. While stirring, a solution of sodium bicarbonate (2.921 g, 34.770 mmol) in deionized water (15 mL) was added resulting in gas evolution. After 30 min, the mixture was filtered via vacuum filtration to remove insoluble particulates. The filtrate was slowly added to a stirring solution of $ZnSO_4$▪$7H_2O$ (5.000 g, 17.389 mmol) in deionized water (25 mL) resulting in the rapid precipitation of a white powder. The reaction was cooled to 0 °C and stirred for an additional 30 minutes. The solid was collected by vacuum filtration, washed with 100 mL deionized water, and dried in vacuo at room temperature until the mass of material no longer changed (8-24 hr).

$[Zn(O_2CAr)_2]_n$ Ar = C_6H_5. Yield: 4.065 g (13.215 mmol), 76%. ^{1}H NMR (dmso-d_6, 25 °C, 60 MHz): δ = 7.28-7.57 (m, 3H, *o*- and *p*-C*H*), 7.83-8.09 (m, 2H, *m*-C*H*). ^{13}C{^{1}H} NMR (dmso-d_6, 25 °C, 15 MHz): δ = 127.96 (*m*-*C*H), 129.58 (*o*-*C*H), 131.01 (*ipso*-*C*), 134.74 (*p*-*C*H), 171.77 (*C*=O). IR (ATR, neat): ν = 1634, 1595, 1576, 1527, 1494, 1407, 1071, 1027, 842, 713, 676 cm^{-1}.

$[Zn(O_2CAr)_2]_n$ Ar = *p*-$C_6H_4NO_2$. Yield: 6.214 g (15.629 mmol), 90%. ^{1}H NMR (dmso-d_6, 25 °C, 60 MHz): δ = 8.02-8.40 (m, 4H, *o*- and *m*-C*H*). ^{13}C{^{1}H} NMR (dmso-d_6, 25 °C, 15 MHz): δ = 123.34 (*m*-*C*H), 130.84 (*o*-*C*H), 141.10 (*ipso*-*C*), 149.10 (*p*-*C*), 169.53 (*C*=O). IR (ATR, neat): ν = 1544, 1348, 1317, 1107, 1012, 877, 843, 795, 721 cm^{-1}. Single, X-ray quality crystals of the *N*,*N*-dimethylformamide adduct were obtained by diffusing Et_2O into a DMF solution of $[Zn(O_2CAr)_2]_n$ (Ar = *p*-$C_6H_4NO_2$) at room temperature.

$[Zn(O_2CAr)_2]_n$ Ar = *p*-C_6H_4Br. Yield: 4.935 g (10.604 mmol), 61%. ^{1}H NMR (dmso-d_6, 25 °C, 60 MHz): δ = 7.58 (d, *J* = 8.4, 2H, *m*-C*H*), 7.88 (d, *J* = 8.4, 2H, *o*-C*H*). ^{13}C{^{1}H} NMR (dmso-d_6, 25 °C, 15 MHz): δ = 124.81 (*p*-*C*), 130.99 (*o*-*C*H), 131.65 (*m*-*C*H), 134.09 (*ipso*-*C*), 170.64 (*C*=O). IR (ATR, neat): ν = 1586, 1532, 1376, 1067, 1011, 864, 768 cm^{-1}.

$[Zn(O_2CAr)_2]_n$ Ar = *p*-$C_6H_4CH_3$. Yield: 4.266 g (12.709 mmol), 73%. ^{1}H NMR (dmso-d_6, 25 °C, 60 MHz): δ = 2.33 (s, 3H, C*H*$_3$), 7.19 (d, *J* = 7.9, 2H, *m*-C*H*), 7.86 (d, *J* = 7.9, 2H, *o*-C*H*). ^{13}C{^{1}H} NMR (dmso-d_6, 25 °C, 15 MHz): δ = 21.13 (*C*H$_3$), 128.60 (*m*-*C*H), 129.73 (*o*-*C*H), 132.01 (*ipso*-*C*), 140.83 (*p*-*C*), 171.99 (*C*=O). IR (ATR, neat): ν = 1590, 1543, 1507, 1379, 1172, 868, 849, 791, 762 cm^{-1}.

$[Zn(O_2CAr)_2]_n$ Ar = *p*-$C_6H_4OCH_3$. Yield: 2.995 g (8.037 mmol), 46%. ^{1}H NMR ($CDCl_3$, 25 °C, 60 MHz): δ = 3.84 (s, 3H, OC*H*$_3$), 6.87 (d, *J* = 8.8, 2H, *m*-C*H*), 8.14 (d, *J* = 8.8, 2H, *o*-C*H*). ^{13}C{^{1}H} NMR ($CDCl_3$, 25 °C, 15 MHz): δ = 55.52 (O*C*H$_3$), 113.40 (*m*-*C*H), 125.06 (*ipso*-*C*), 132.94 (*o*-*C*H), 163.46 (*p*-*C*), 175.05 (*C*=O). ^{1}H NMR (dmso-d_6, 25 °C, 60 MHz): δ = 3.78 (s, 3H, OC*H*$_3$), 6.93 (d, *J* = 8.7, 2H, *m*-C*H*), 7.92 (d, *J* = 8.7, 2H, *o*-C*H*). ^{13}C{^{1}H} NMR (dmso-d_6, 25 °C, 15 MHz): δ = 55.35 (O*C*H$_3$), 113.29 (*m*-*C*H), 127.00 (*ipso*-*C*), 131.66 (*o*-*C*H), 161.81 (*p*-*C*), 172.29 (*C*=O). IR (ATR, neat): ν = 1601, 1507, 1376, 1243, 1029, 781 cm^{-1}. Single, X-ray quality crystals of the tetranuclear cluster, $Zn_4O(O_2CAr)_6$ (Ar = *p*-$C_6H_4OCH_3$), were obtained by the diffusion of hexane into a toluene solution of $[Zn(O_2CAr)_2]_n$ (Ar = *p*-$C_6H_4OCH_3$) containing trace 1,2-dichloroethane at room temperature.

[Zn(O$_2$CAr)$_2$]$_n$ Ar = *p*-C$_6$H$_4$NMe$_2$. Yield: 2.845 g (7.226 mmol), 42%. ^{1}H NMR (dmso-*d*$_6$, 25 °C, 60 MHz): δ = 2.96 (s, 6H, N(C*H*$_3$)$_2$), 6.66 (d, *J* = 8.2, 2H, *m*-C*H*), 7.83 (d, *J* = 8.8, 2H, *o*-C*H*). The low solubility of [Zn(O$_2$CAr)$_2$]$_n$ (Ar = *p*-C$_6$H$_4$NMe$_2$) precluded meaningful ^{13}C{^{1}H} NMR analysis. Single, X-ray quality crystals of the solvated tetranuclear cluster, Zn$_4$O(O$_2$CAr)$_6$(dmso)$_2$ (Ar = *p*-C$_6$H$_4$NMe$_2$), were obtained by the diffusion of Et$_2$O into a dmso solution of [Zn(O$_2$CAr)$_2$]$_n$ (Ar = *p*-C$_6$H$_4$NMe$_2$) at room temperature.

[Zn(O$_2$CAr)$_2$]$_n$ Ar = *p*-C$_6$H$_4$CF$_3$. Yield: 6.533 g (14.727 mmol), 85%. ^{1}H NMR (dmso-*d*$_6$, 25 °C, 60 MHz): δ = 7.76 (d, *J* = 8.3, 2H, *m*-C*H*), 8.14 (d, *J* = 8.3, 2H, *o*-C*H*). ^{13}C{^{1}H} NMR (dmso-*d*$_6$, 25 °C, 15 MHz): δ = 124.25 (q, *J* = 272, *C*F$_3$), 125.05 (q, *J* = 3.8, *m*-Ar-*C*H), 130.27 (*o*-Ar-*C*H), 130.97 (q, *J* = 31.5, *p*-Ar-*C*-CF$_3$), 138.92 (*ipso*-Ar-*C*), 170.14 (*C*=O). IR (ATR, neat): ν = 1596, 1547, 1512, 1410, 1315, 1182, 1163, 1127, 1104, 1063, 1017, 866, 789, 775, 713 cm^{-1}.

[Zn(O$_2$CAr)$_2$]$_n$ Ar = *p*-C$_6$H$_4$CH$_2$Cl. Yield: 6.124 g (15.138 mmol), 87%. ^{1}H NMR (dmso-*d*$_6$, 25 °C, 60 MHz): δ = 4.80 (s, 2H, C*H*$_2$Cl), 7.46 (d, *J* = 7.0, 2H, *m*-C*H*), 7.95 (d, *J* = 7.0, 2H, *o*-C*H*). ^{13}C{^{1}H} NMR (dmso-*d*$_6$, 25 °C, 15 MHz): δ = 45.71 (*C*H$_2$Cl), 128.41 (Ar-*C*H), 129.83 (Ar-*C*H), 134.67 (Ar-*C*), 140.36 (Ar-*C*), 170.94 (*C*=O). IR (ATR, neat): ν = 1588, 1521, 1425, 1270, 860, 787, 718, 682 cm^{-1}.

Synthesis of [Zn(O$_2$C^{t}Bu)$_2$]$_n$. A 100 mL Erlenmeyer flask was charged with pivalic acid (3.552 g, 34.779 mmol), 20 mL deionized H$_2$O, and a stir bar. In a separate flask, NaHCO$_3$ (2.921 g, 34.770 mmol) was dissolved in 20 mL deionized H$_2$O. The solution of NaHCO$_3$ was slowly added to the pivalic acid solution resulting in CO$_2$ release as evidenced by effervescence. After 20 min, the solution was added to a separate flask containing ZnSO$_4$·7H$_2$O dissolved in deionized H$_2$O (20 mL) resulting in rapid precipitation of a white solid. The flask was chilled to 0 °C and stirred an additional 20 min. The solid was collected via vacuum filtration, washed with deionized H$_2$O (3 x 20 mL). The solid was dried in vacuo at room temperature to afford white microcrystalline material (1.928 g, 7.204 mmol, 41%) identified as [Zn(O$_2$C^{t}Bu)$_2$]$_n$. The IR spectrum (ATR) obtained was compared to literature.[16] IR (ATR, neat): 2963, 1605, 1534, 1482, 1457, 1425, 1379, 1361, 1228, 1031, 938, 899, 792 cm^{-1}.

Attempted Synthesis of [Zn(ip)$_2$]$_n$ (ip = C$_8$H$_4$O$_4$; isophthalic acid).[21] A 250 mL Erlenmeyer flask was charged with isophthalic acid, 50 mL deionized H$_2$O, and a stir bar. In a separate flask, NaHCO$_3$ (2.921 g, 34.770 mmol) was dissolved in 15 mL deionized H$_2$O. The solution of NaHCO$_3$ was slowly added to the phthalic acid solution resulting in CO$_2$ release as evidenced by effervescence. After 20 min, the solution was filtered to remove minimal amounts of insoluble particulates. The solution was then added to a separate flask containing ZnSO$_4$·7H$_2$O (5.000 g, 17.389 mmol), dissolved in deionized H$_2$O (25 mL). No precipitate formed. After 5 min, the flask was chilled to 0 °C and stirred an additional 30 min. No precipitate was observed, contrasting the results observed in the general procedure above. After storing 18 hr at RT, a minimal amount of white, non-crystalline powder precipitate (0.238 g). This material was investigated by infrared spectroscopy and did not match the literature report.[21]

Synthesis of (tpy^R)Zn(O_2CAr)$_2$ (Ar = *p*-C_6H_4OMe). A 20 mL scintillation vial was charged with [Zn(O_2CAr)$_2$]$_n$ (Ar = *p*-C_6H_4OMe; 0.107 g, 0.291 mmol) and 6 mL of MeOH. A separate 20 mL scintillation vial containing a suspension of 4'-(4-methylphenyl)-2,2':6',2''-terpyridine (tpy^R; 0.094 g, 0.291 mmol) in 2 mL of MeOH was added to the zinc-containing vial. After 4 hr, the solution was filtered over Celite and dried. The resulting material was washed with 20 mL *n*-hexane and dried to afford off-white powder. The bulk material was recrystallized by layering a dichloromethane solution with hexanes to afford a light-yellow crystalline material (0.098 g, 0.142 mmol, 49%) assigned as (tpy^R)Zn(O_2CAr)$_2$ (Ar = *p*-C_6H_4OMe). The recrystallized material has minimal solubility on common NMR solvents including $CDCl_3$ and dmso-d_6, precluding $^{13}C\{^1H\}$ NMR analysis. Single, X-ray quality crystals were obtained by slow diffusion of hexanes into a dichloromethane solution of (tpy^R)Zn(O_2CAr)$_2$ (Ar = *p*-C_6H_4OMe) at room temperature. ^1H NMR ($CDCl_3$, 25 °C, 60 MHz): δ = 2.42 (s, 3H, C*H*$_3$), 3.79 (s, 6H, OC*H*$_3$), 6.80 (d, *J* = 8.8, 4H, *m*-benzoate-C*H*), 7.25 (m, 2H, Ar-C*H*), 7.59 (t, *J* = 6.9, 2H, Ar-C*H*), 7.79 (d, *J* = 7.0, 2H, Ar-C*H*), 8.03 (d, *J* = 8.9, *o*-benzoate-C*H*), 8.04 (m, 2H, Ar-C*H*), 8.24 (m, 2H, Ar-C*H*), 8.31 (s, 2H, pyridine-C*H*), 8.99 (d, *J* = 4.8, 2H, *o*-pyridine-C*H*). IR (ATR, neat): 1600, 1552, 1507, 1477, 1359, 1308, 1246, 1163, 1097, 1015, 851, 825, 783, 730, 699, 659 cm^{-1}.

Synthesis of (phen)$_2$Zn(O_2CAr)$_2$ (Ar = *p*-C_6H_4OMe). A 20 mL scintillation vial was charged with [Zn(O_2CAr)$_2$]$_n$ (Ar = *p*-C_6H_4OMe; 0.100 g, 0.272 mmol) and 6 mL of MeOH. A separate 20 mL scintillation vial containing a solution of 1,10-phenanthroline (phen; 0.092 g, 0.511 mmol) in 2 mL of MeOH was added to the zinc-containing vial with stirring. After 4 hr, the solution was filtered over Celite to remove a fine white precipitate. Volatiles were removed in vacuo and the resulting material was washed with 20 mL *n*-hexane and dried to afford white powder (0.167 g, 0.229 mmol, 91%) assigned as (phen)$_2$Zn(O_2CAr)$_2$ (Ar = *p*-C_6H_4OMe). Single, X-ray quality crystals of two different species were obtained by diffusing hexanes into a 1,2-dichloromethane solution of (phen)$_2$Zn(O_2CAr)$_2$ at room temperature: (phen)Zn(H_2O)(O_2CAr)$_2$ and [(phen)$_2$Zn(O_2CAr)$_2$][X']. ^1H NMR ($CDCl_3$, 25 °C, 60 MHz): δ = 3.67 (s, 6H, OC*H*$_3$), 6.72 (d, *J* = 8.7, 4H, *m*-Ar-C*H*), 7.56 (t, *J* = 4.6, 4H, phen-3,8-C*H*), 7.66 (s, 4H, phen-5,6-C*H*), 8.02 (d, *J* = 8.7, 4H, *o*-Ar-C*H*), 8.22 (d, *J* = 8.2, 4H, phen-4,7-C*H*), 9.19 (d, *J* = 4.6, 4H, phen-2,9-C*H*). $^{13}C\{^1H\}$ NMR ($CDCl_3$, 25 °C, 15 MHz): δ = 55.13 (O*C*H$_3$), 112.81 (*m*-Ar-*C*H), 124.55 (phen-3,8-*C*H), 126.67 (phen-5,6-*C*H), 128.64 (*ipso*-Ar-*C*), 131.98 (*o*-Ar-*C*H), 138.22 (phen-4,7-*C*H and phen-quaternary-*C*), 142.47 (phen-5,6-*C*H), 149.82 (phen-2,9-*C*H), 161.89 (*p*-Ar-*C*), 175.40 (*C*=O). IR (ATR, neat): 1593, 1548, 1516, 1367, 1245, 1168, 1103, 1027, 846, 785, 724 cm^{-1}.

Synthesis of (tmeda)Zn(O_2CAr)$_2$ (Ar = *p*-C_6H_4OMe). A 20 mL scintillation vial was charged with [Zn(O_2CAr)$_2$]$_n$ (Ar = *p*-C_6H_4OMe; 0.100 g, 0.272 mmol) and 6 mL of MeOH. A separate 20 mL scintillation vial containing a solution of N,N,N',N'-tetramethylethylenediamine (tmeda; 0.038 mL, 0.253 mmol) in 2 mL of MeOH was added to the zinc-containing vial while stirring. After 4 hr, the solution was filtered over Celite to remove a fine white precipitate. Volatiles were removed in vacuo and the resulting material was washed with 20 mL *n*-hexane and dried to afford white powder (0.104 g, 0.229 mmol, 91%) assigned as (tmeda)Zn(O_2CAr)$_2$ (Ar = *p*-C_6H_4OMe). Single, X-ray quality crystals were obtained by diffusing hexanes into a toluene solution of (tmeda)Zn(O_2CAr)$_2$ (Ar = *p*-C_6H_4OMe) at room temperature. ^1H NMR ($CDCl_3$, 25 °C, 60 MHz): δ = 2.62 (s, 12H, N-C*H*$_3$), 2.77 (s, 4H, N-C*H*$_2$), 3.82 (s, 6H, OC*H*$_3$), 6.85 (d, *J* = 8.8, 4H, *m*-Ar-C*H*), 8.07 (d, *J* = 8.8, 4H, *o*-Ar-C*H*). $^{13}C\{^1H\}$ NMR ($CDCl_3$, 25 °C, 15 MHz): δ = 46.80 (N-*C*H$_3$), 55.42 (OC*H*$_3$ or N-*C*H$_2$), 56.83 (OC*H*$_3$ or N-*C*H$_2$), 113.06 (*m*-Ar-*C*H), 126.48 (*ipso*-Ar-*C*), 132.27 (*o*-Ar-*C*H). The remaining benzoate resonances are not clearly resolved. IR (ATR, neat): 1595, 1543, 1398, 1310, 1251, 1170, 1103, 1027, 956, 857, 785, 703 cm^{-1}.

Synthesis of (tmeda)Zn(O_2CAr)$_2$ (Ar = *p*-$C_6H_4NO_2$). A 125 mL Erlenmeyer flask was charged with [Zn(O_2CAr)$_2$]$_n$ (Ar = *p*-$C_6H_4NO_2$; 0.300 g, 0.755 mmol) and 30 mL of acetone and stirred. Insoluble particulates (minimal) were removed via vacuum filtration over Celite; then, N,N,N',N'-tetramethylethylenediamine (tmeda; 0.113 mL, 0.757 mmol) in 2 mL of acetone was added dropwise to the stirring zinc solution. A fine white precipitate immediately formed. After 5 minutes, an equal volume of hexanes was added to induce further precipitation and the mixture was stored at 0 °C overnight. The solid was collected and dried in vacuo to afford white powder assigned as (tmeda)Zn(O_2CAr)$_2$ (Ar = *p*-$C_6H_4NO_2$). Single, X-ray quality crystals were obtained by diffusing diethyl ether into a 1,2-dichloromethane solution of (tmeda)Zn(O_2CAr)$_2$ (Ar = *p*-$C_6H_4NO_2$) at room temperature. The material has minimal solubility in $CDCl_3$ and dmso-d$_6$, precluding analysis by NMR spectroscopy. IR (ATR): 1618, 1568, 1517, 1468, 1414, 1341, 1287, 1102, 1023, 1010, 950, 890, 838, 799, 767, 727 cm^{-1}.

General catalytic procedure for synthesis of 4,4'-dimethyl-2-(4-trifluoromethyl)phenyl-4,5-dihydrooxazole. All catalytic trials were set up open to air. A 20 mL scintillation vial was charged with a catalyst (0.060 mmol; 10 mol%) and a stir bar. Via volumetric pipets, methyl 4-(trifluoromethyl)benzoate (0.500 mL of a 1.196 M stock solution in C_6H_5Cl; 0.598 mmol) and 2-amino-2-methyl-1-propanol (0.500 mL of a 1.439 M stock solution in C_6H_5Cl; 0.720 mmol; 1.2 equiv) were added. The vials were sealed with a Teflon-lined cap and stirred in an aluminum heating block at 100 °C for 18 hrs. The samples were cooled to RT and α,α,α,-trifluoroanisole (0.500 mL of a 1.196 M stock solution in C_6H_5Cl, 0.598 mmol) was added via volumetric pipet as an internal standard. The solution was filtered to remove insoluble colorless/white particulates and analyzed by ^{19}F NMR. A minimum of four trials were performed for each catalyst.

Crystal growth for *N*-(2-hydroxy-1,1-dimethylethyl)-4-(trifluoromethyl)benzamide. The general catalytic procedure was employed with catalyst [Zn(O_2CAr)$_2$]$_n$ (Ar = *p*-$C_6H_4CF_3$). At the conclusion of the reaction, the vial was allowed to cool to RT and filtered. The solution was left undisturbed at room temperature for 15 min during which time single, X-ray quality crystals of *N*-(2-hydroxy-1,1-dimethylethyl)-4-(trifluoromethyl)benzamide formed. While these crystals were analyzed, the same crystal growth, qualitatively, was observed, regardless of the [Zn(O_2CAr)$_2$]$_n$ catalyst used. From a separate set of crystals, the mother liquor was decanted, the crystals washed with hexanes, and the material analyzed by ^{1}H NMR spectroscopy (dmso-*d*$_6$). The ^{1}H NMR analysis is consistent with *N*-(2-hydroxy-1,1-dimethylethyl)-4-(trifluoromethyl)benzamide and was compared to an authentic sample (see below).

Catalytic procedure for synthesis of 4,4'-dimethyl-2-(4-trifluoromethyl)phenyl-4,5-dihydrooxazole from *N*-(2-hydroxy-1,1-dimethylethyl)-4-(trifluoromethyl)benzamide. All catalytic trials were set up open to air. A 20 mL scintillation vial was charged with catalyst [Zn(O_2CAr)$_2$]$_n$ (Ar = *p*-C_6H_4OMe) (0.022 g, 0.060 mmol; 10 mol%), *N*-(2-hydroxy-1,1-dimethylethyl)-4-(trifluoromethyl)benzamide (0.156 g, 0.597 mmol), and a stir bar. Via volumetric pipet, 1.000 mL of C_6H_5Cl was added. The vial was sealed with a Teflon-lined cap and stirred in an aluminum heating block at 100 °C for 18 hrs. The samples were cooled to RT and α,α,α,-trifluoroanisole (0.500 mL of a 1.196 M stock solution in C_6H_5Cl, 0.598 mmol) was added via volumetric pipet as an internal standard. The solution was filtered to remove insoluble, colorless/white particulates and analyzed by ^{19}F NMR. Six trials were performed. Average yield of 4,4'-dimethyl-2-(4-trifluoromethyl)phenyl-4,5-dihydrooxazole: 44% +/- 6%.

Independent synthesis of 4,4'-dimethyl-2-(4-trifluoromethyl)phenyl-4,5-dihydrooxazole. This molecule was prepared according to modification of literature protocol.[7] An oven-dried 250 mL three-neck round bottom flask was charged with activated molecular sieves (4 Å, 20 g), dichloromethane (80 mL), 4-(trifluoromethyl)benzaldehyde (3.480 g, 19.986 mmol), and 2-amino-2-methylpropan-1-ol (2.680 g, 30.065 mmol) against a flow of N_2. The reaction mixture was gently stirred (~200 rpm) for 20 hr at room temperature; then, N-bromosuccinimide (5.34 g, 30.002 mmol) was added against a flow of N_2. The resulting mixture was stirred for 4 hr, then filtered. The organic solution was washed with $NaHCO_3$ (aq. sat.; 3 x 25 mL) and $Na_2S_2O_3$ (aq. sat.; 1 x 50 mL), then dried over $MgSO_4$. Volatiles were removed to afford a light-yellow oil. The crude material was purified by flash column chromatography on SiO_2 to afford colorless solid (1.935 g, 7.955 mmol, 40%) assigned as 4,4'-dimethyl-2-(4-trifluoromethyl)phenyl-4,5-dihydrooxazole. R_f = 0.69 (3:1 hexane:EtOAc; impurity R_f = 0.79). R_f = 0.59 (4:1 hexane:EtOAc; impurity R_f = 0.73). ^{1}H NMR ($CDCl_3$, 25 °C, 60 MHz): δ = 1.38 (s, 6H, C(C*H*$_3$)$_2$), 4.13 (s, 2H, C*H*$_2$), 7.63 (d, *J* = 8.2, 2H, Ar-C*H*), 8.06 (d, *J* = 8.2, 2H, Ar-C*H*). ^{13}C{^{1}H} NMR ($CDCl_3$, 25 °C, 15 MHz): δ = 28.47 (*C*H$_3$), 68.06 (*C*(CH$_3$)$_2$), 79.49 (*C*-O), 123.97 (q, *J* = 268.2, *C*F$_3$), 125.36 (q, *J* = 3.8, *o*-to-CF$_3$ *C*H), 128.73 (*m*-to-CF$_3$ C-H), 131.70 (*C*-C=O), 131.85 (q, *J* = 64.4, *C*-CF$_3$), 161.02 (*C*=N). ^{19}F NMR ($CDCl_3$, 25 °C, 56 MHz): δ = -63.29 (s, C*F*$_3$). IR (ATR, neat): ν = 2971, 1651, 1619, 1581, 1464, 1411, 1322, 1167, 1106, 1069, 1016, 963, 921, 852, 755, 686 cm^{-1}. MP: 309-310 K.

Independent synthesis of *N*-(2-hydroxy-1,1-dimethylethyl)-4-(trifluoromethyl)benzamide. This molecule was prepared according to modification of literature protocol.[8] A 100 mL RBF was charged with 2-amino-2-methylpropanol (2.137 g, 23.965 mmol), dichloromethane (40 mL), triethylamine (6.688 mL, 47.951 mmol), and a stir bar. The solution was cooled to 0 °C and sparged with N_2. While stirring, 4-(trifluoromethyl)benzoyl chloride (5.000 g, 23.974 mmol) was added over the course of 2 min. The reaction was stirred for 2 hr while gradually warming to RT as the ice bath warmed. The solution was transferred to a separatory funnel with 20 mL DI-H_2O and 20 mL sat. aq. $NaHCO_3$. The organics were extracted with dichloromethane (2 x 20 mL), washed with 20 mL brine, and dried over $MgSO_4$. Volatiles were removed by rotary evaporation to afford white solid. The solid was collected on a Buchner funnel, washed with 20 mL of hexanes, and dried to afford white powder (4.240 g, 16.230 mmol, 68%) identified as *N*-(2-hydroxy-1,1-dimethylethyl)-4-(trifluoromethyl)benzamide. ^{1}H NMR ($CDCl_3$, 25 °C, 60 MHz): δ = 1.42 (s, 6H, C(C*H*$_3$)$_2$), 3.68 (s, 2H, C*H*$_2$), 4.30 (broad, 1H, N*H* or O*H*), 6.38 (broad, 1H, N*H* or O*H*), 7.64 (d, *J* = 8.7, 2H, Ar-C*H*), 7.85 (d, *J* = 8.7, 2H, Ar-C*H*). ^{1}H NMR (dmso-*d*$_6$, 25 °C, 60 MHz): δ = 1.33 (s, 6H, C(C*H*$_3$)$_2$), 3.53 (s, 2H, C*H*$_2$), 4.95 (broad, 1H, N*H* or O*H*), 7.76 (d, *J* = 8.2, 2H, Ar-C*H*), 8.02 (d, *J* = 8.2, 2H, Ar-C*H*). ^{13}C{^{1}H} NMR (dmso-*d*$_6$, 25 °C, 15 MHz): δ = 23.45 (*C*H$_3$), 55.27 (*C*(CH$_3$)$_2$), 55.27 (*C*-O), 124.01 (q, *J* = 268, *C*F$_3$), 124.98 (q, *J* = 3.8, *o*-to-CF$_3$ *C*-H), 128.27 (*m*-to-CF$_3$ *C*-H), 130.84 (q, *J* = 31.3, *C*-CF$_3$), 139.61 (*C*-C=O), 165.41 (*C*=O). IR (ATR, neat): ν = 3267 (OH), 3098, 2878, 1725, 1631, 1561, 1459, 1387, 1363, 1321, 1278, 1163, 1110, 1056, 1015, 879, 855, 767, 695, 683 cm^{-1}.

Control reactions between $[Zn(O_2CAr)_2]_n$ and individual substrates or substrate surrogates. All stock solutions were prepared in C_6H_5Cl. A 20 mL scintillation vial was charged with $[Zn(O_2CAr)_2]_n$ (Ar = *p*-C_6H_4OMe; 0.022 g, 0.060 mmol) and a stir bar. Via volumetric pipet, 0.5 mL of the stock solutions of the substrates of interest were added (ester: 1.196 M, 10 equiv.; amino alcohol: 1.439 M, 12 equiv.; *tert*-butylamine: 1.439 M, 12 equiv; *n*-propyl alcohol: 1.439 M, 12 equiv.). For reactions with a fluorine-containing substrate, 0.5 mL of a $PhOCF_3$ stock solution were added (1.196 M; 10 equiv.) via volumetric pipet. Every reaction was performed with a total of 2.0 mL of solution. The total volume of was achieved via addition of the appropriate amount of a C_6H_5Cl solution containing tetramethylsilane (0.598 M). Reactions were stirred for 30 min at either RT (~18 °C) or 100 °C. Once reactions cooled to room temperature, each was filtered and characterized by NMR spectroscopy.

Crystal growth for $[\{\kappa\text{-}N,O\text{-}NH_2C(CH_3)_2CH_2OH\}_2Zn(O_2CAr)][O_2CAr]$ (Ar = *p*-C_6H_4OMe). The control reaction was performed, as described above, between the catalyst $[Zn(O_2CAr)_2]_n$ (Ar = *p*-C_6H_4OMe; 0.022 g, 0.060 mmol) and the amino alcohol, 2-amino-2-methyl-1-propanol (0.5 mL, 1.439 M, 12 equiv.) in 2.0 mL total C_6H_5Cl solution with tetramethylsilane as an internal standard. The reaction was heated to 100 °C for 30 min and allowed to cool to RT. The solution was filtered and assessed by ^1H NMR. The remaining solution was left undisturbed in a 20 mL scintillation vial for 3 days at RT (~18 °C) resulting in the formation of single, colorless crystals. This protocol was performed for seven separate trials and produced crystalline material in each instance.

Table S1. Literature comparison of crystallographically characterized $(L)_2Zn(O_2CAr)_2$ and $Zn_4O(O_2CAr)_6$ complexes as a function of their Hammett parameter.

Ar = 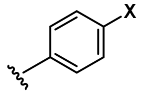		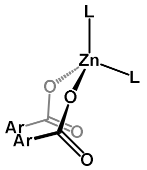	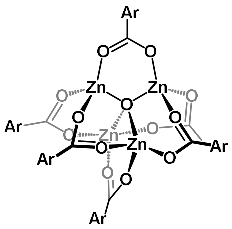
	σ_p	CCDC Code	CCDC Code
X = NO_2	+0.78	CEHCAT01[9] / This work	
X = CF_3	+0.43		
X = CHO	+0.35	KIWMEJ[10] / SESWET[11]	
X = Cl	+0.33	HACZIU[12]	
X = Br	+0.23	KIWNOU[13]	
X = CH_2Cl	+0.11		
X = C_6H_5	+0.06		KEFFAE[14]
X = H	0.00	ZZZRHA[15]	VOLTEV[16] / XADJOC02[17]
X = SMe	0.00		KEFDUW[14]
X = CH_3	-0.17	MIRSEM[18] / 01[19] / 02[20]	KEFDOQ[14]
X = OCH_3	-0.27		This work
X = NMe_2	-0.83		This work

Scheme S1. Literature reported protocol for the generation of various forms of the parent zinc benzoate.

1) *New J. Chem.* 2013, *37*, 1529-1537
$Zn(NO_3)_2{\cdot}6H_2O + 2\ NaO_2CPh \xrightarrow{H_2O} [Zn(O_2CPh)_2]_n$

2) *Spectrochim. Acta A Mol. Biomol. Spectrosc.* 2012, *97*, 1002-1006
$ZnCl_2$ (1M, aq.) + HO_2CPh (1M, EtOH) $\xrightarrow[\text{pH = 5-6 (HOAc)}]{Na_2SiO_3\ /\ H_2O}$ $[Zn(O_2CPh)_2]_n$

3) *Inorg. Chim. Acta* 1991, *186*, 51-60
$[Zn(O_2CPh)_2]_n \xrightarrow[\text{acetone, warming}]{MgSO_4} Zn_4O(O_2CPh)_6$

4) *J. Am. Chem. Soc.* 1948, *70*, 2151-2154
$ZnCl_2 + NaO_2CPh + HO_2CPh \xrightarrow{H_2O} [Zn(O_2CPh)_2(H_2O)_2{\cdot}2HO_2CPh$

5) *J. Am. Chem. Soc.* 1948, *70*, 2151-2154
$ZnCO_3 + HO_2CPh \xrightarrow{H_2O} [Zn(O_2CPh)_2(H_2O)_2{\cdot}2HO_2CPh$

6) *J. Am. Chem. Soc.* 1948, *70*, 2151-2154
"zinc borate" + $HO_2CPh \xrightarrow{H_2O,\ hot} [Zn(O_2CPh)_2(H_2O)_2{\cdot}2HO_2CPh$

7) *Acta Cryst.* 2005, *E61*, m1340-m1342
$Zn(NO_3)_2{\cdot}6H_2O + 2\ NaO_2CPh \xrightarrow[\text{473 K, 100 hr}]{H_2O} [Zn_2(O_2CPh)_3(OH)]_n$

8) *Acta Cryst.* 2005, *E61*, m1340-m1342
$Zn_4O(O_2CPh)_6 + 1\ H_2O \xrightarrow{THF} Zn_4O(O_2CPh)_6(H_2O)(THF)$

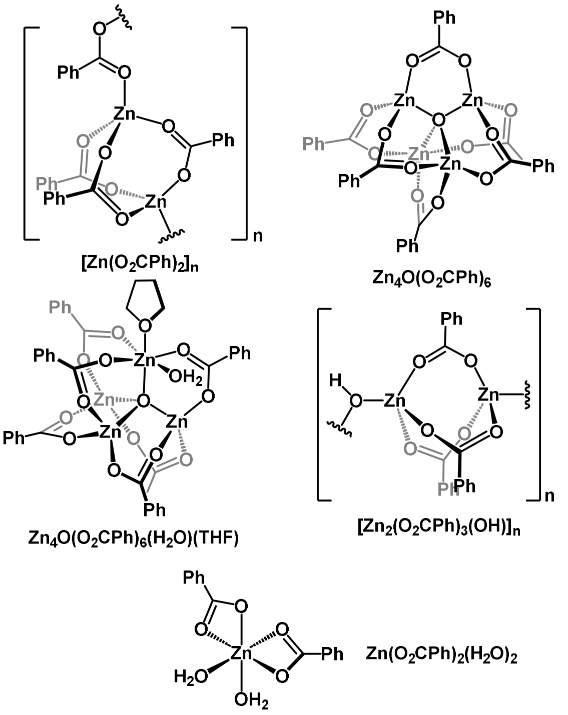

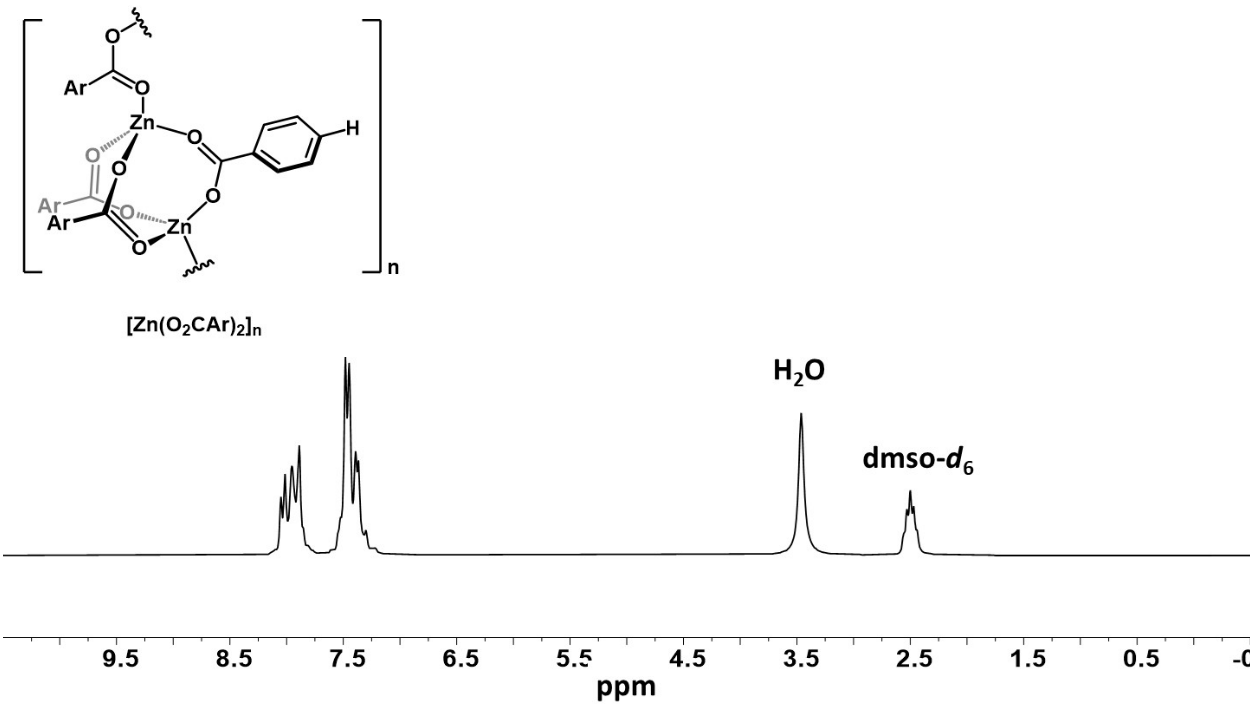

Figure S1. ^{1}H NMR spectrum (dmso-*d*$_6$, 25 °C, 60 MHz) of $[Zn(O_2CAr)_2]_n$ (Ar = C_6H_5).

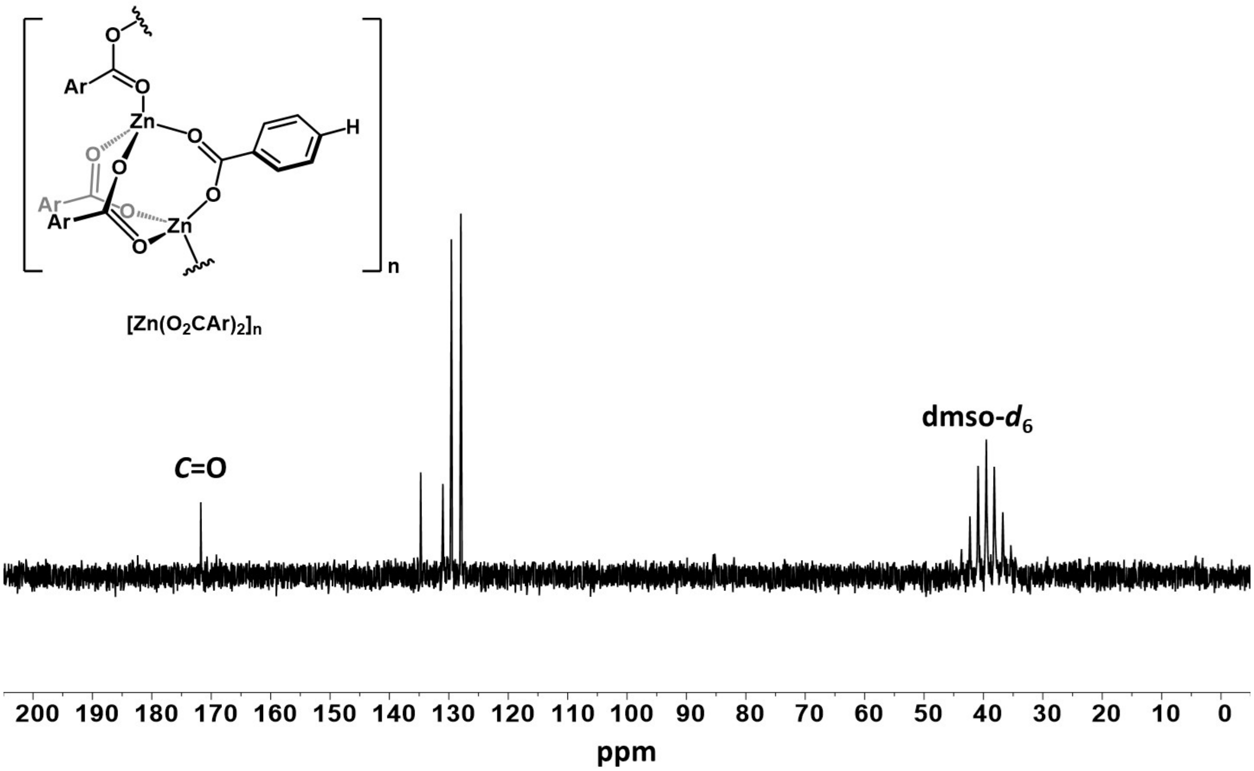

Figure S2. ^{13}C{^{1}H} NMR spectrum (dmso-*d*$_6$, 25 °C, 15 MHz) of $[Zn(O_2CAr)_2]_n$ (Ar = C_6H_5).

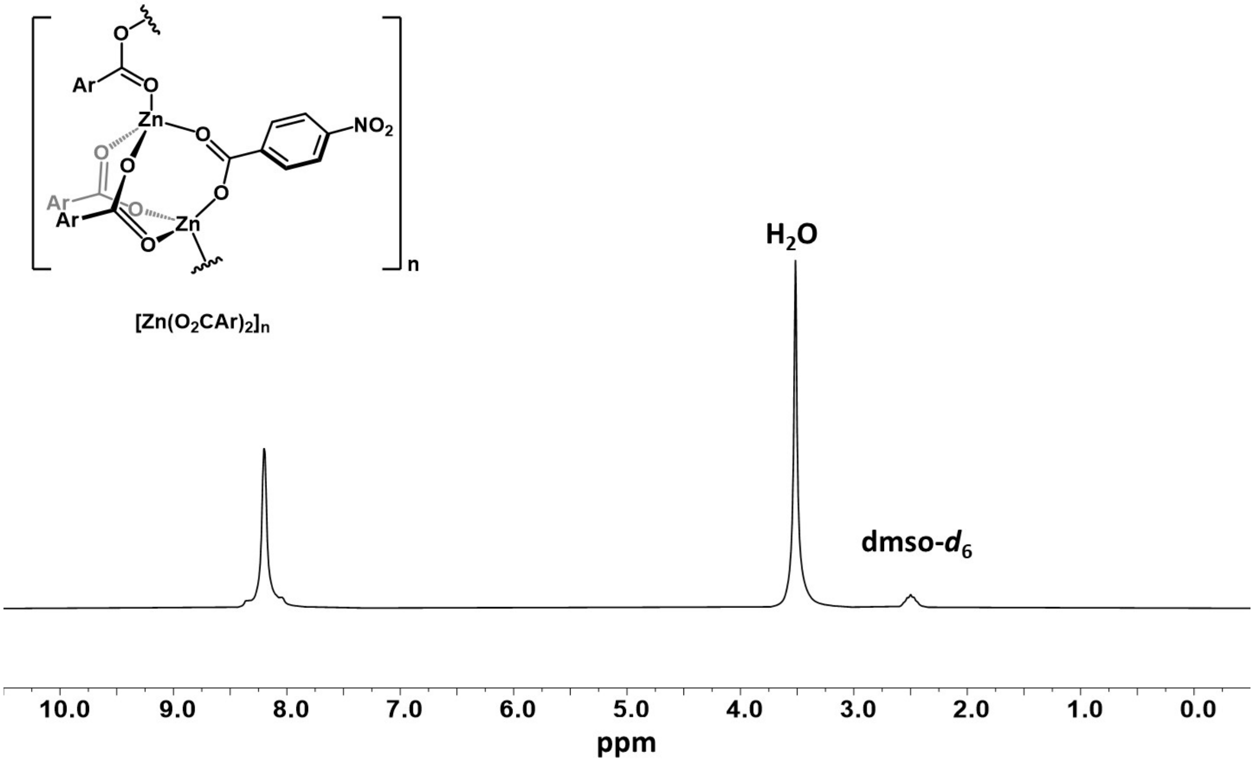

Figure S3. ^{1}H NMR spectrum (dmso-d_6, 25 °C, 60 MHz) of $[Zn(O_2CAr)_2]_n$ (Ar = p-$C_6H_4NO_2$).

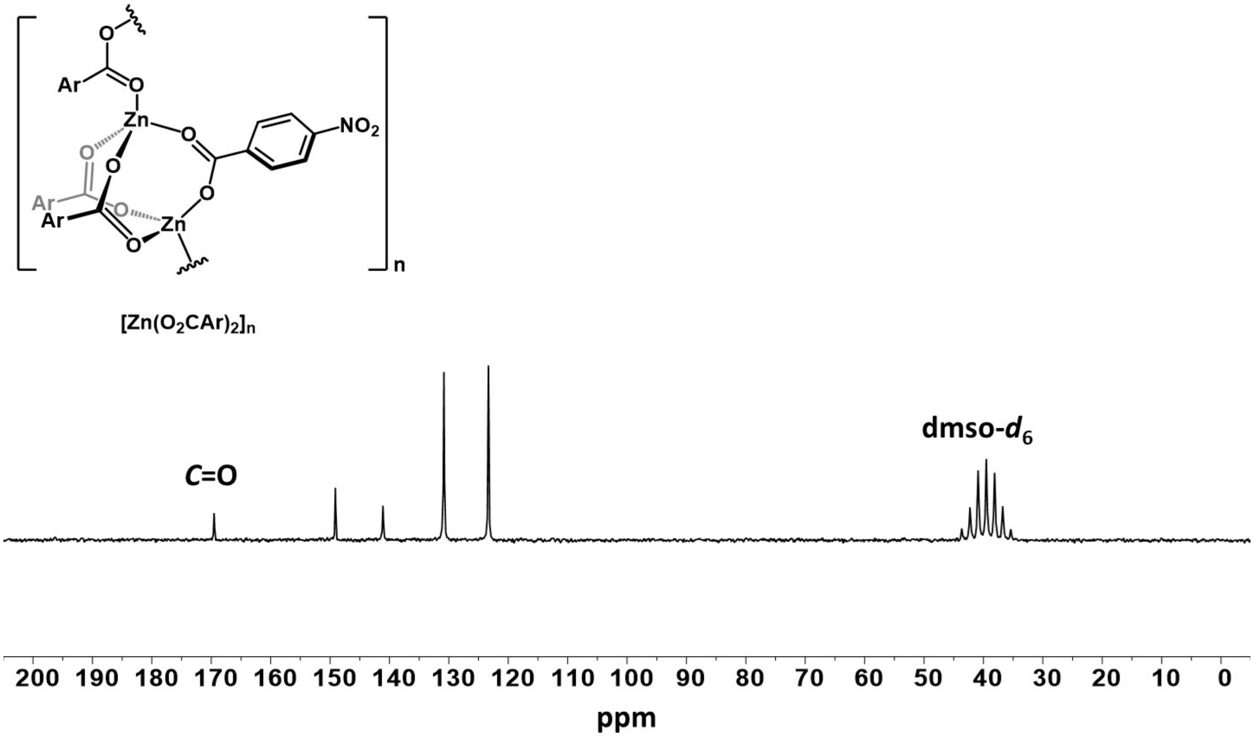

Figure S4. $^{13}C\{^{1}H\}$ NMR spectrum (dmso-d_6, 25 °C, 15 MHz) of $[Zn(O_2CAr)_2]_n$ (Ar = p-$C_6H_4NO_2$).

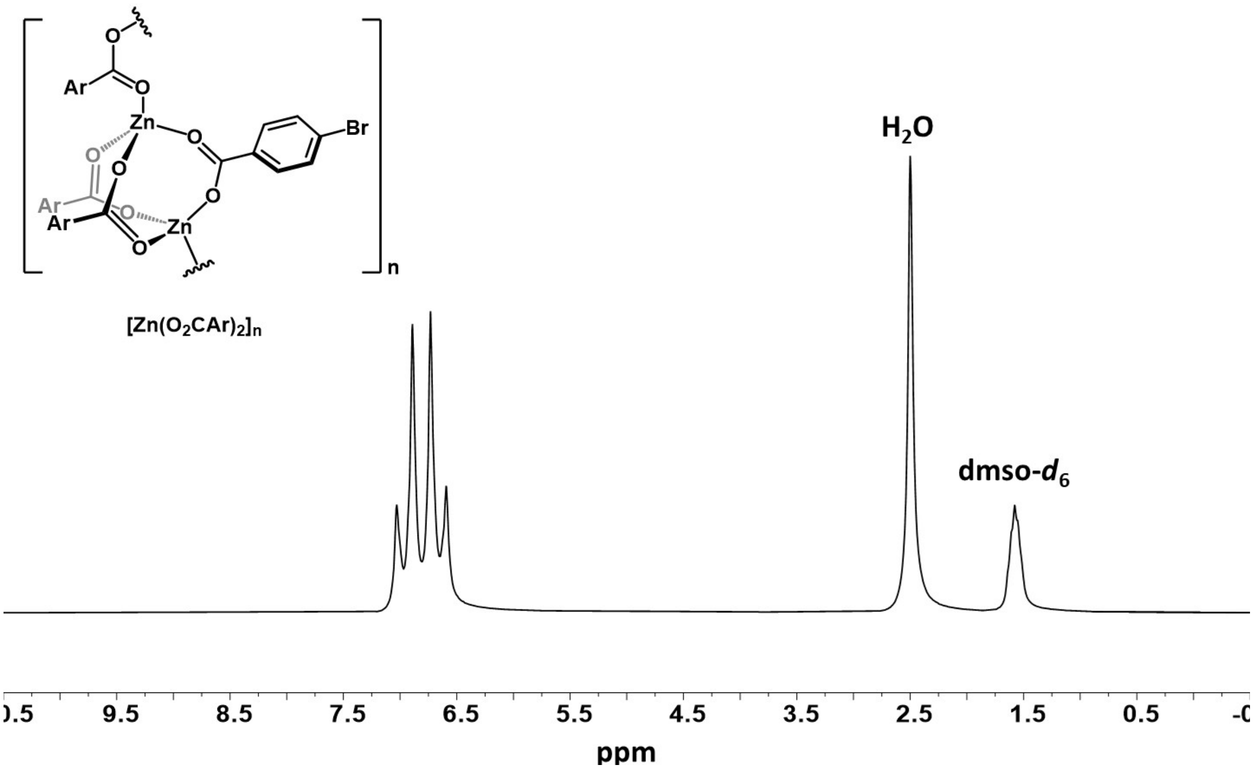

Figure S5. ^{1}H NMR spectrum (dmso-d_6, 25 °C, 60 MHz) of $[Zn(O_2CAr)_2]_n$ (Ar = *p*-C_6H_4Br).

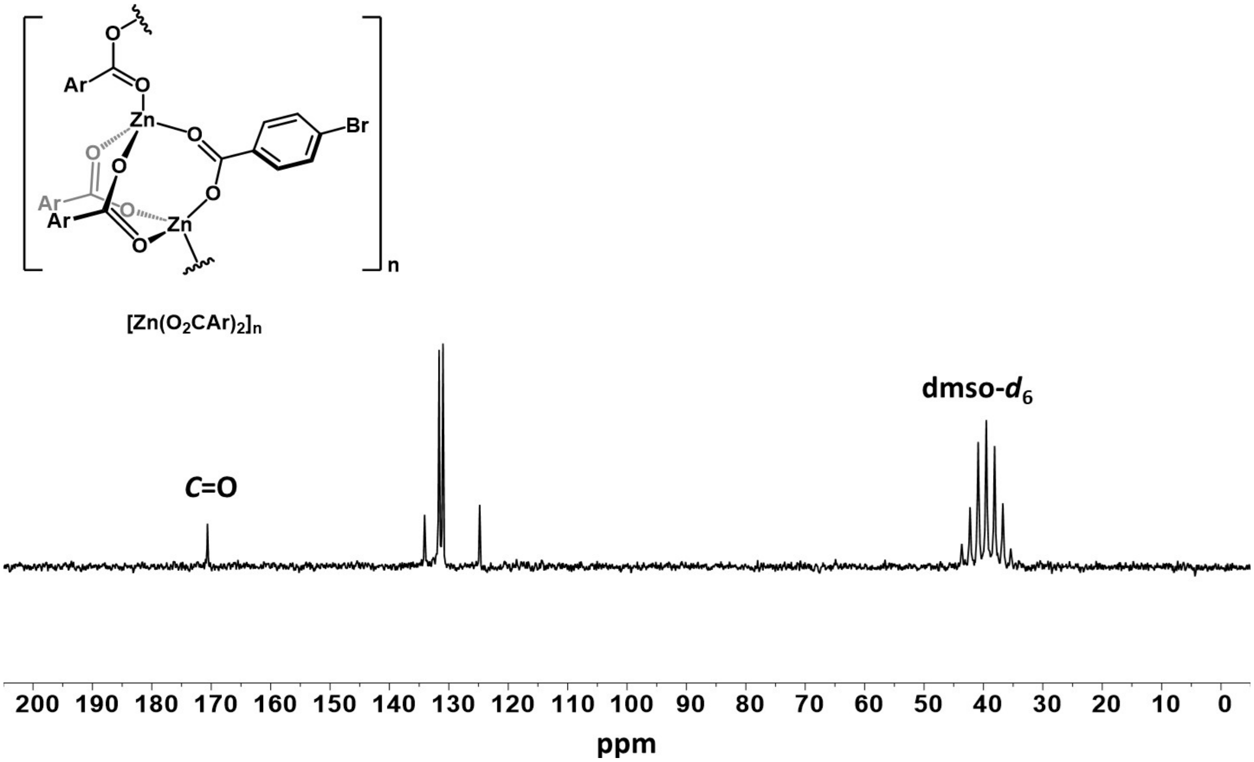

Figure S6. $^{13}C\{^1H\}$ NMR spectrum (dmso-d_6, 25 °C, 15 MHz) of $[Zn(O_2CAr)_2]_n$ (Ar = *p*-C_6H_4Br).

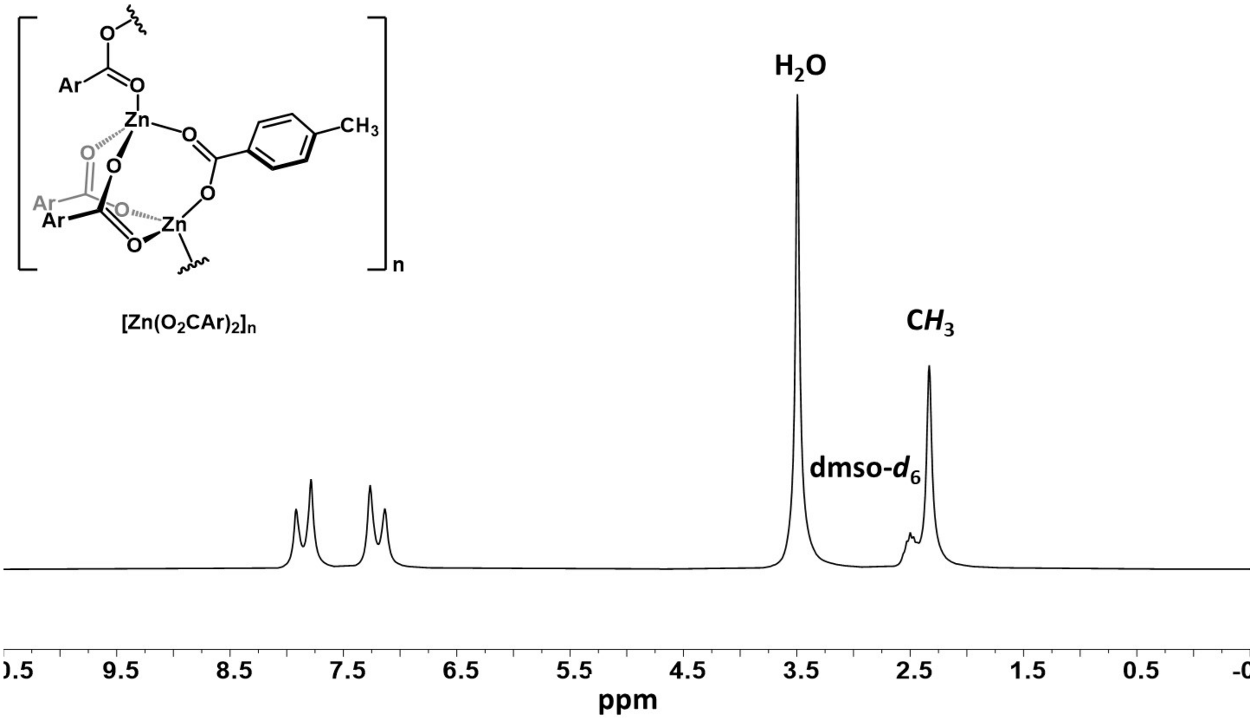

Figure S7. ^{1}H NMR spectrum (dmso-d_6, 25 °C, 60 MHz) of $[Zn(O_2CAr)_2]_n$ (Ar = p-$C_6H_4CH_3$).

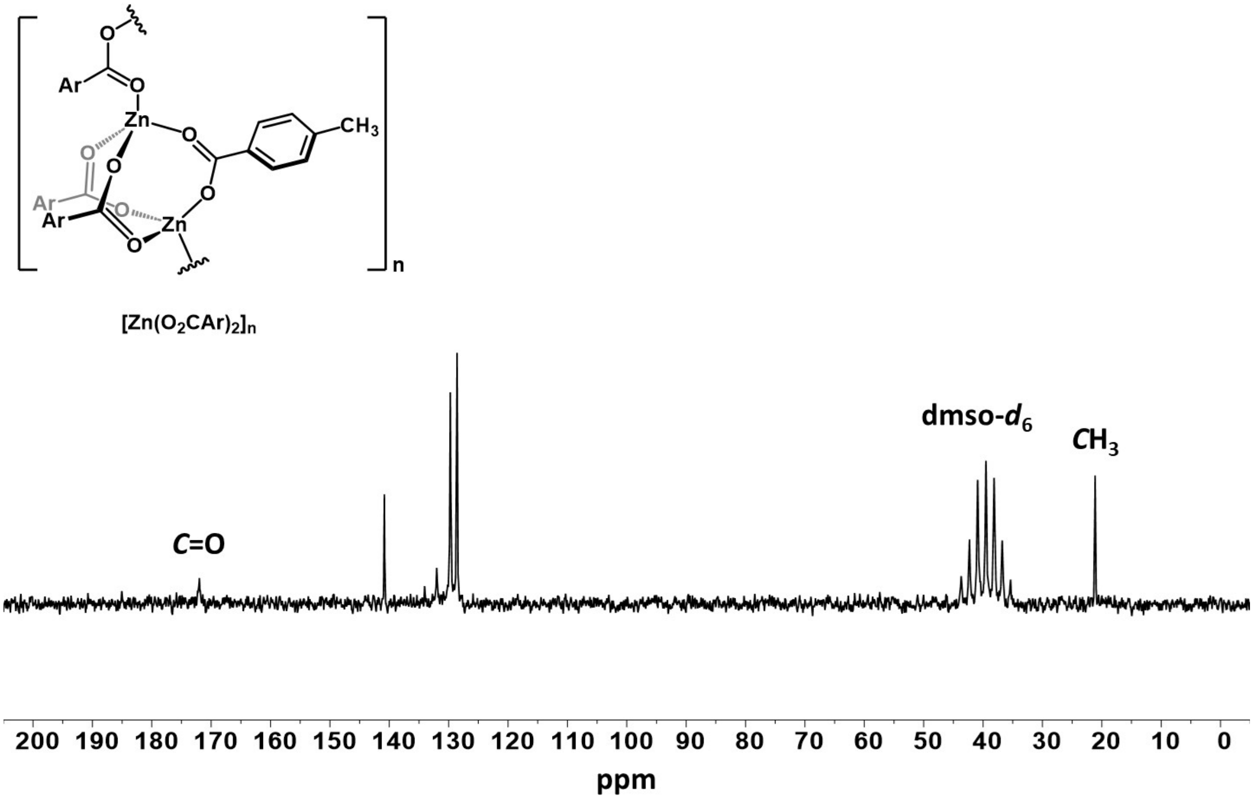

Figure S8. $^{13}C\{^{1}H\}$ NMR spectrum (dmso-d_6, 25 °C, 15 MHz) of $[Zn(O_2CAr)_2]_n$ (Ar = p-$C_6H_4CH_3$).

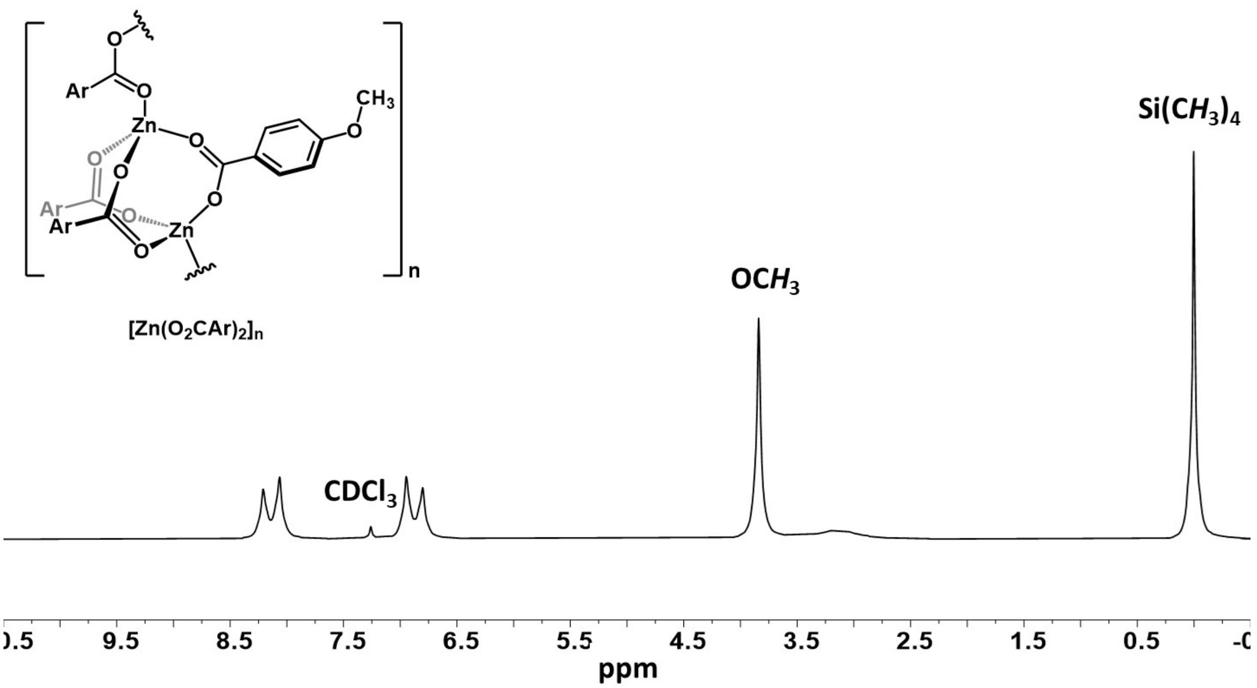

Figure S9. ^{1}H NMR spectrum ($CDCl_3$, 25 °C, 60 MHz) of $[Zn(O_2CAr)_2]_n$ (Ar = *p*-$C_6H_4OCH_3$).

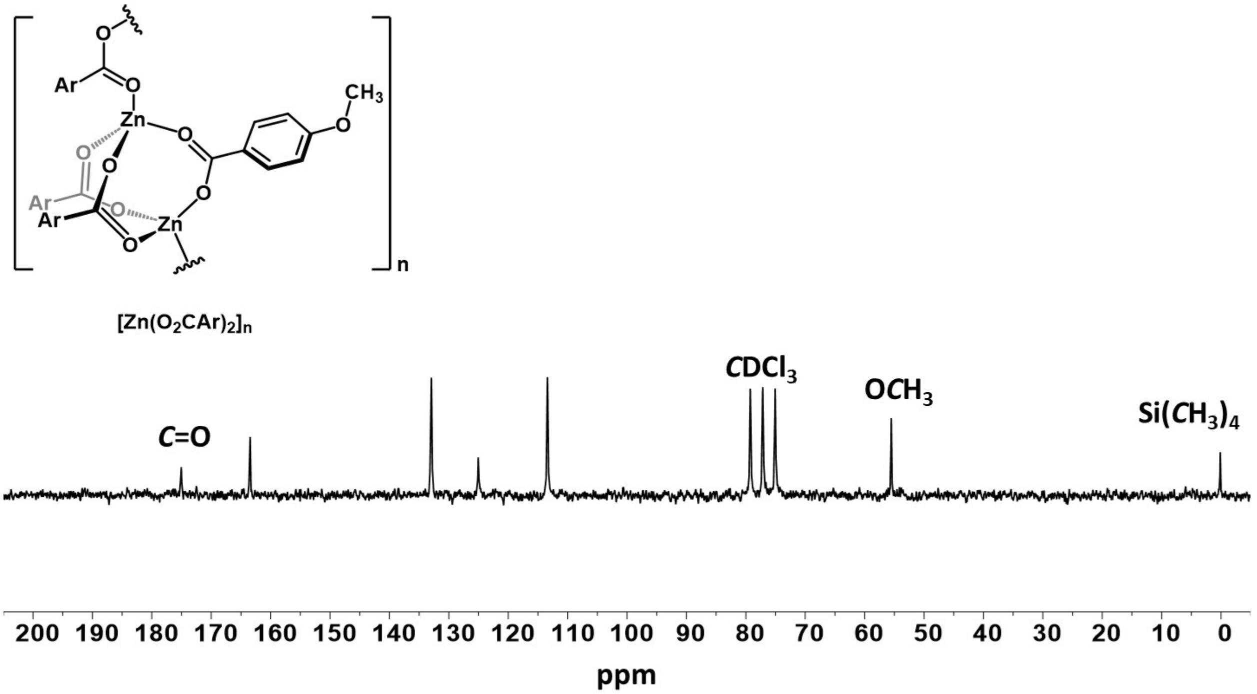

Figure S10. $^{13}C\{^1H\}$ NMR spectrum ($CDCl_3$, 25 °C, 15 MHz) of $[Zn(O_2CAr)_2]_n$ (Ar = *p*-$C_6H_4OCH_3$).

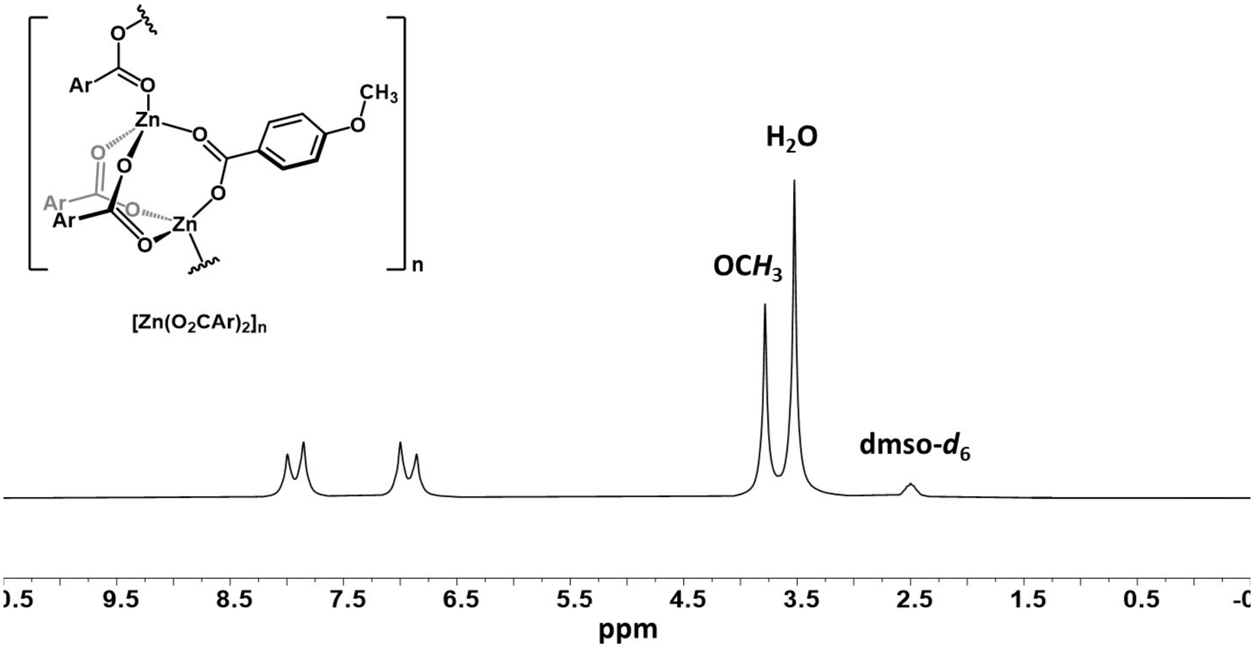

Figure S11. ^{1}H NMR spectrum (dmso-d_6, 25 °C, 60 MHz) of $[Zn(O_2CAr)_2]_n$ (Ar = p-$C_6H_4OCH_3$).

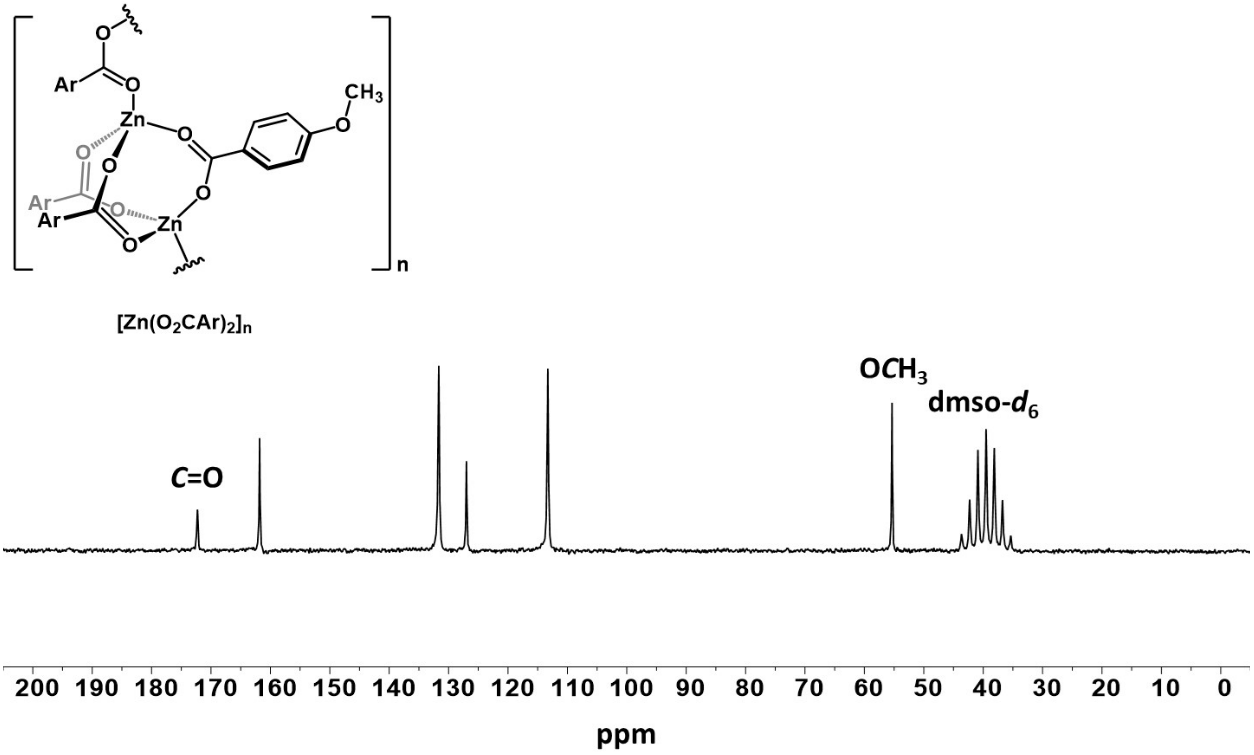

Figure S12. ^{13}C{^{1}H} NMR spectrum (dmso-d_6, 25 °C, 15 MHz) of $[Zn(O_2CAr)_2]_n$ (Ar = p-$C_6H_4OCH_3$).

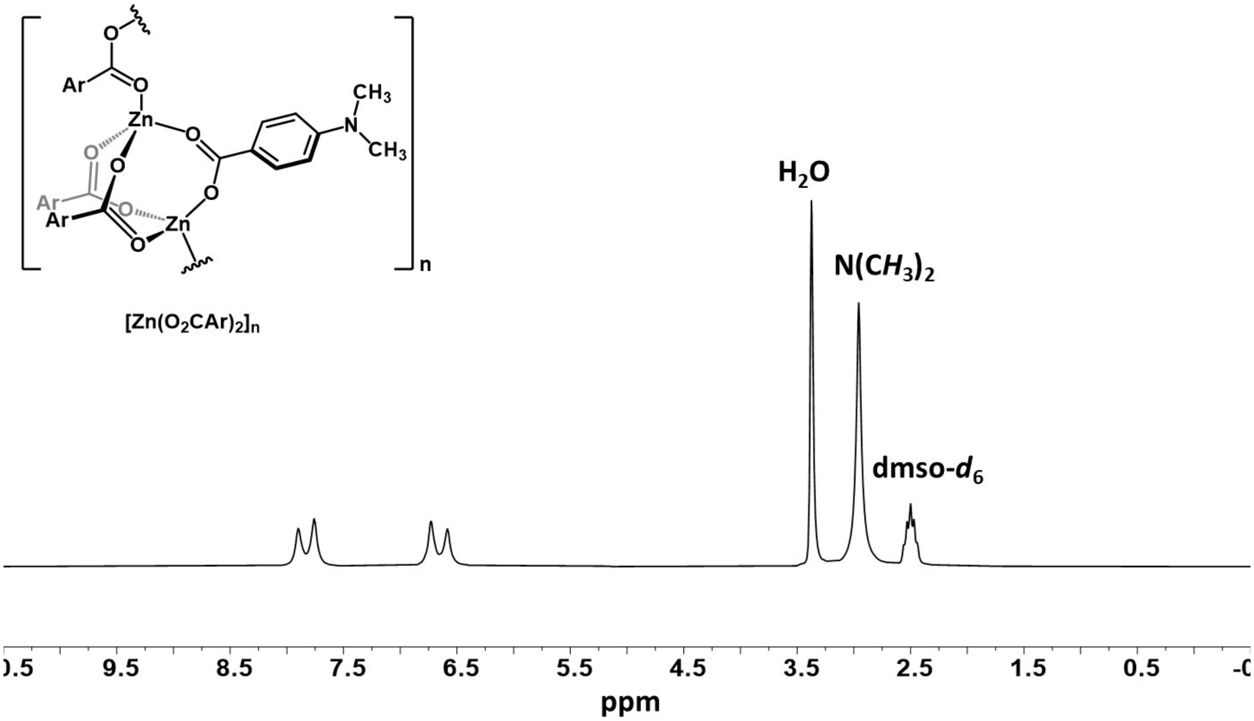

Figure S13. ^{1}H NMR spectrum (dmso-d_6, 25 °C, 60 MHz) of $[Zn(O_2CAr)_2]_n$ (Ar = *p*-$C_6H_4NMe_2$).

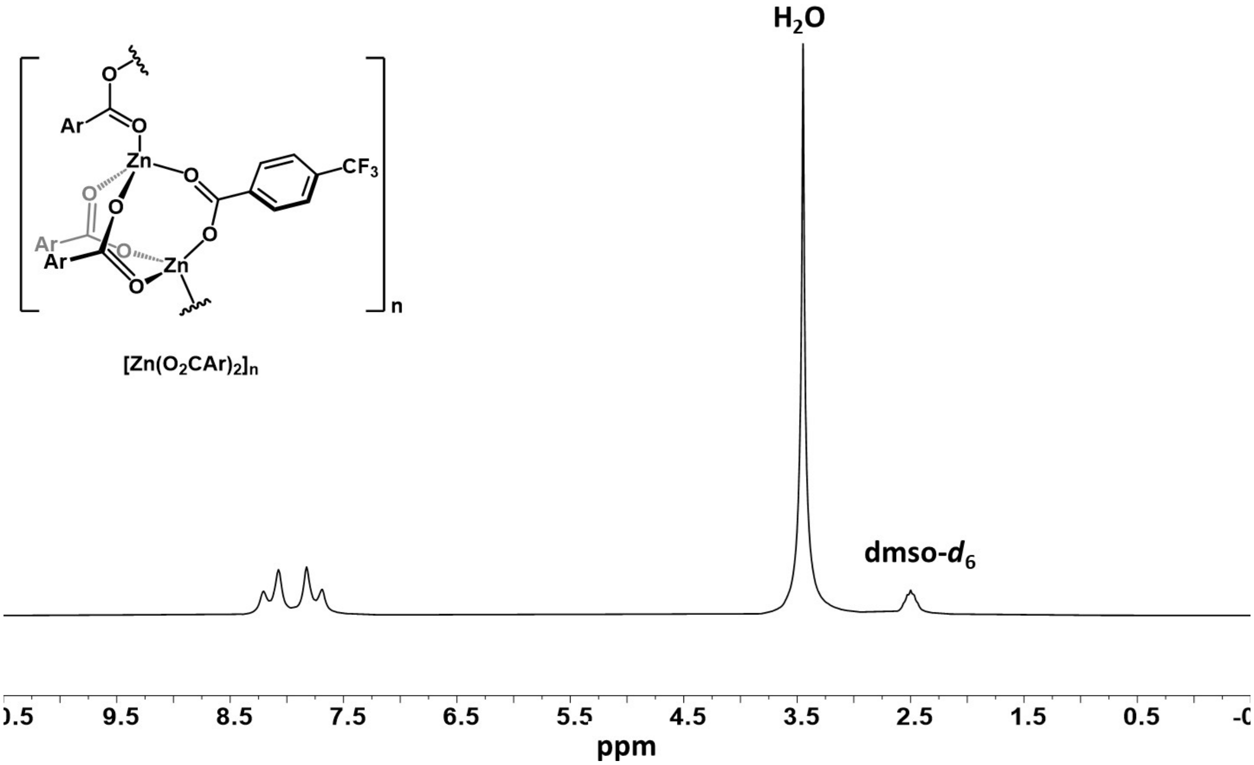

Figure S14. ^{1}H NMR spectrum (dmso-d_6, 25 °C, 60 MHz) of $[Zn(O_2CAr)_2]_n$ (Ar = *p*-$C_6H_4CF_3$).

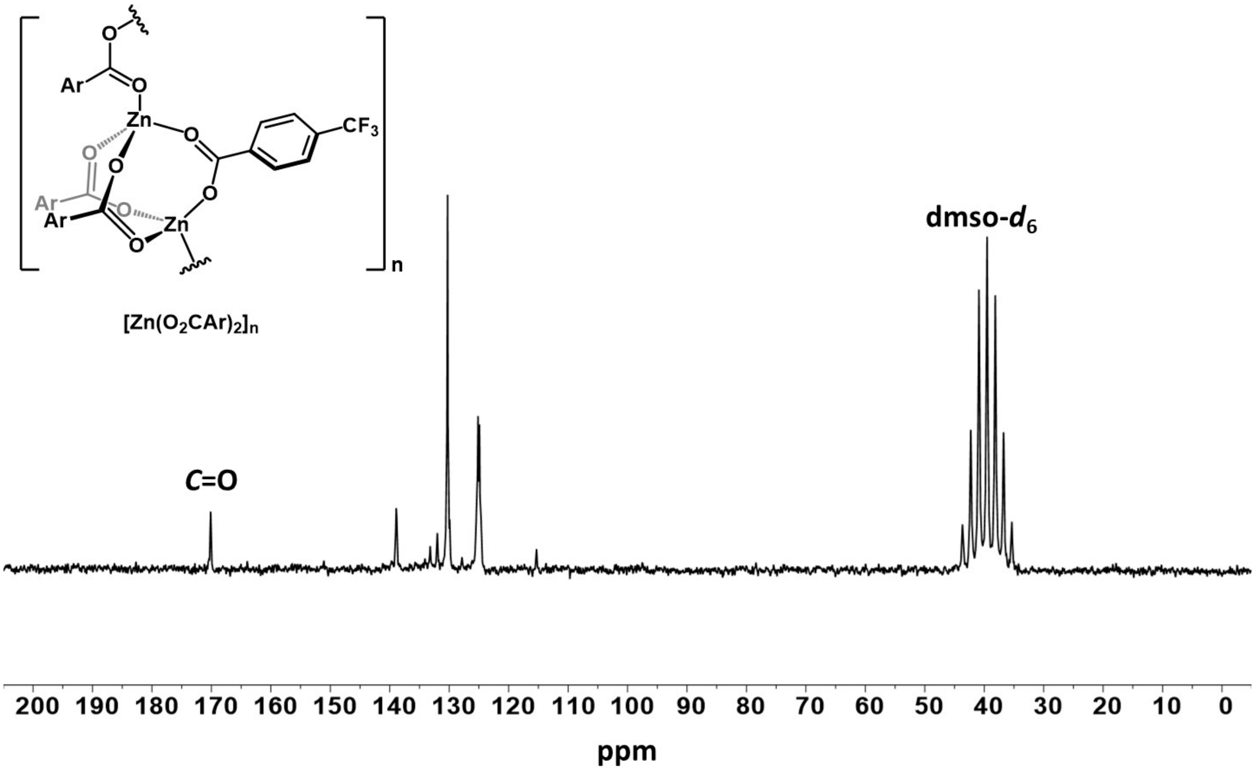

Figure S15. $^{13}C\{^1H\}$ NMR spectrum (dmso-d_6, 25 °C, 15 MHz) of $[Zn(O_2CAr)_2]_n$ (Ar = *p*-$C_6H_4CF_3$).

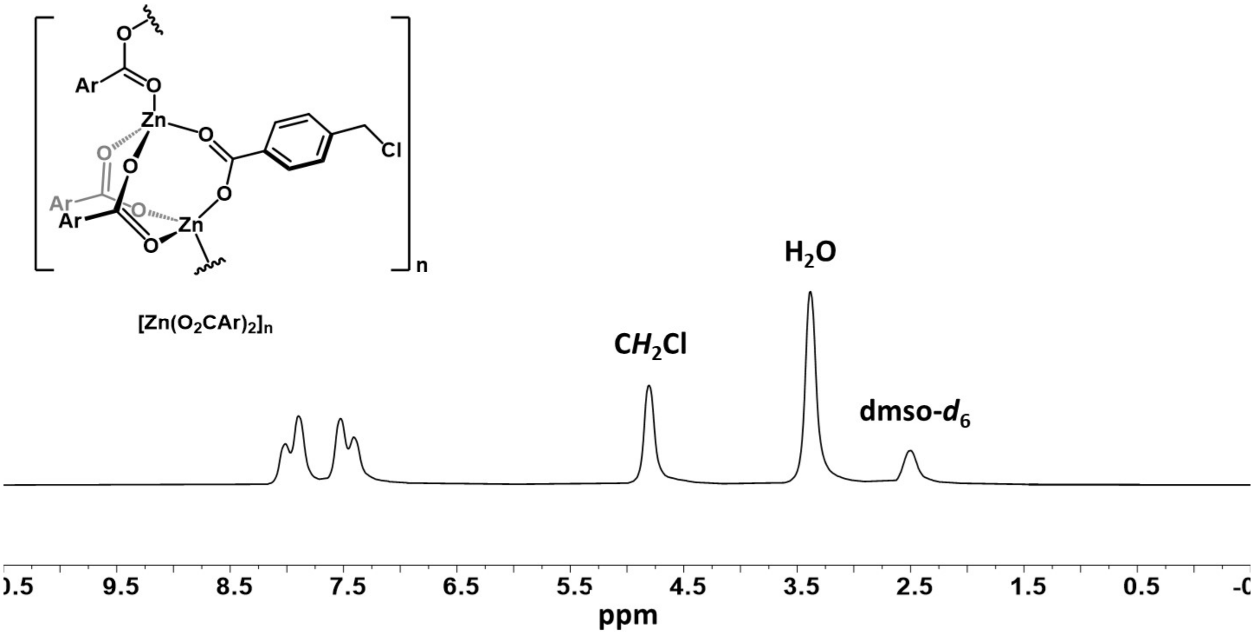

Figure S16. ^1H NMR spectrum (dmso-d_6, 25 °C, 60 MHz) of $[Zn(O_2CAr)_2]_n$ (Ar = *p*-$C_6H_4CH_2Cl$).

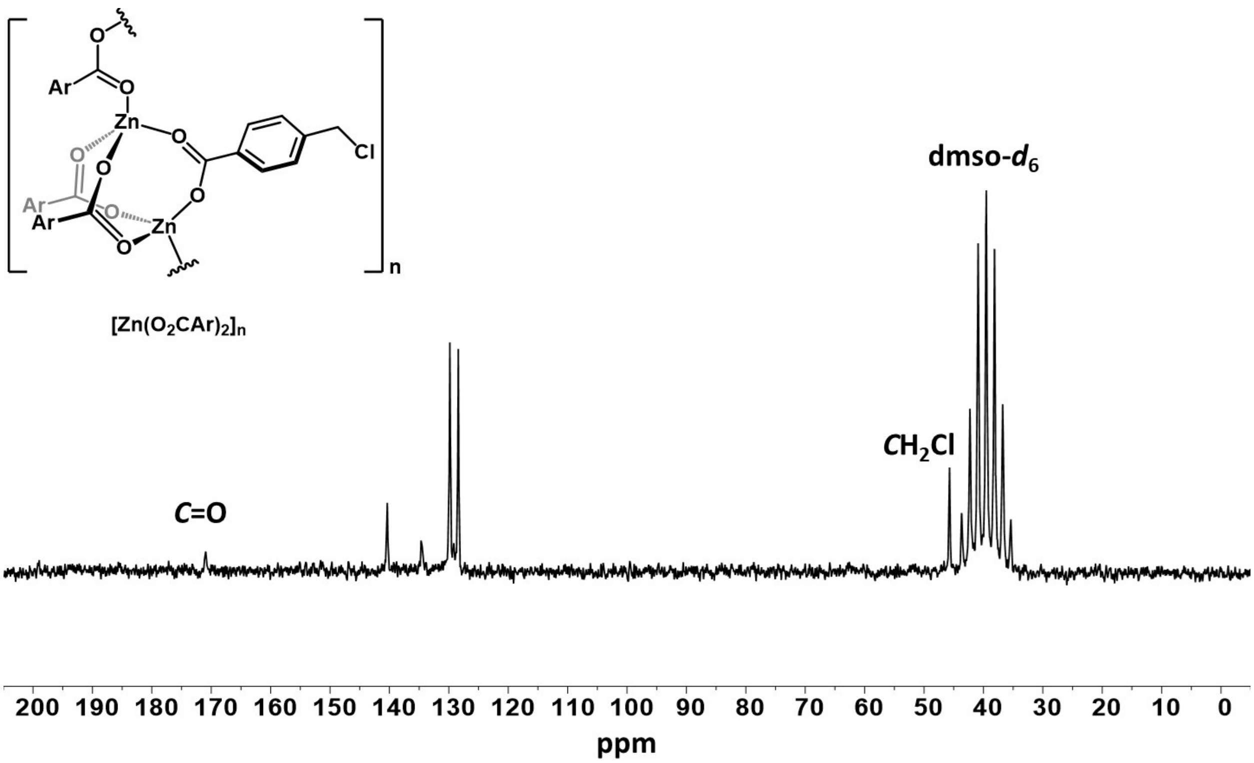

Figure S17. $^{13}C\{^{1}H\}$ NMR spectrum (dmso-d_6, 25 °C, 15 MHz) of $[Zn(O_2CAr)_2]_n$ (Ar = *p*-$C_6H_4CH_2Cl$).

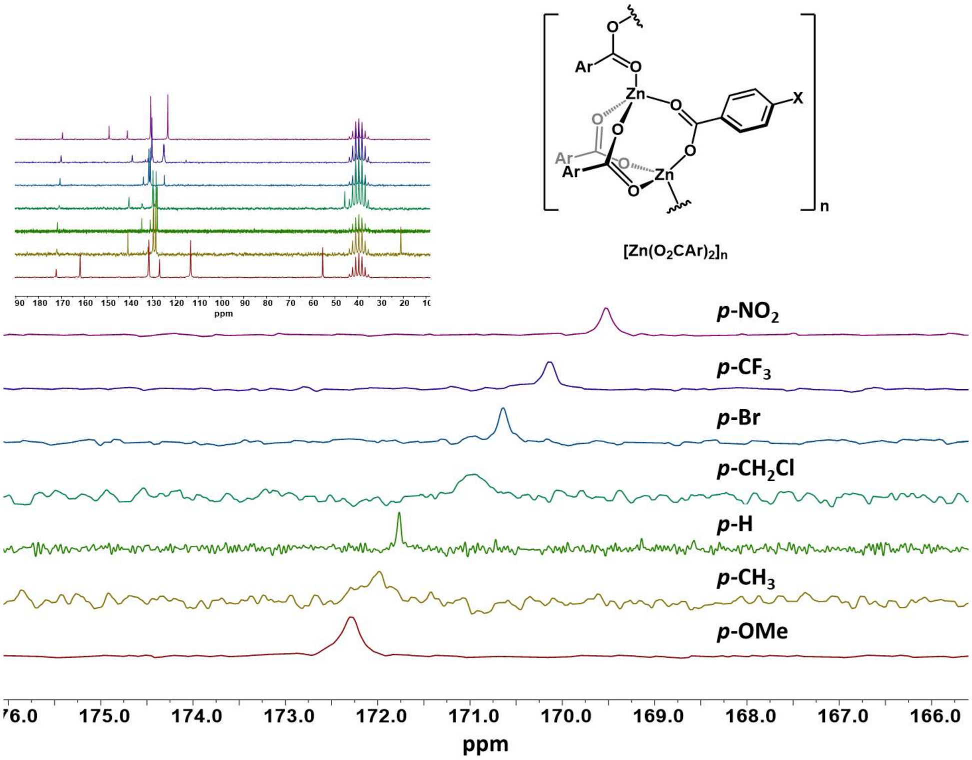

Figure S18. Overlay of $^{13}C\{^{1}H\}$ NMR spectra (dmso-d_6, 25 °C, 15 MHz) of $[Zn(O_2CAr)_2]_n$ highlighting the *C*=O resonances.

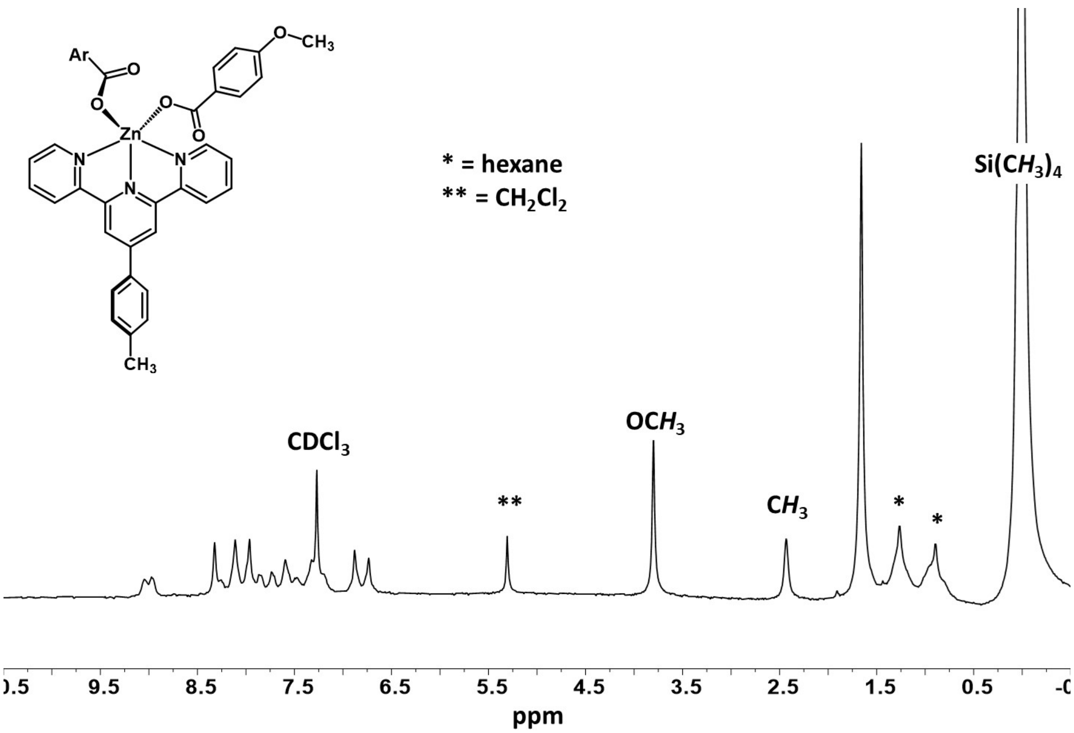

Figure S19. ^{1}H NMR spectrum ($CDCl_3$, 25 °C, 60 MHz) of $(tpy^R)Zn(O_2CAr)_2$ (Ar = *p*-C_6H_4OMe; tpy^R = 4'-(4-methylphenyl)-2,2':6',2''-terpyridine).

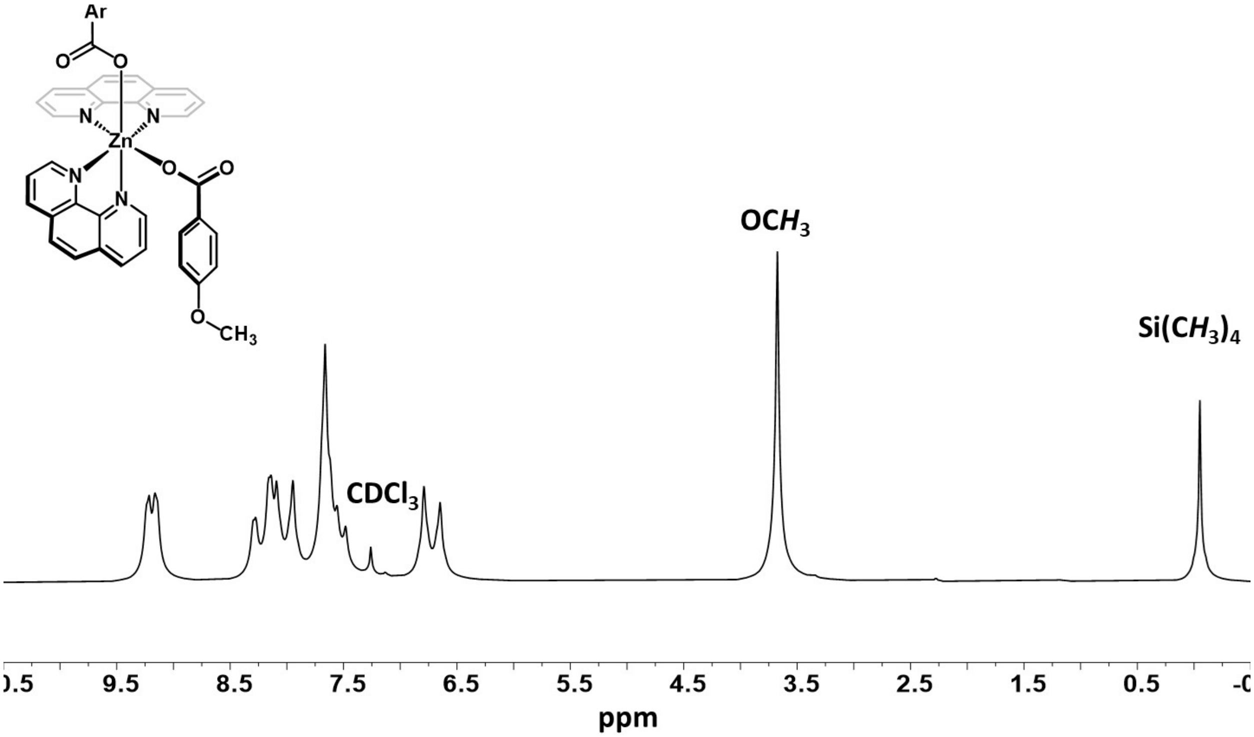

Figure S20. ^{1}H NMR spectrum ($CDCl_3$, 25 °C, 60 MHz) of $(phen)_2Zn(O_2CAr)_2$ (Ar = *p*-C_6H_4OMe; phen = 1,10-phenanthroline).

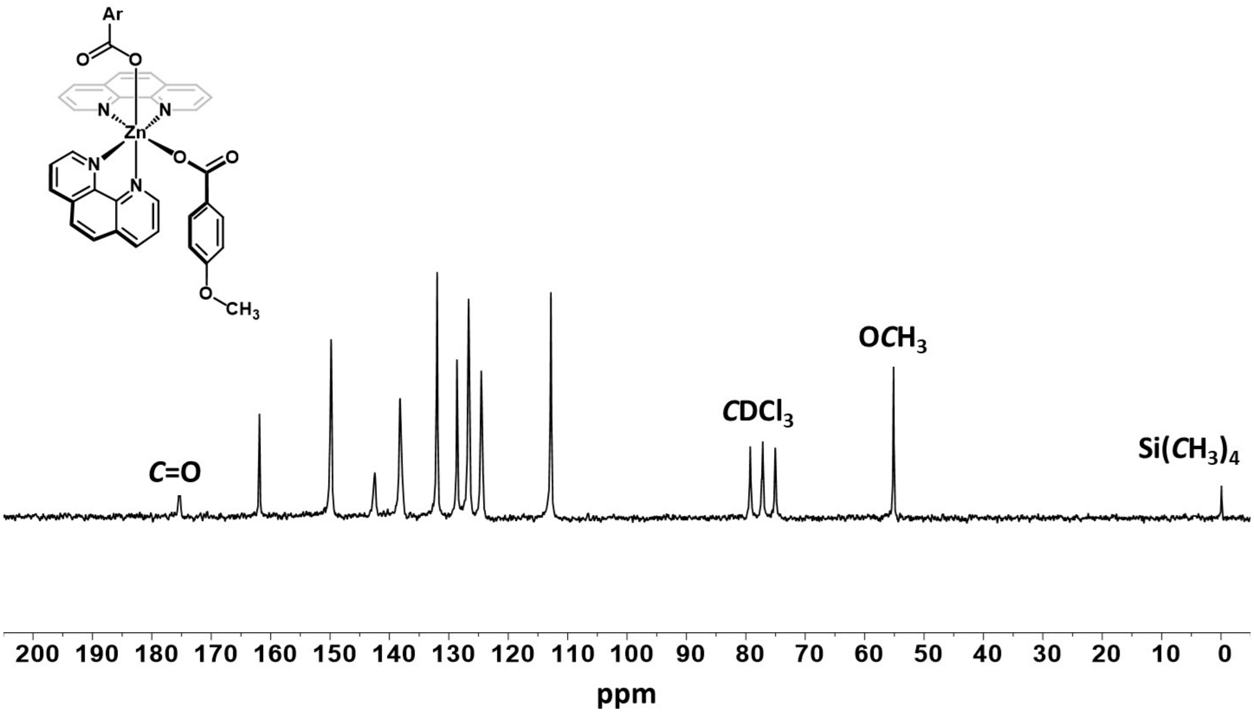

Figure S21. $^{13}C\{^1H\}$ NMR spectrum ($CDCl_3$, 25 °C, 15 MHz) of $(phen)_2Zn(O_2CAr)_2$ (Ar = *p*-C_6H_4OMe; phen = 1,10-phenanthroline).

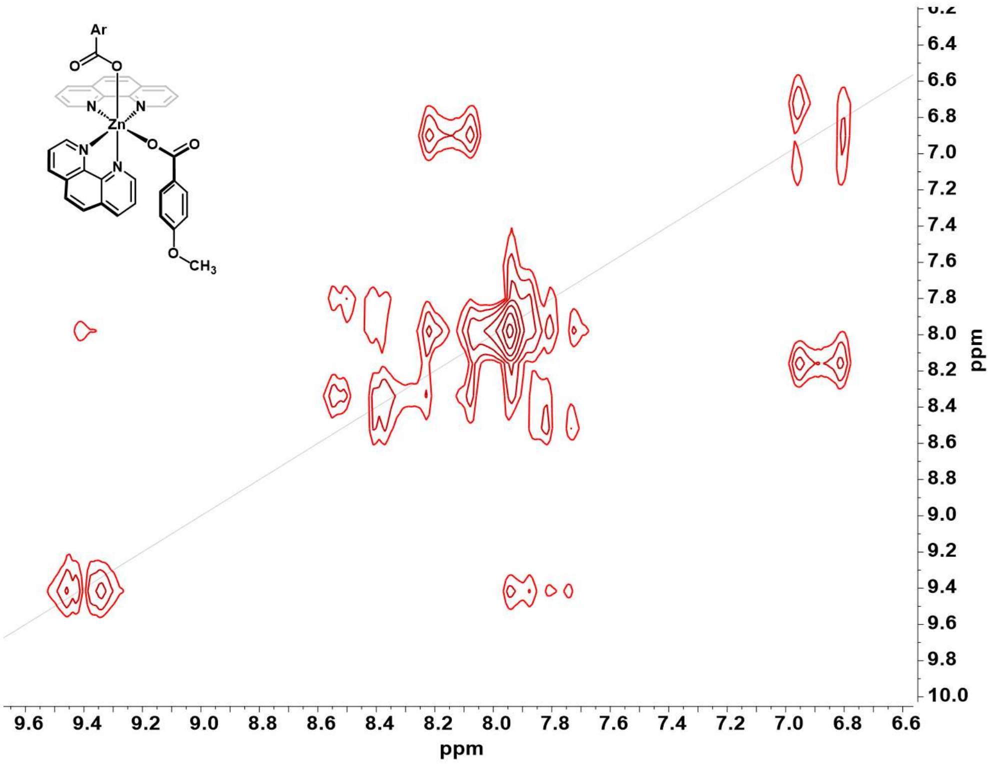

Figure S22. ^1H-^1H COSY spectrum ($CDCl_3$, 25 °C, 60 MHz) of $(phen)_2Zn(O_2CAr)_2$ (Ar = *p*-C_6H_4OMe; phen = 1,10-phenanthroline) highlighting the aromatic region.

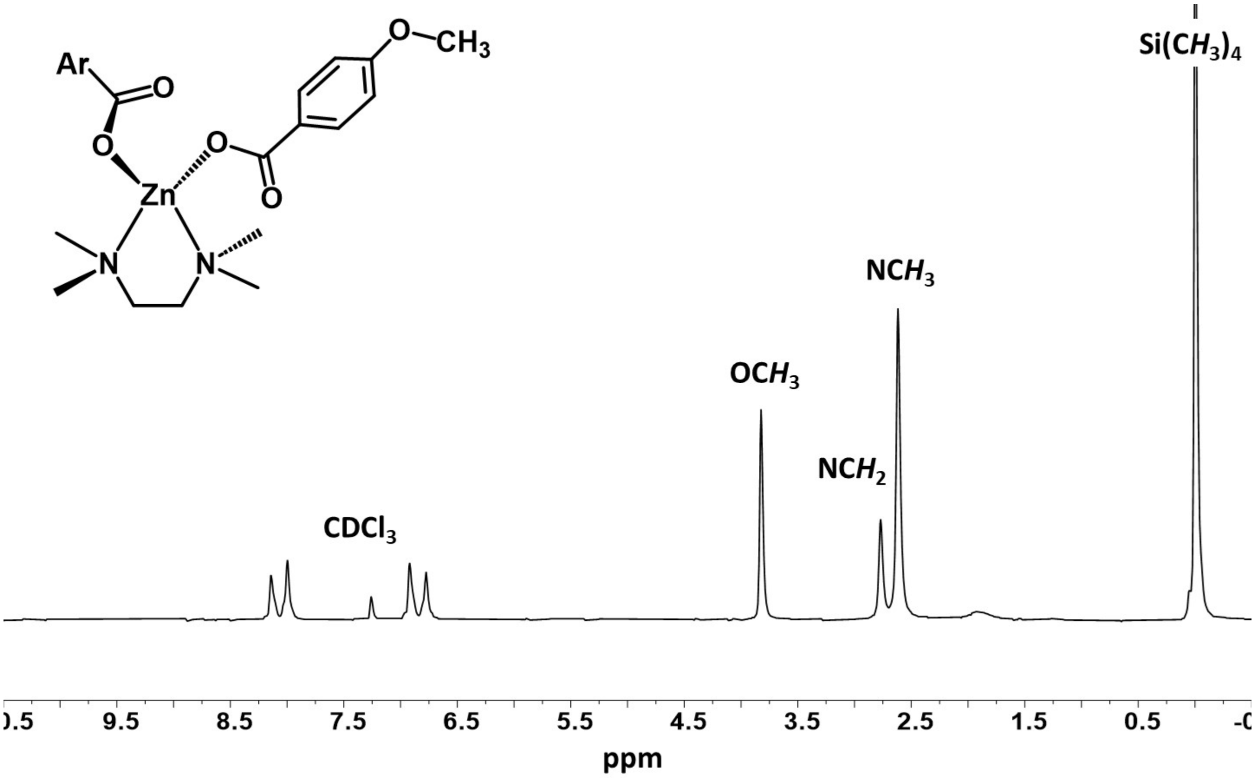

Figure S23. ^{1}H NMR spectrum ($CDCl_3$, 25 °C, 60 MHz) of (tmeda)Zn(O_2CAr)$_2$ (Ar = *p*-C_6H_4OMe; tmeda = N,N,N',N'-tetramethylethylenediamine).

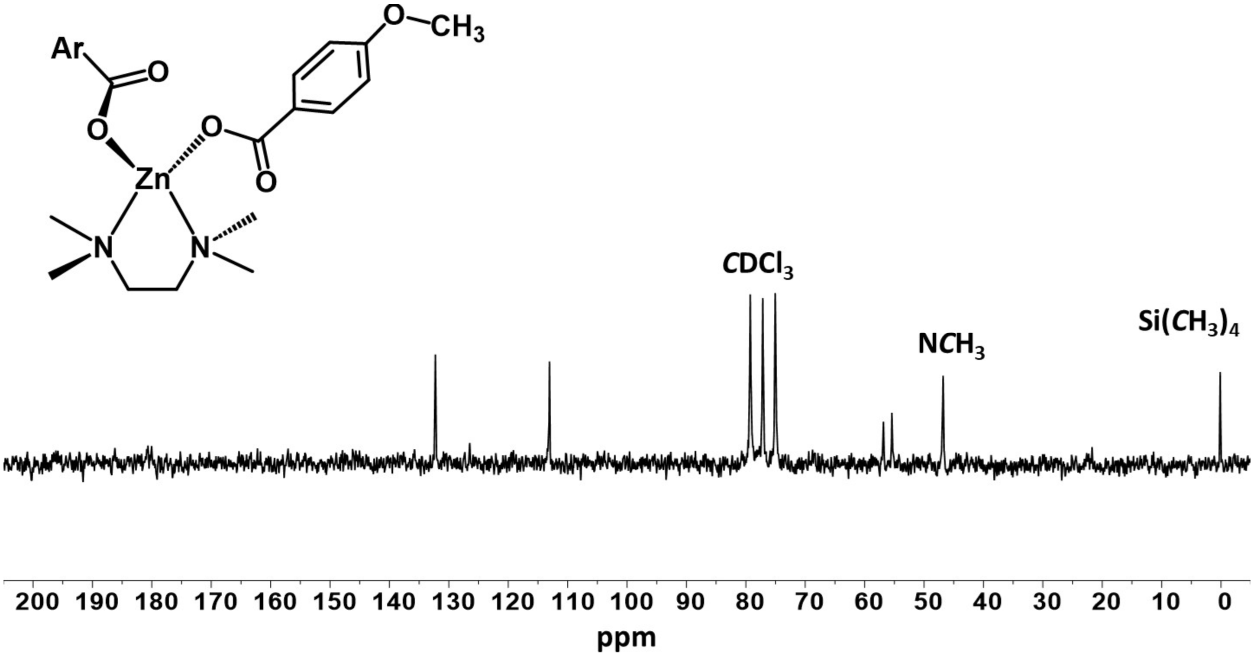

Figure S24. $^{13}C\{^{1}H\}$ NMR spectrum ($CDCl_3$, 25 °C, 15 MHz) of (tmeda)Zn(O_2CAr)$_2$ (Ar = *p*-C_6H_4OMe; tmeda = N,N,N',N'-tetramethylethylenediamine).

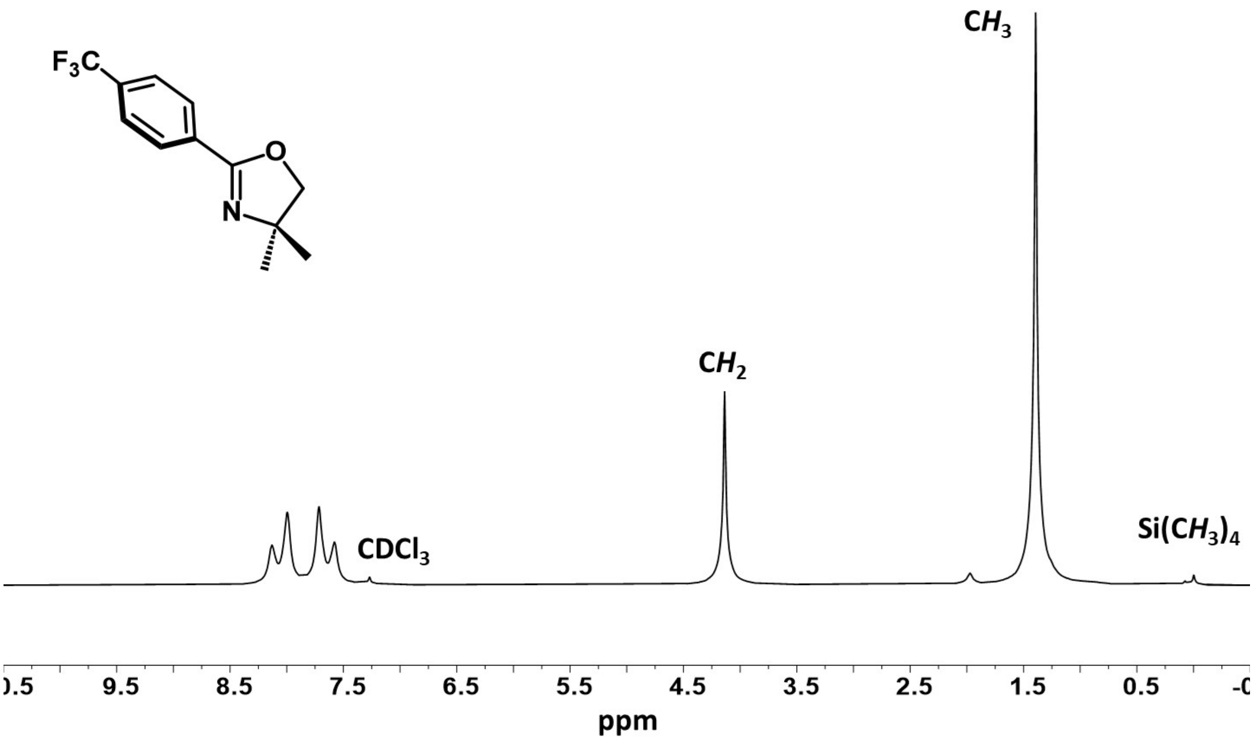

Figure S25. ^{1}H NMR spectrum ($CDCl_3$, 25 °C, 60 MHz) of 4,4'-dimethyl-2-(4-trifluoromethyl)phenyl-4,5-dihydrooxazole.

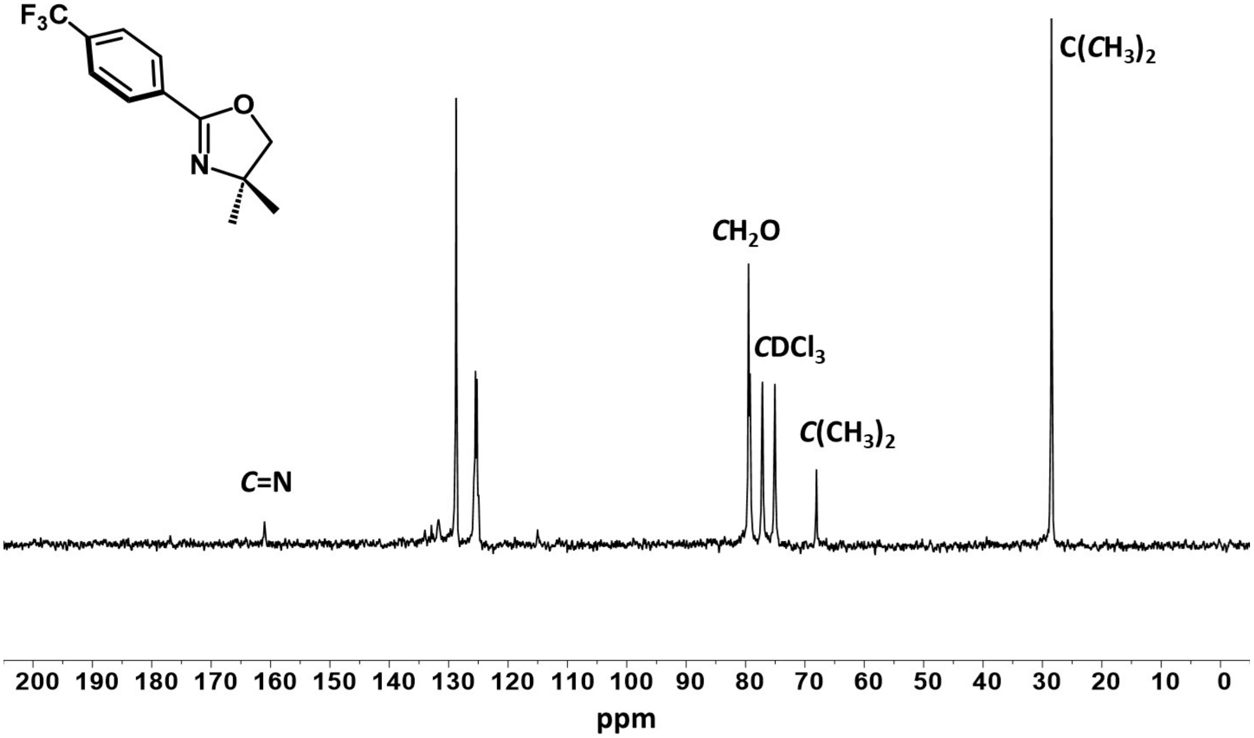

Figure S26. $^{13}C\{^{1}H\}$ NMR spectrum ($CDCl_3$, 25 °C, 15 MHz) of 4,4'-dimethyl-2-(4-trifluoromethyl)phenyl-4,5-dihydrooxazole.

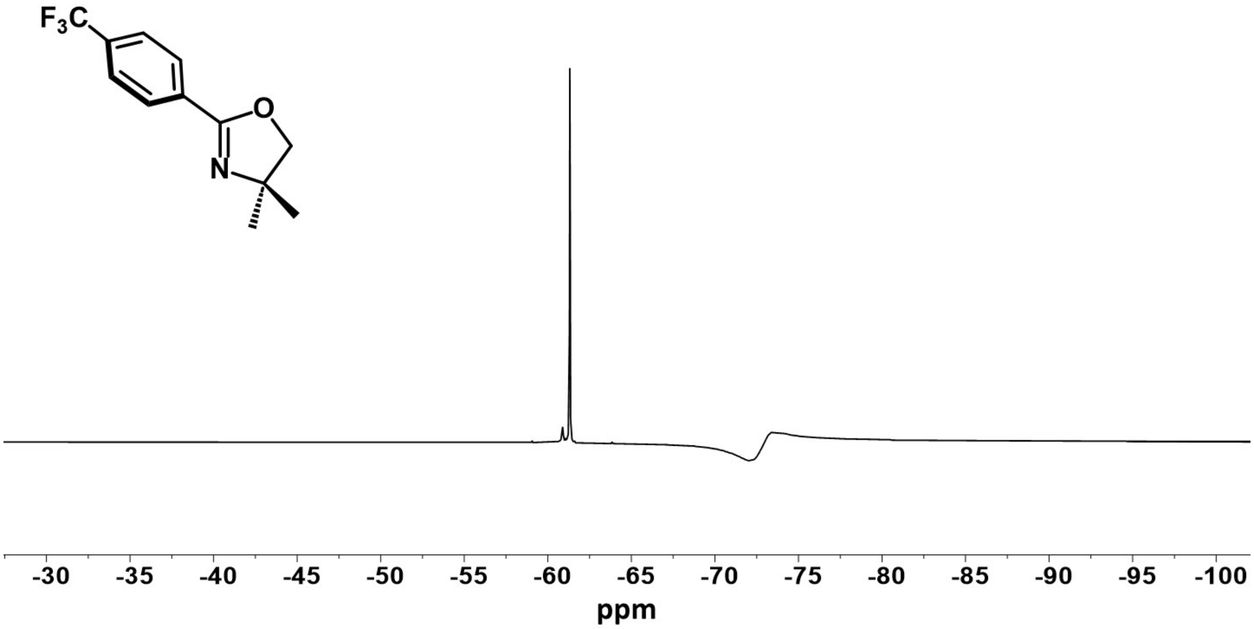

Figure S27. ^{19}F NMR spectrum ($CDCl_3$, 25 °C, 56 MHz) of 4,4'-dimethyl-2-(4-trifluoromethyl)phenyl-4,5-dihydrooxazole.

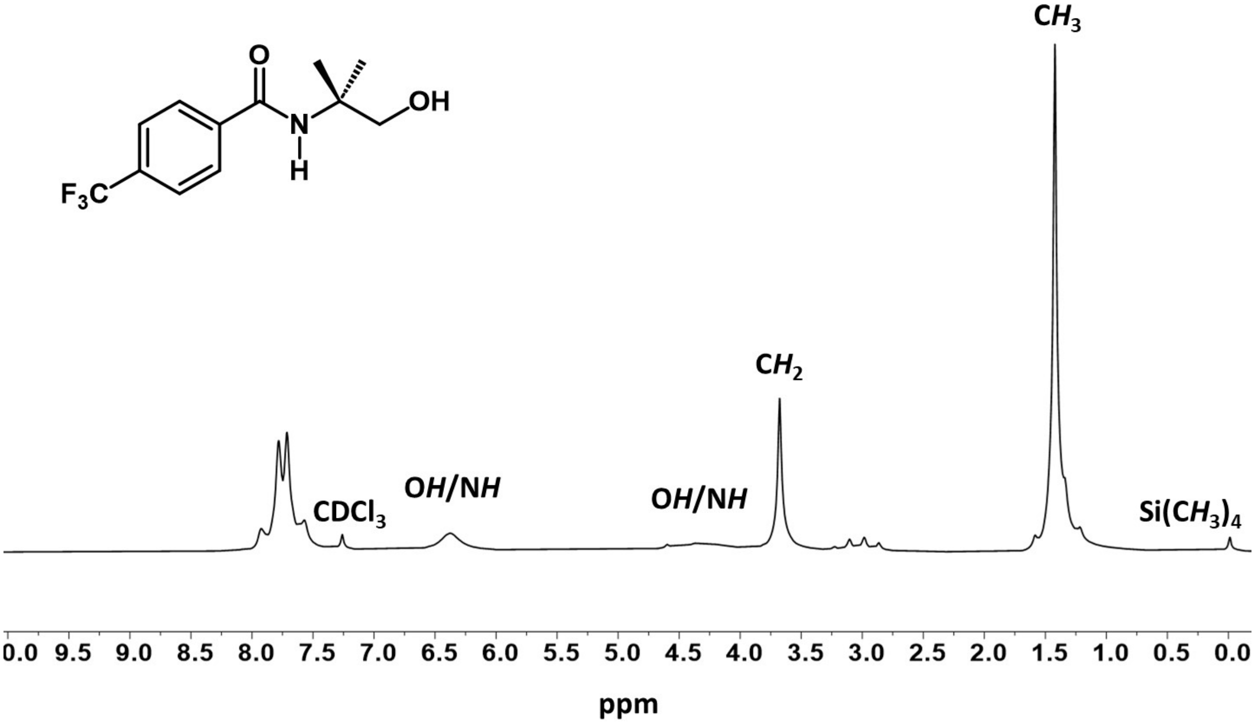

Figure S28. ^{1}H NMR spectrum ($CDCl_3$, 25 °C, 60 MHz) of *N*-(2-hydroxy-1,1-dimethylethyl)-4-(trifluoromethyl)benzamide.

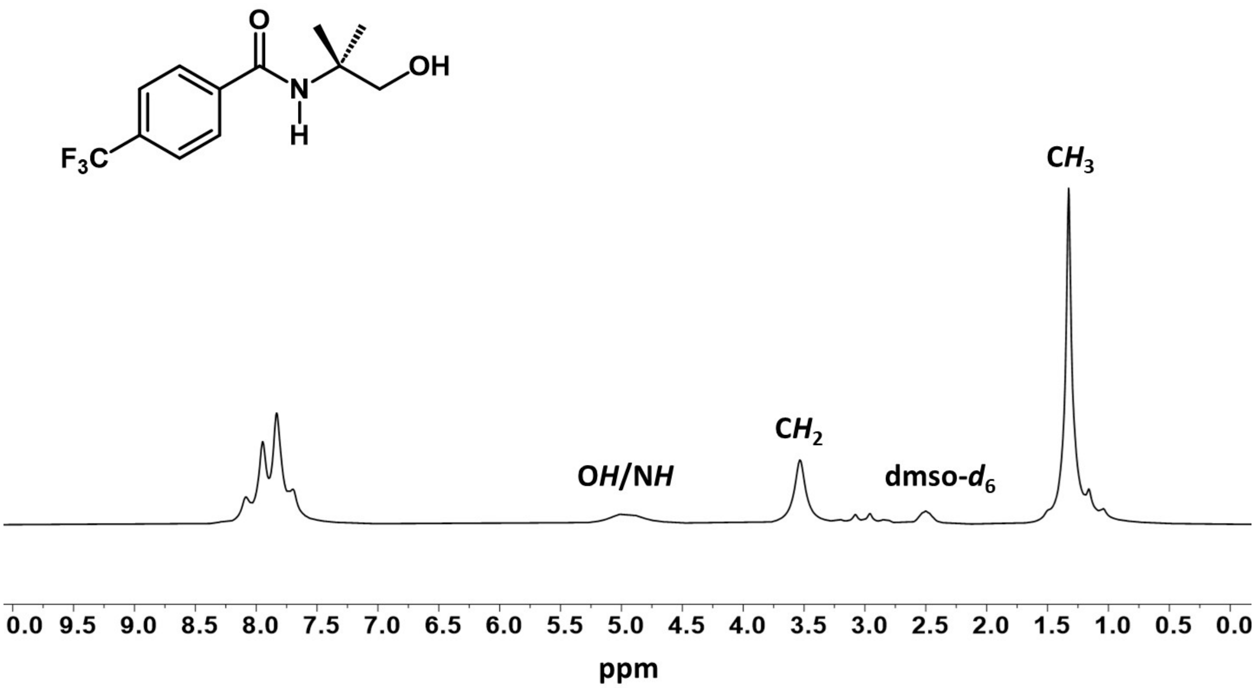

Figure S29. ^{1}H NMR spectrum (dmso-d_6, 25 °C, 60 MHz) of *N*-(2-hydroxy-1,1-dimethylethyl)-4-(trifluoromethyl)benzamide.

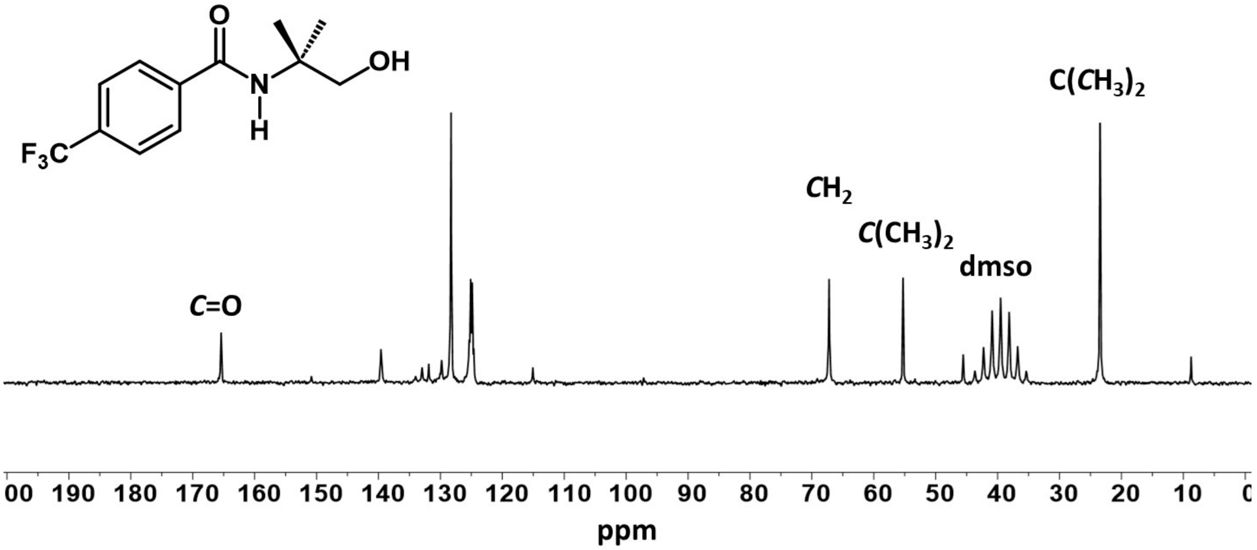

Figure S30. $^{13}C\{^{1}H\}$ NMR spectrum (dmso-d_6, 25 °C, 15 MHz) of *N*-(2-hydroxy-1,1-dimethylethyl)-4-(trifluoromethyl)benzamide.

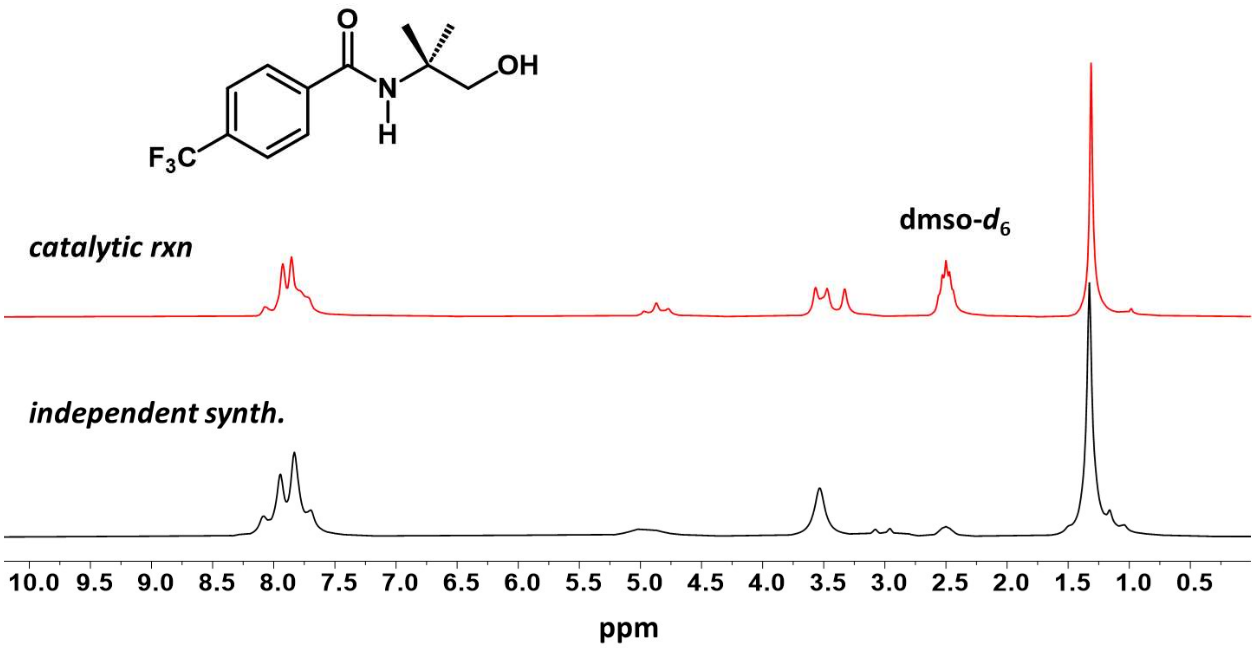

Figure S31. ^{1}H NMR spectra (dmso-d_6, 25 °C, 60 MHz) of *N*-(2-hydroxy-1,1-dimethylethyl)-4-(trifluoromethyl)benzamide derived from a catalytic trial (top) and from independent synthesis (bottom).

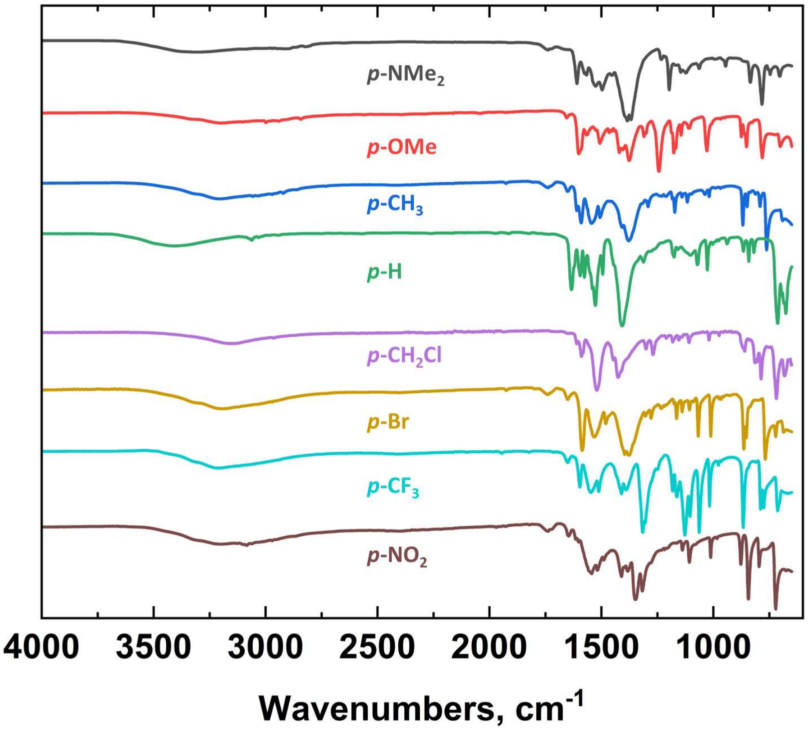

Figure S32. Infrared spectra (ATR, neat, room temperature) of $[Zn(O_2CAr)_2]_n$.

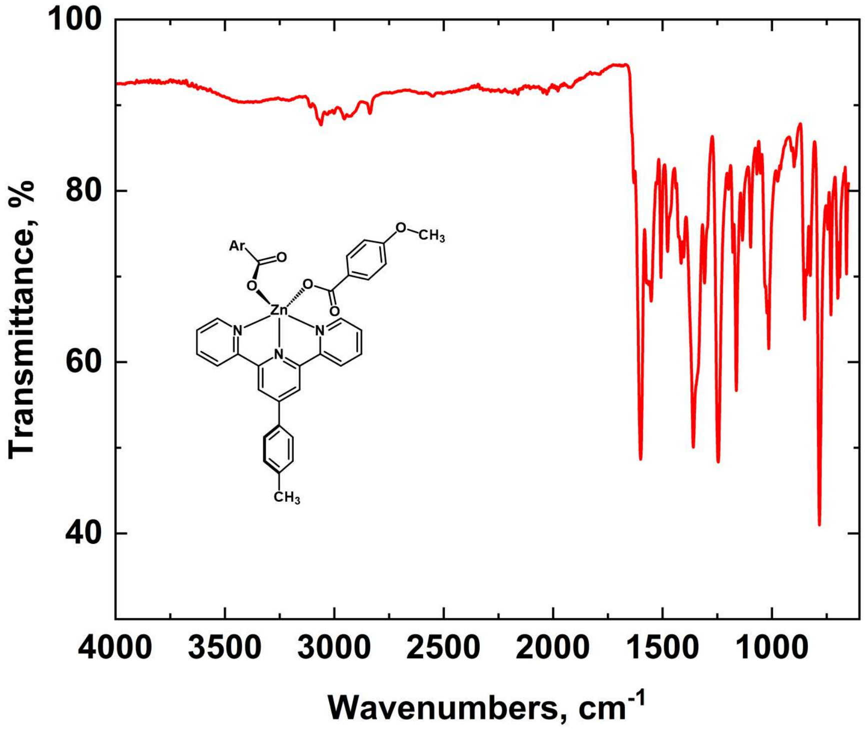

Figure S33. Infrared spectrum (ATR, neat, room temperature) of (tpy^R)Zn(O_2CAr)$_2$ (Ar = *p*-C_6H_4OMe; tpy^R = 4’-(4-methylphenyl)-2,2’:6’,2’’-terpyridine).

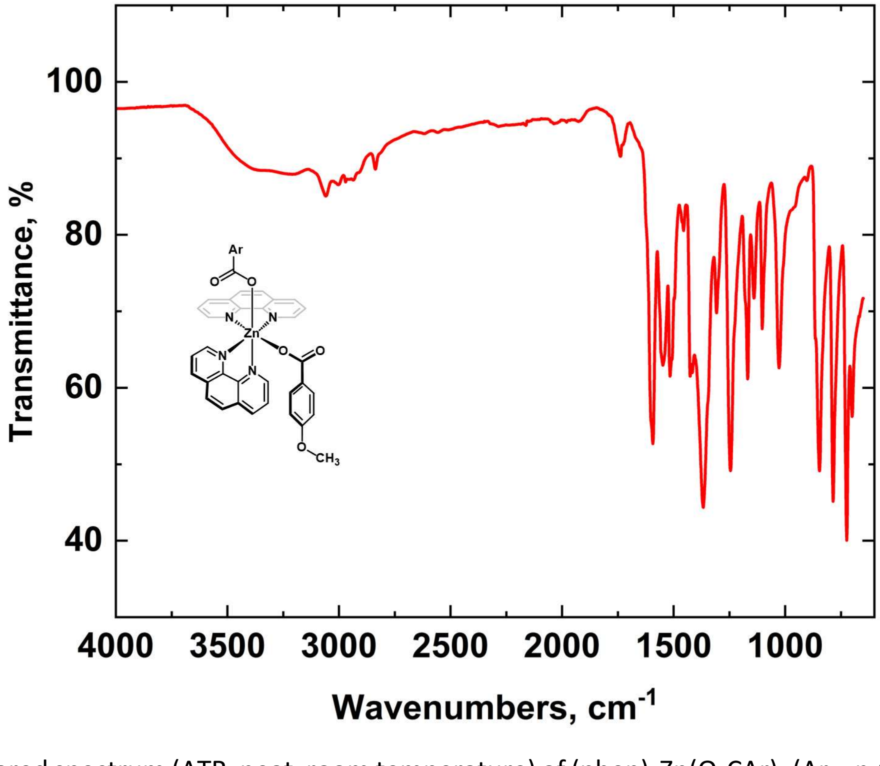

Figure S34. Infrared spectrum (ATR, neat, room temperature) of (phen)$_2$Zn(O_2CAr)$_2$ (Ar = *p*-C_6H_4OMe; phen = 1,10-phenanthroline).

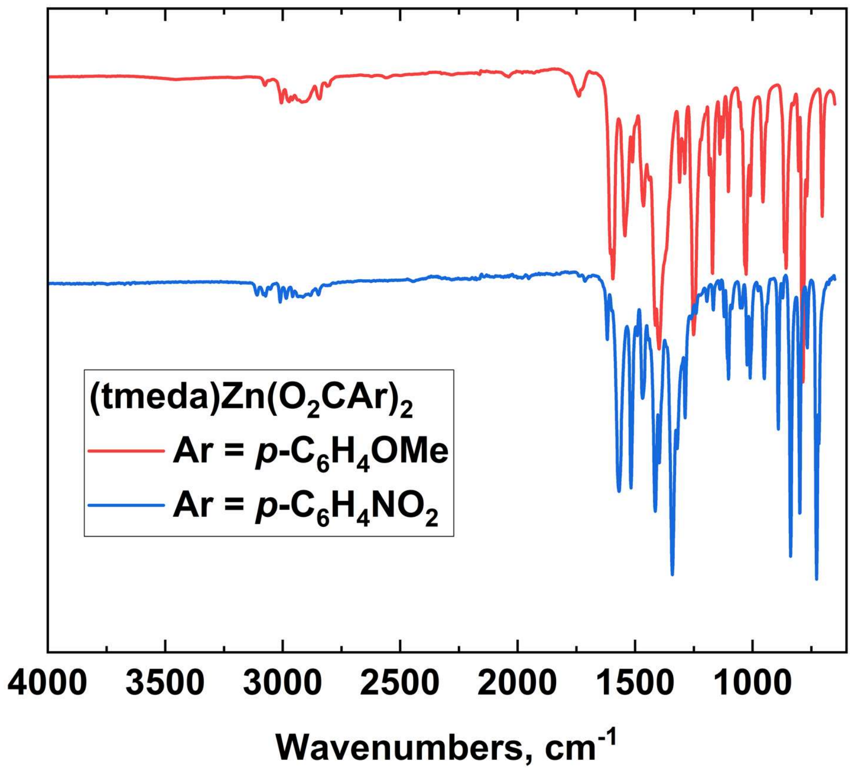

Figure S35. Infrared spectra (ATR, neat, room temperature) of $(tmeda)_2Zn(O_2CAr)_2$ (tmeda = N,N,N',N'-tetramethylethylenediamine).

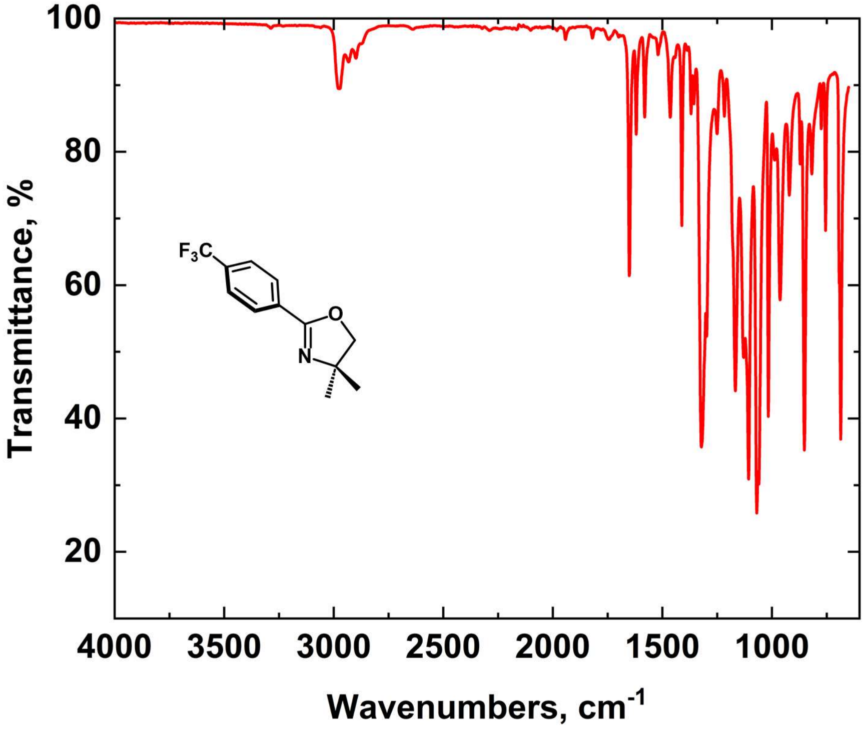

Figure S36. Infrared spectrum (ATR, neat, room temperature) of 4,4'-dimethyl-2-(4-trifluoromethyl)phenyl-4,5-dihydrooxazole.

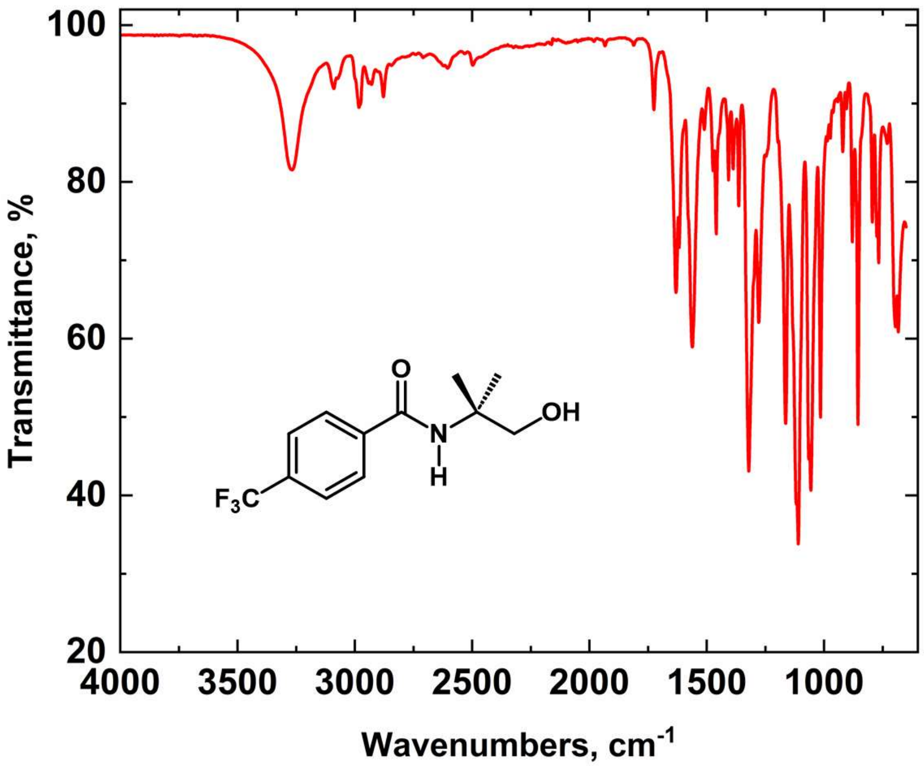

Figure S37. Infrared spectrum (ATR, neat, room temperature) of *N*-(2-hydroxy-1,1-dimethylethyl)-4-(trifluoromethyl)benzamide.

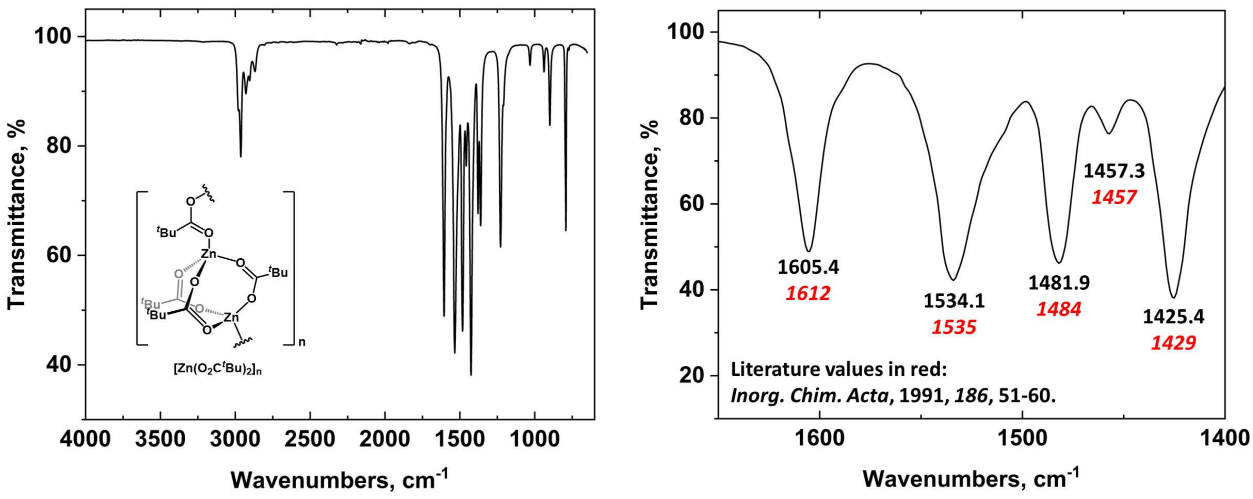

Figure S38. Infrared spectrum (ATR, neat, room temperature) of $[Zn(O_2C^tBu)_2]_n$. Left: full spectrum. Right: zoomed in for literature comparison (black values = this report; red values = literature report).

Table S2. Summary of catalytic reactions.

Catalyst	Trial	^{19}F NMR Yield	Catalyst	Trial	^{19}F NMR Yield
p-NMe_2	Trial #1	58%	*p*-Br	Trial #1	51%
	Trial #2	35%		Trial #2	45%
	Trial #3	35%		Trial #3	45%
	Trial #4	39%		Trial #4	57%
	Trial #5	31%		Ave/error:	50% +/- 5%
	Ave/error:	40% +/- 10%			
			p-CF_3	Trial #1	34%
p-OMe	Trial #1	47%		Trial #2	40%
	Trial #2	69%		Trial #3	58%
	Trial #3	51%		Trial #4	42%
	Trial #4	52%		Trial #5	51%
	Ave/error:	55% +/- 8%		Ave/error:	45% +/- 8%
p-CH_3	Trial #1	47%	*p*-NO_2	Trial #1	29%
	Trial #2	43%		Trial #2	25%
	Trial #3	50%		Trial #3	31%
	Trial #4	35%		Trial #4	32%
	Ave/error:	44% +/- 6%		Ave/error:	29% +/- 3%
p-CH_2Cl	Trial #1	27%	$Zn(OAc)_2$	Trial #1	40%
	Trial #2	40%		Trial #2	29%
	Trial #3	25%		Trial #3	29%
	Trial #4	25%		Trial #4	38%
	Ave/error:	29% +/- 6%		Ave/error:	34% +/- 5%
p-H	Trial #1	53%	No Catalyst	Trial #1	5%
	Trial #2	67%		Trial #2	3%
	Trial #3	37%		Trial #3	7%
	Trial #4	46%		Trial #4	5%
	Trial #5	51%		Ave/error:	5% +/- 1%
	Trial #6	39%			
	Ave/error:	49% +/- 10%			

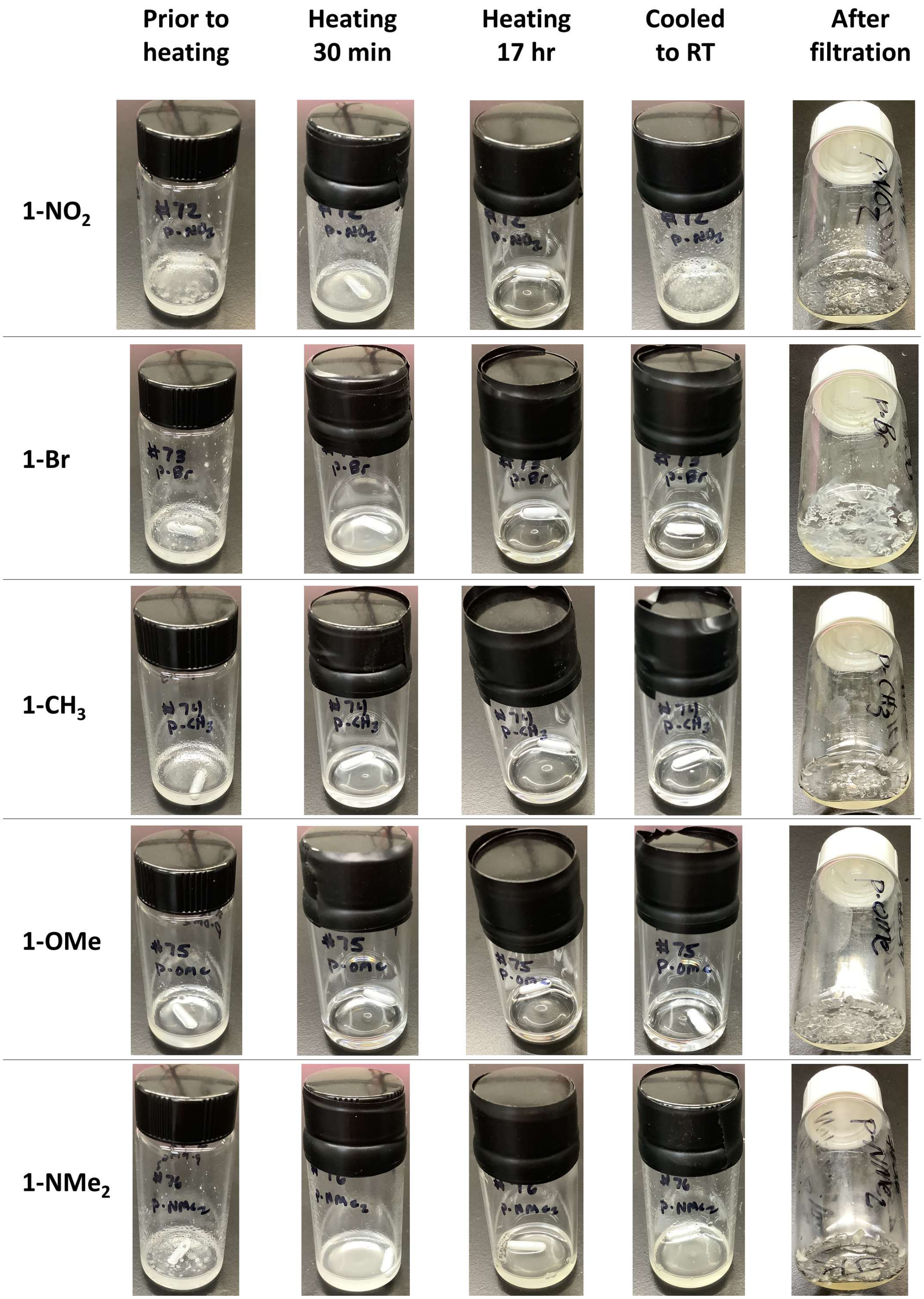

Figure S39. Qualitative observations during catalytic reactions.

Qualitative observations during catalytic reactions

Catalytic reactions involving **1-NO_2**, **1-Br**, **1-CH_3**, **1-OMe**, and **1-NMe_2** were monitored qualitatively for solubility observations. Below are listed observations at specific reaction times.

1) Prior to heating
   a) **1-NO_2**: Catalyst not soluble
   b) **1-Br**: Catalyst not soluble
   c) **1-CH_3**: Catalyst not soluble
   d) **1-OMe**: Catalyst not soluble
   e) **1-NMe_2**: Catalyst not soluble

2) After 30 minutes of heating
   a) **1-NO_2**: Consider amounts of insoluble material remains
   b) **1-Br**: Mostly dissolved, some fine insoluble material remains
   c) **1-CH_3**: Mostly dissolved, some fine insoluble material remains
   d) **1-OMe**: Fully dissolved
   e) **1-NMe_2**: Consider amounts of insoluble material remains

3) After 17 hr of heating
   a) **1-NO_2**: Fully dissolved
   b) **1-Br**: Fully dissolved
   c) **1-CH_3**: Fully dissolved
   d) **1-OMe**: Fully dissolved
   e) **1-NMe_2**: Mostly dissolved, some fine insoluble material remains

4) After cooling to RT
   a) **1-NO_2**: Heavy precipitation occurs
   b) **1-Br**: Mostly dissolved, some fine precipitate
   c) **1-CH_3**: Mostly dissolved, some fine precipitate
   d) **1-OMe**: Fully dissolved
   e) **1-NMe_2**: Moderate precipitation occurs

5) After addition of internal standard (with dilution) and filtration.
   a) **1-NO_2**: Gradual, heavy crystallization occurs
   b) **1-Br**: Gradual, heavy crystallization occurs
   c) **1-CH_3**: Gradual, heavy crystallization occurs
   d) **1-OMe**: Gradual, heavy crystallization occurs
   e) **1-NMe_2**: Gradual, heavy crystallization occurs

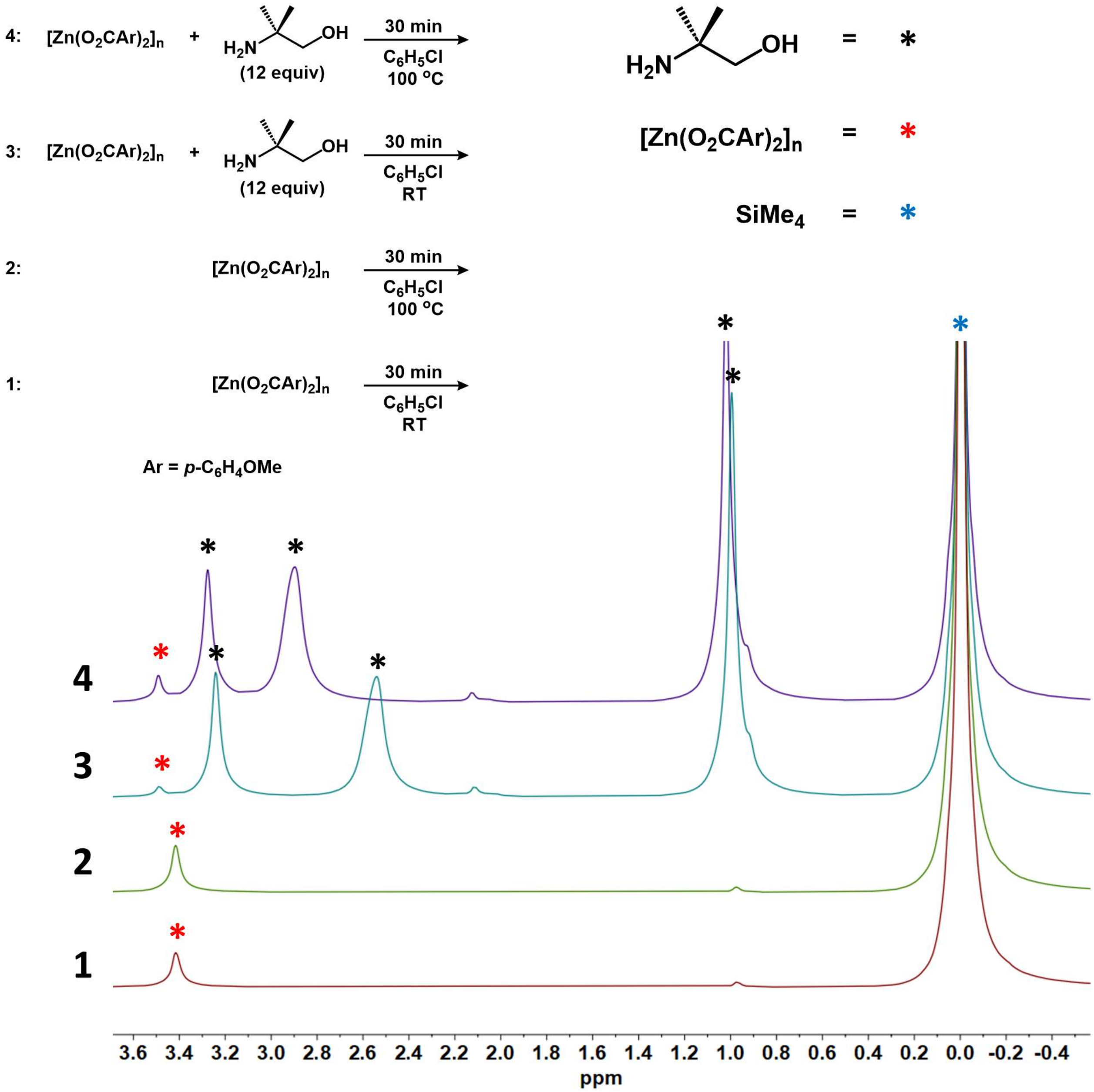

Figure S40. ^{1}H NMR (60 MHz, C_6H_5Cl) comparison of $[Zn(O_2CAr)_2]_n$ (Ar = *p*-C_6H_4OMe) with and without 2-amino-2-methyl-1-propanol (12 equiv) after 30 min at 100 °C or RT (approx. 18 °C).

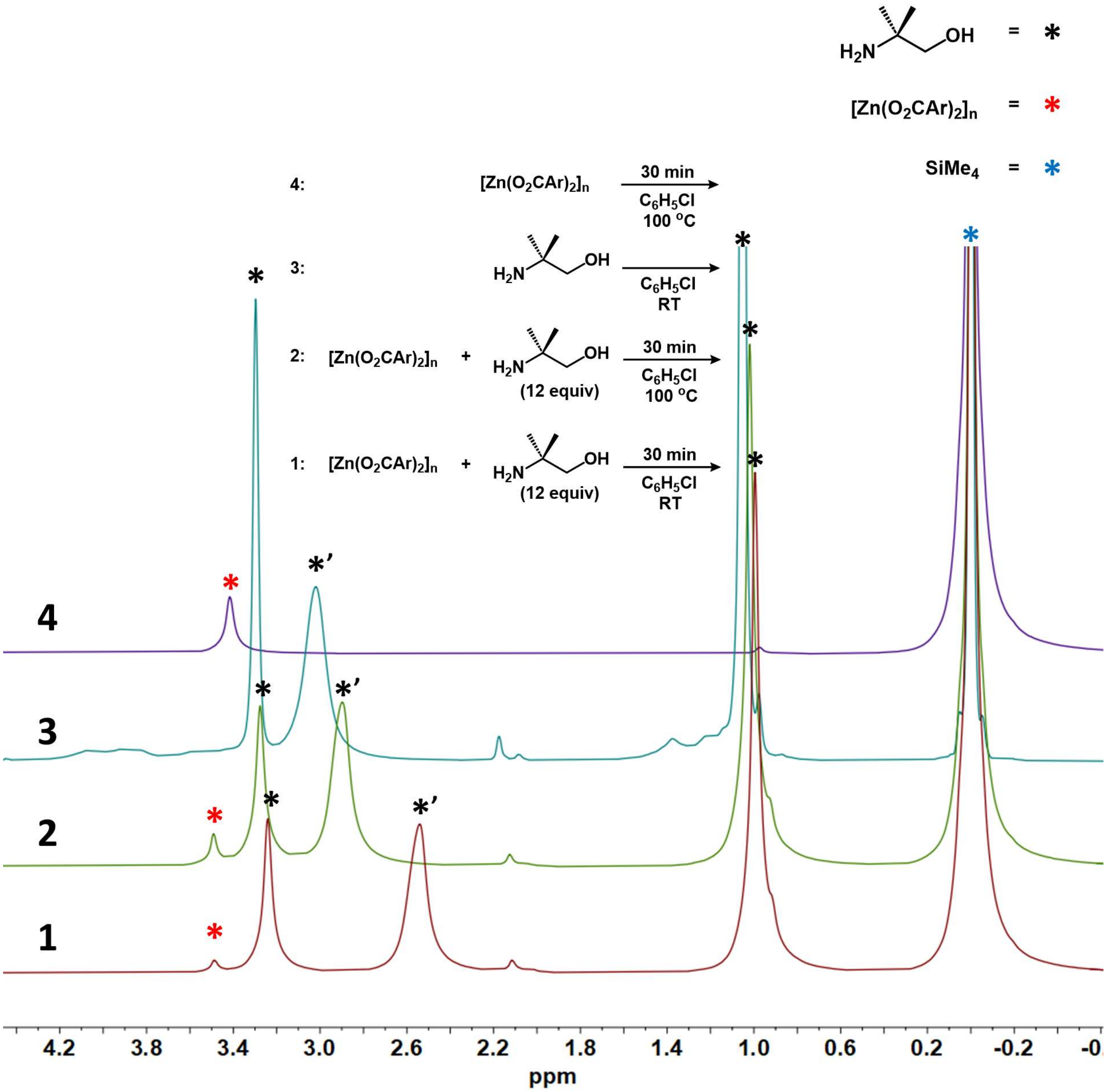

Figure S41. ^{1}H NMR (60 MHz, C_6H_5Cl) comparison of 2-amino-2-methyl-1-propanol (12 equiv) with and without $[Zn(O_2CAr)_2]_n$ (Ar = *p*-C_6H_4OMe) after 30 min at 100 °C or RT (approx. 18 °C).

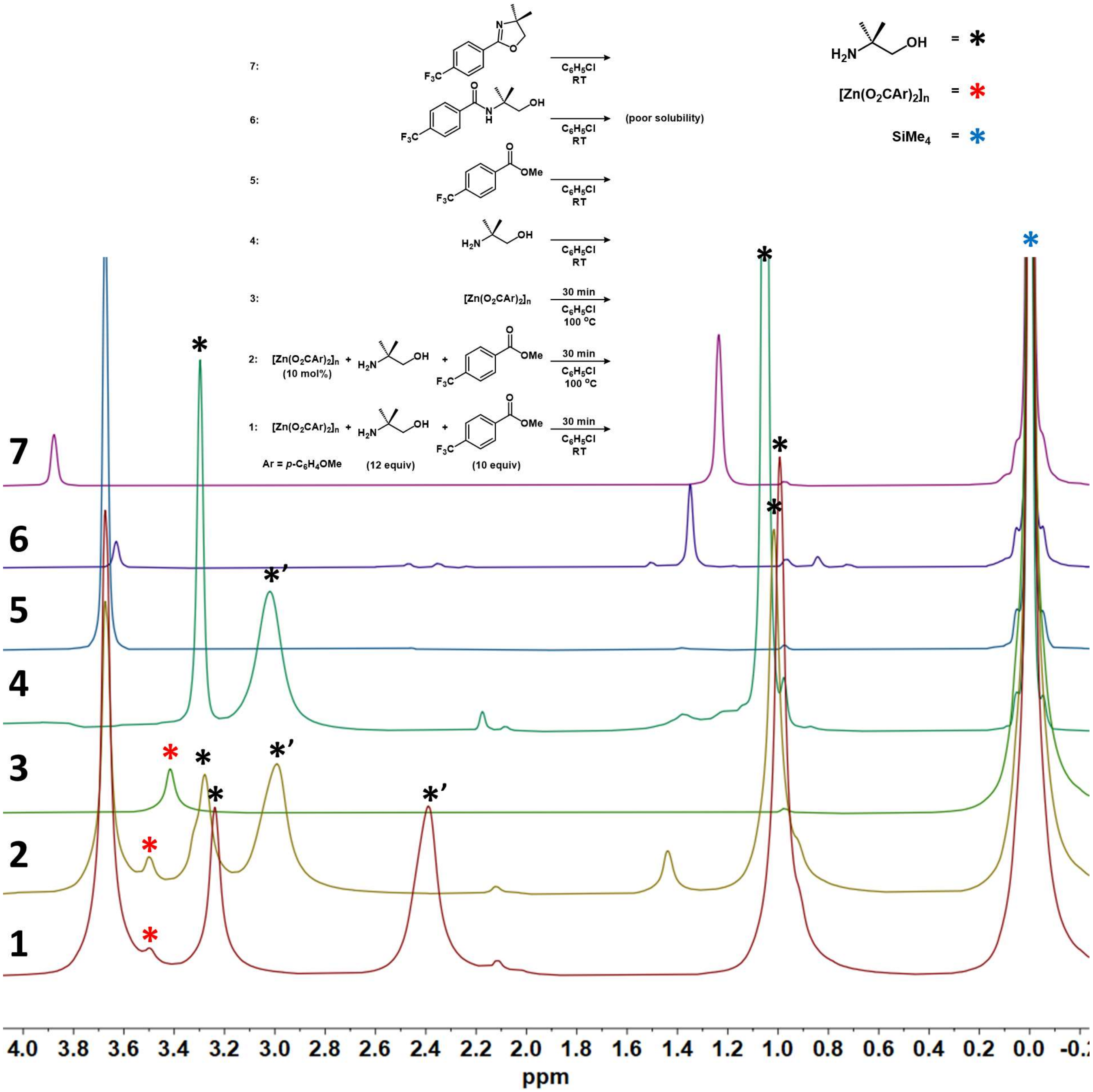

Figure S42. ^{1}H NMR (60 MHz, C_6H_5Cl) comparison of reaction progress of catalytic trial after 30 min at 100 °C or RT (approx. 18 °C). Standards of $[Zn(O_2CAr)_2]_n$ (Ar = *p*-C_6H_4OMe; 3), 2-amino-2-methyl-1-propanol (4), methyl 4-(trifluoromethyl)benzoate (5), [*N*-(2-hydroxy-1,1-dimethylethyl)-4-(trifluoromethyl)benzamide (6), and 4,4'-dimethyl-2-(4-trifluoromethyl)phenyl-4,5-dihydrooxazole (7) are included for comparison.

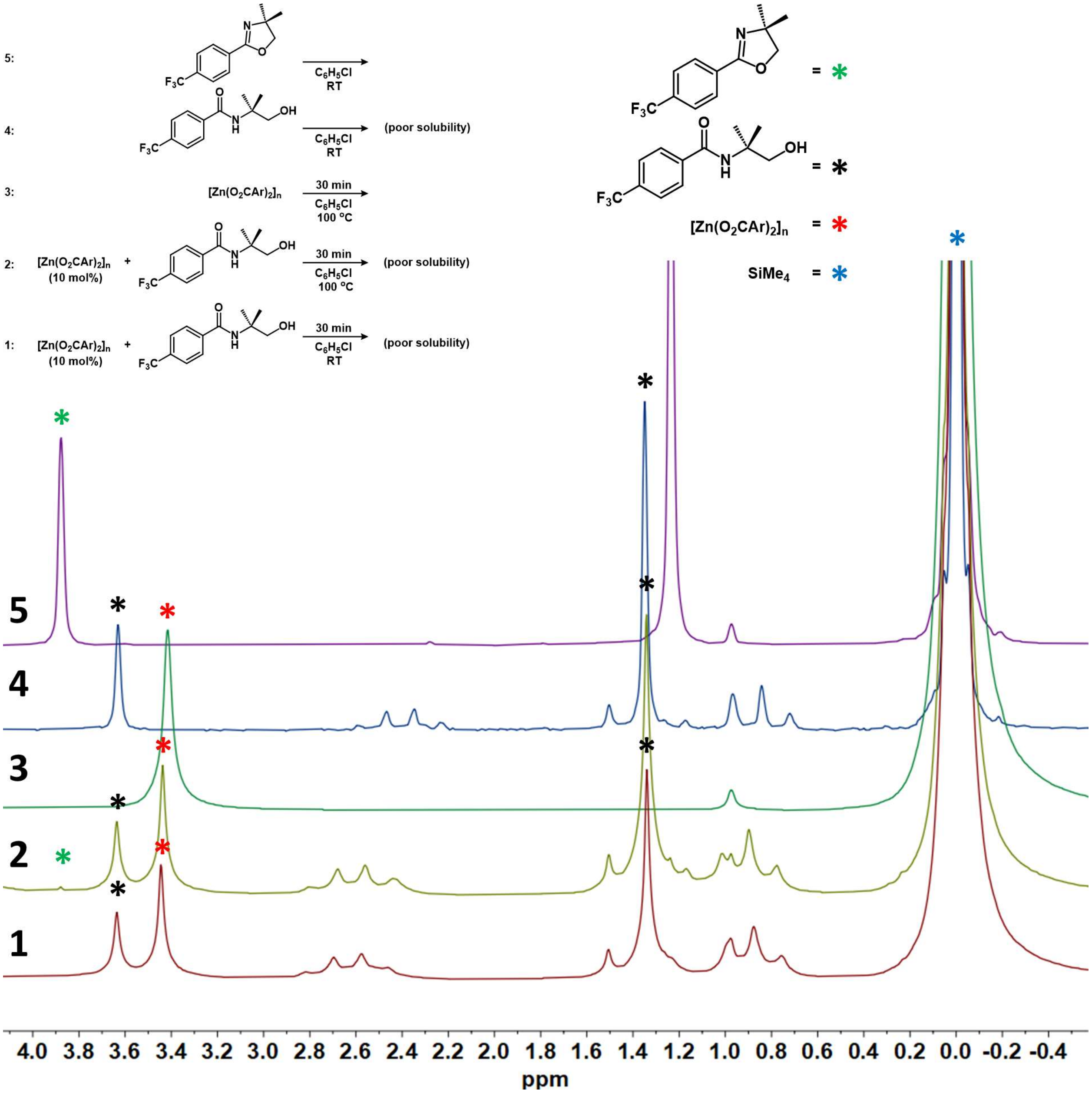

Figure S43. ^{1}H NMR (60 MHz, C_6H_5Cl) comparison of reaction progress of $[Zn(O_2CAr)_2]_n$ (Ar = *p*-C_6H_4OMe; 10 mol%) with [*N*-(2-hydroxy-1,1-dimethylethyl)-4-(trifluoromethyl)benzamide after 30 min at 100 °C or RT (approx. 18 °C). Standards of $[Zn(O_2CAr)_2]_n$ (Ar = *p*-C_6H_4OMe; 3), [*N*-(2-hydroxy-1,1-dimethylethyl)-4-(trifluoromethyl)benzamide (4), and 4,4'-dimethyl-2-(4-trifluoromethyl)phenyl-4,5-dihydrooxazole (5) are included for comparison. Authentic samples of [*N*-(2-hydroxy-1,1-dimethylethyl)-4-(trifluoromethyl)benzamide have minimal solubility in C_6H_5Cl. Upon heating to 100 °C (reaction 2), [*N*-(2-hydroxy-1,1-dimethylethyl)-4-(trifluoromethyl)benzamide fully dissolves and precipitates upon cooling.

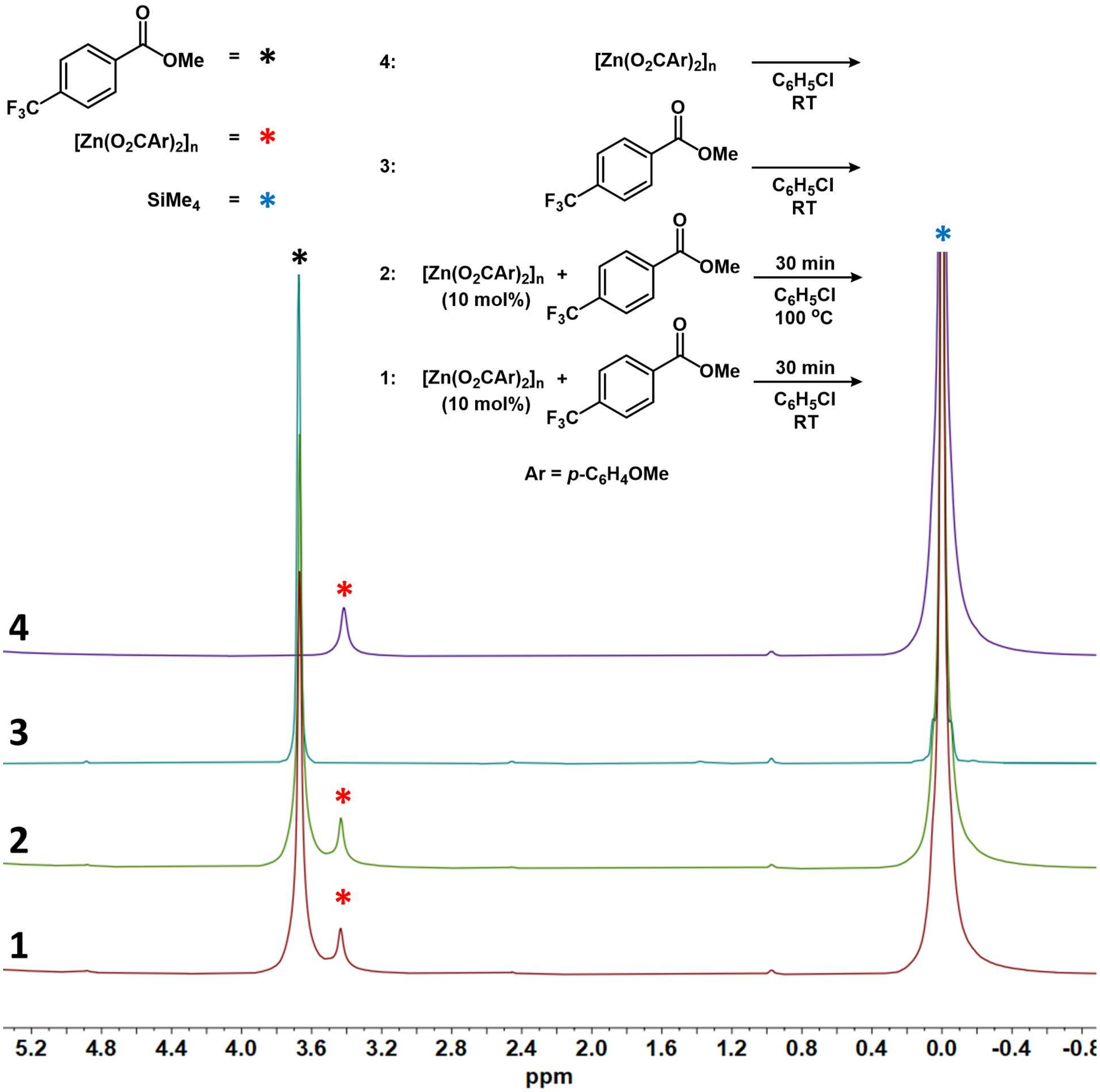

Figure S44. ^{1}H NMR (60 MHz, C_6H_5Cl) comparison of reaction between $[Zn(O_2CAr)_2]_n$ (Ar = *p*-C_6H_4OMe; 10 mol%) with methyl 4-(trifluoromethyl)benzoate after 30 min at 100 °C or RT (approx. 18 °C). Standards of methyl 4-(trifluoromethyl)benzoate (3) and $[Zn(O_2CAr)_2]_n$ (Ar = *p*-C_6H_4OMe; 4) included for reference. No reaction is observed.

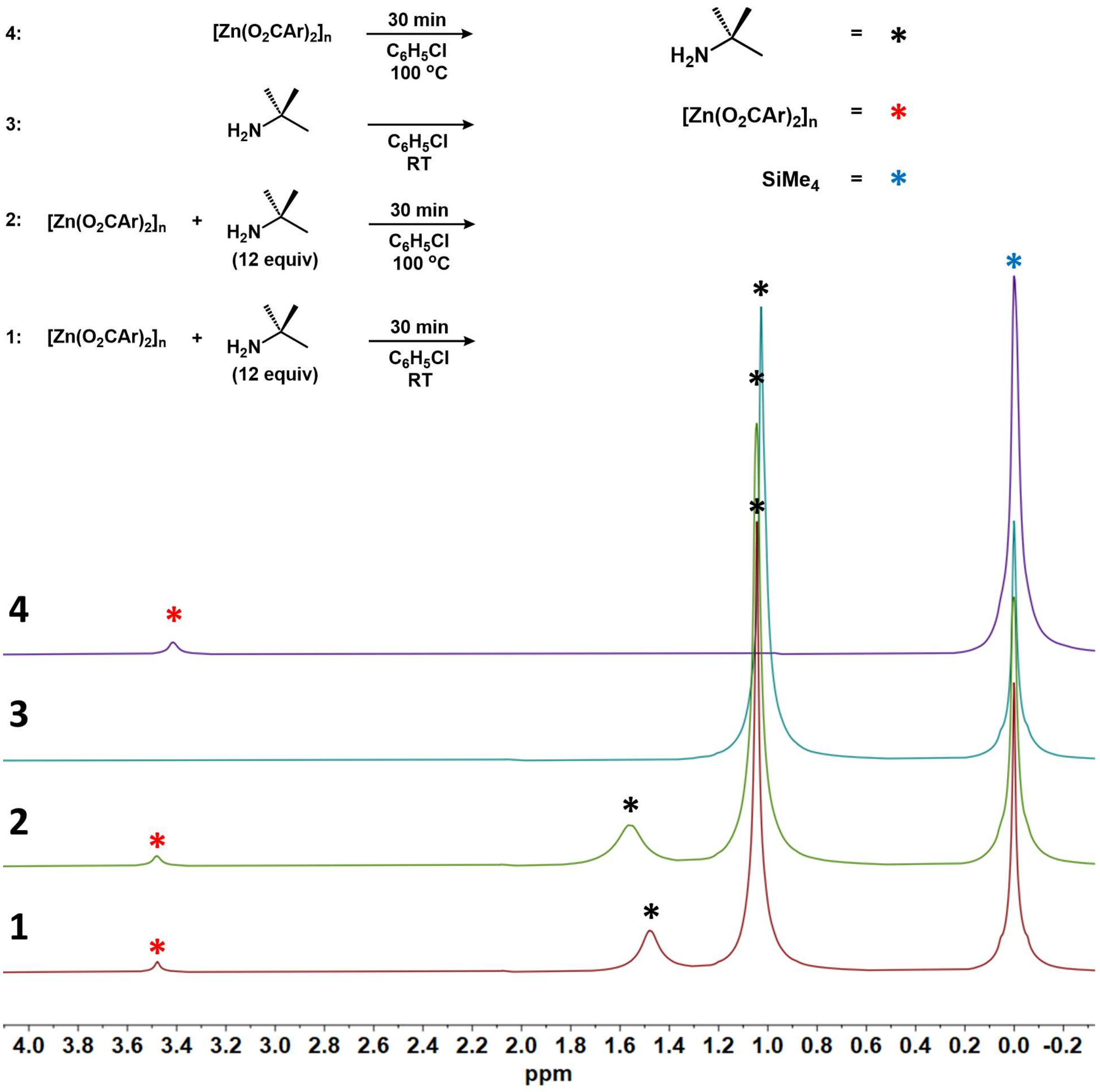

Figure S45. ^{1}H NMR (60 MHz, C_6H_5Cl) comparison of reaction between $[Zn(O_2CAr)_2]_n$ (Ar = *p*-C_6H_4OMe) and *tert*-butylamine (12 equiv) after 30 min at 100 °C or RT (approx. 18 °C). Standards *tert*-butylamine (3) and $[Zn(O_2CAr)_2]_n$ (Ar = *p*-C_6H_4OMe; 4) included for reference.

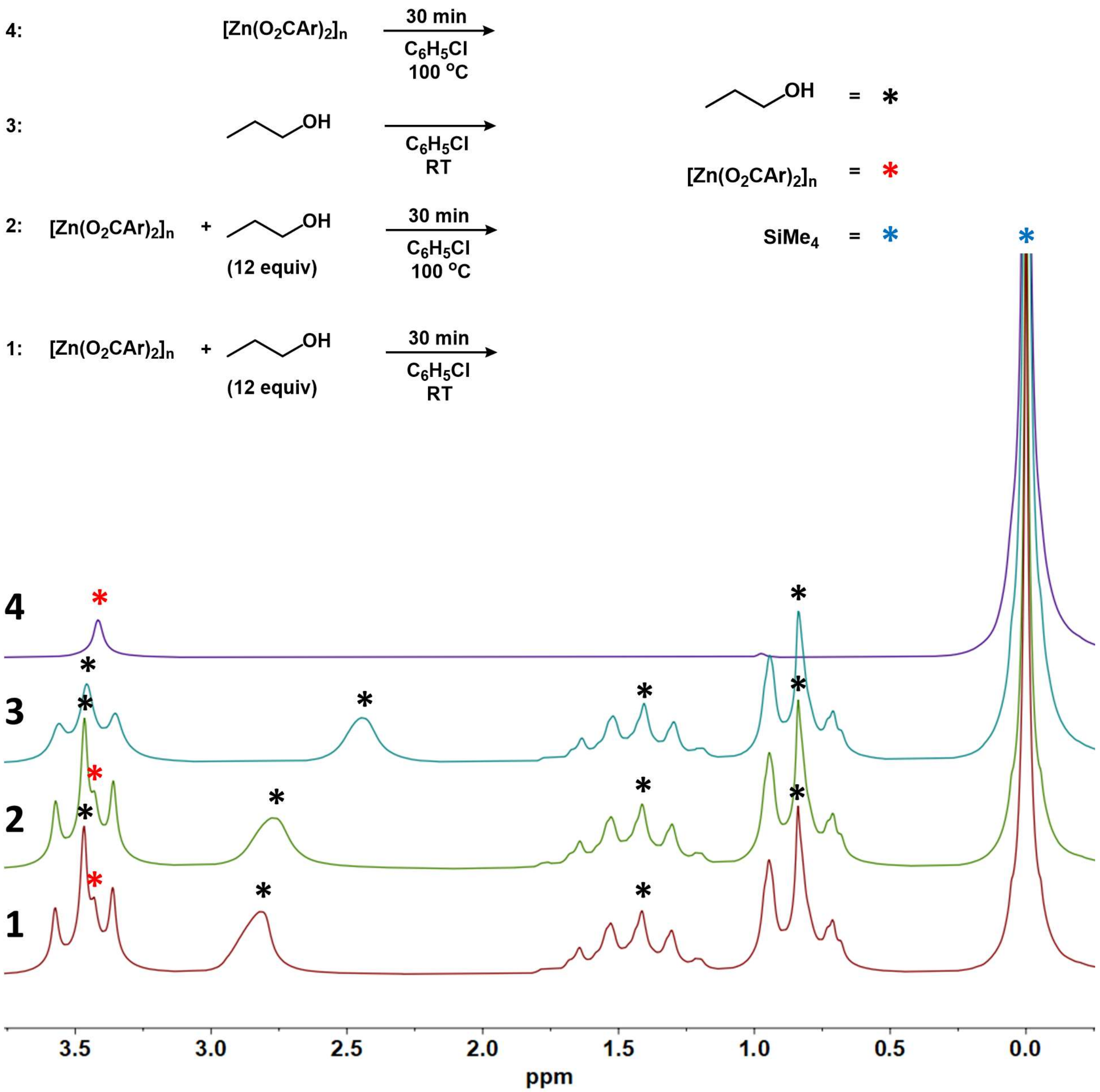

Figure S46. ^{1}H NMR (60 MHz, C_6H_5Cl) comparison of reaction between $[Zn(O_2CAr)_2]_n$ (Ar = *p*-C_6H_4OMe) and *n*-butanol (12 equiv) after 30 min at 100 °C or RT (approx. 18 °C). Standards *n*-butanol (3) and $[Zn(O_2CAr)_2]_n$ (Ar = *p*-C_6H_4OMe; 4) included for reference.

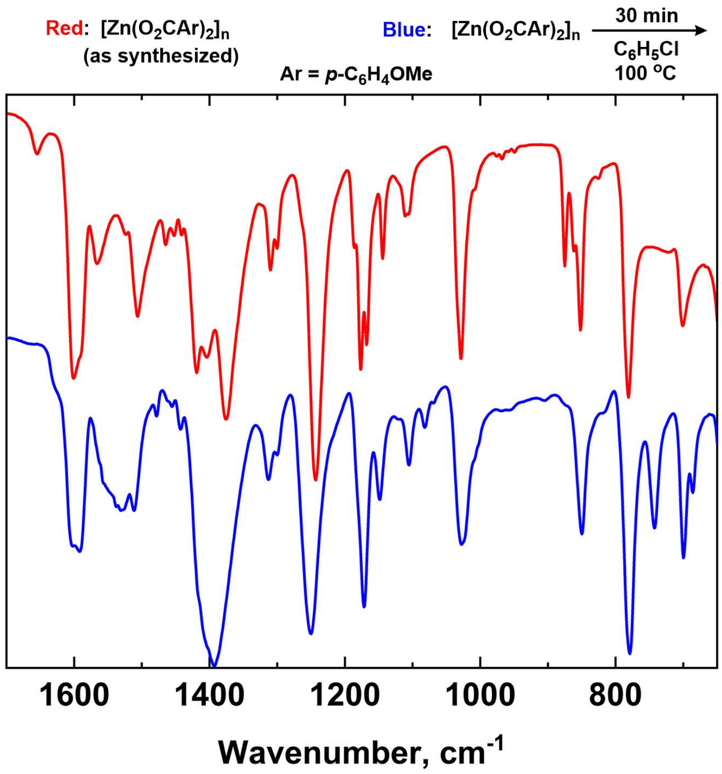

Figure S47. Infrared spectra (ATR, neat) comparison $[Zn(O_2CAr)_2]_n$ (Ar = *p*-C_6H_4OMe) as synthesized (red) and after heating to 100 °C in C_6H_5Cl for 30 min (blue; solvent removed in vacuo prior to analysis).

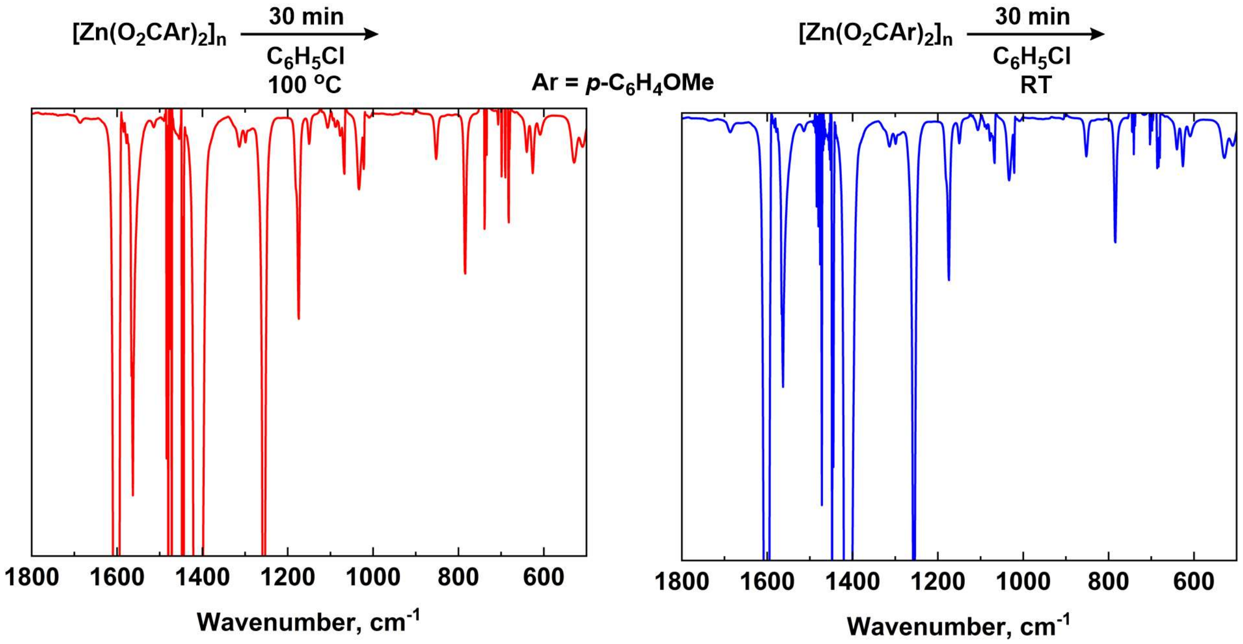

Figure S48. Infrared spectra (solution, C_6H_5Cl) comparison $[Zn(O_2CAr)_2]_n$ (Ar = *p*-C_6H_4OMe) after heating at 100 °C in C_6H_5Cl for 30 min (red, left) and after stirring at RT in C_6H_5Cl for 30 min (blue, right).

Compound: $Zn_4O(O_2CAr)_6$ (Ar = *p*-$C_6H_4OCH_3$)

Local Name: JK-1-24

CCDC Number: 2342377

Table S3. Crystallographic details for $Zn_4O(O_2CAr)_6$ (Ar = *p*-$C_6H_4OCH_3$).

**Crystal data**	
Chemical formula	$C_{48}H_{42}O_{19}Zn_4 \cdot 0.436(C_7H_8) \cdot 0.243(C_6H_{14}) \cdot 0.57(C_2H_4Cl_2)$
M_r	1301.98
Crystal system, space group	Monoclinic, $P2_1/n$
Temperature (K)	150
a, *b*, *c* (Å)	24.218 (12), 15.714 (5), 29.151 (7)
β (°)	94.497 (17)
V (Å^3)	11060 (7)
Z	8
Radiation type	Mo *K*α
μ (mm^{-1})	1.84
Crystal size (mm)	0.55 × 0.45 × 0.07
**Data collection**	
Diffractometer	Bruker AXS D8 Quest diffractometer with PhotonII charge-integrating pixel array detector (CPAD)
Absorption correction	Multi-scan *SADABS* 2016/2: Krause, L., Herbst-Irmer, R., Sheldrick G.M. & Stalke D., J. Appl. Cryst. 48 (2015) 3-10
T_{min}, T_{max}	0.185, 0.334
No. of measured, independent and observed [*I* > 2s(*I*)] reflections	122694, 23611, 14386
R_{int}	0.092
(sin θ/λ)$_{max}$ (Å^{-1})	0.641
**Refinement**	
$R[F^2 > 2\sigma(F^2)]$, $wR(F^2)$, S	0.080, 0.201, 1.05
No. of reflections	23611
No. of parameters	1744
No. of restraints	1526
H-atom treatment	H-atom parameters constrained
	$w = 1/[s^2(F_o^2) + (0.0177P)^2 + 79.441P]$ where $P = (F_o^2 + 2F_c^2)/3$
$\Delta\rho_{max}$, $\Delta\rho_{min}$ (e Å^{-3})	0.94, -0.93

Computer programs: Apex4 v2021.10-RC6 (Bruker, 2021), *SAINT* V8.40B (Bruker, 2020), *SHELXT* (Sheldrick, 2015), *SHELXL2018*/3 (Sheldrick, 2015, 2018), SHELXLE Rev1275 (Hübschle *et al.*, 2011).

Refinement details:

Two anisyl groups are disordered by rotation of their methoxy groups. The disorder extends to the phenyl ring. The disorder for one of the anisyl groups is correlated with solvent disorder with one of the disordered moieties being present in the presence of a hexane molecule, and the other being present when this hexane is absent (and a further removed toluene or 1,2-dichloroethane molecule being present instead).

A 1,2-dichloroethane molecule is disordered by inversion. The two disordered moieties and that disordered with the hexane were restrained to have similar geometries.

A toluene molecule is disordered across an inversion center. Its phenyl ring and that of the toluene molecules disordered with the hexane were constrained to resemble an ideal hexagon with C-C bond distances of 1.39 Å. The C-C(methyl) distances were restrained to a target value of 1.55(2) Å. The toluene molecules disordered with the hexane were also restrained to be close to planar, and the C-C-C angles involving the methyl-C atom were restrained to be similar.

All disordered anisyl groups were restrained to have similar geometries as another not disordered anisyl group. The C-C bond distances and angles of the hexane molecule were restrained to target values.

U^{ij} components of ADPs for disordered atoms closer to each other than 2.0 Å were restrained to be similar.

Subject to these conditions, the occupancy ratios for the two anisyl groups refined to 0.724(9) to 0.724(9) for the independently disordered anisyl (involving O13) and to 0.487(3) to 0.513(3) for the anisyl disordered with the hexane molecule (involving O38). The hexane molecule shares the 0.487(3) occupancy. The occupancies of the two toluene and the 1,2 dichloroethane molecules disordered with the hexane refined to 0.168(3), 0.205(3) and 0.140(3), respectively. The occupancy ratio for the 1,2 dichloroethane molecule refined to 0.659(13) to 0.341(13).

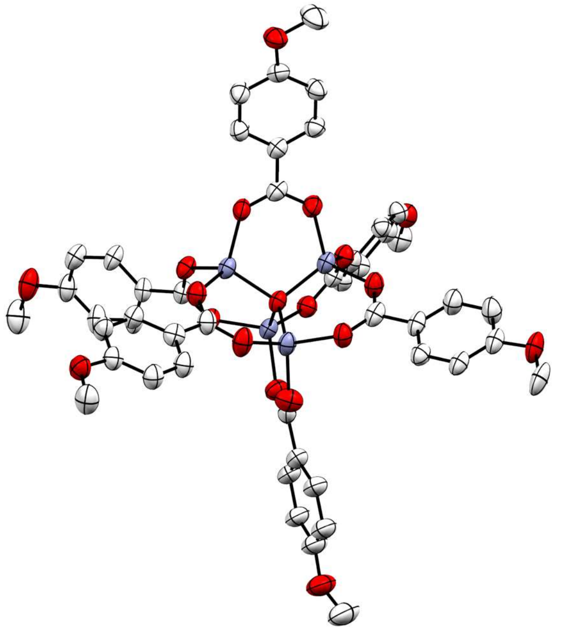

Figure S49. Molecular structure of $Zn_4O(O_2CAr)_6$ (Ar = *p*-$C_6H_4OCH_3$) displayed with 50% probability ellipsoids. Hydrogen atoms, solvent molecules, and disordered moieties are omitted for clarity.

Compound: $Zn_4O(O_2CAr)_6(dmso)_2$ (Ar = *p*-$C_6H_4NMe_2$)

Local Name: JK-1-25

CCDC Number: 2342376

Table S4. Crystallographic details for $Zn_4O(O_2CAr)_6(dmso)_2$ (Ar = *p*-$C_6H_4NMe_2$).

**Crystal data**	
Chemical formula	$C_{58}H_{72}N_6O_{15}S_2Zn_4 \cdot 0.141(C_2H_6OS)$
M_r	1429.93
Crystal system, space group	Trigonal, $R\bar{3}$:*H*
Temperature (K)	150
a, *c* (Å)	56.5971 (9), 10.5189 (2)
V (Å^3)	29180.3 (11)
Z	18
Radiation type	Cu $K\alpha$
μ (mm^{-1})	2.88
Crystal size (mm)	0.22 × 0.05 × 0.04
**Data collection**	
Diffractometer	Bruker AXS D8 Quest diffractometer with PhotonIII_C14 charge-integrating and photon counting pixel array detector
Absorption correction	Multi-scan *SADABS* 2016/2: Krause, L., Herbst-Irmer, R., Sheldrick G.M. & Stalke D., J. Appl. Cryst. 48 (2015) 3-10
T_{min}, T_{max}	0.639, 0.754
No. of measured, independent and observed [$I > 2s(I)$] reflections	53747, 13096, 9113
R_{int}	0.041
$(\sin \theta/\lambda)_{max}$ (Å^{-1})	0.638
**Refinement**	
$R[F^2 > 2\sigma(F^2)]$, $wR(F^2)$, *S*	0.054, 0.135, 1.01
No. of reflections	13096
No. of parameters	1301
No. of restraints	2683
H-atom treatment	H-atom parameters constrained
	$w = 1/[s^2(F_o^2) + (0.0433P)^2 + 146.3402P]$ where $P = (F_o^2 + 2F_c^2)/3$
$\Delta\rho_{max}$, $\Delta\rho_{min}$ (e Å^{-3})	0.75, -0.94

Computer programs: Apex4 v2021.10-RC6 (Bruker, 2021), *SAINT* V8.40B (Bruker, 2020), *SHELXT* (Sheldrick, 2015), *SHELXL2018*/3 (Sheldrick, 2015, 2018), SHELXLE Rev1275 (Hübschle *et al.*, 2011).

Refinement details:

Extensive disorder is observed for the dimethylamino benzoate ligands. Ligand 3 is disordered over two orientations. The major moiety is also incompatible with the minor moiety of the same ligand in a neighboring molecule. Ligands 4 and 6 are disordered in general positions over each two moieties. Ligand 2 was refined as disordered over two moieties, with the major moiety being in conflict with a partially occupied solvate DMSO molecule that is disordered around a three-fold rotinversion axis. Ligands 1 and 5 were refined as not disordered.

Disorder is also observed for one of two metal-coordinated DMSO molecules. All dimethylamino benzoate ligands and all DMSO molecules were each restrained to have similar geometries. U^{ij} components of ADPs for disordered atoms closer to each other than 2.0 Å were restrained to be similar. The carboxylate sections and the dimethylamino benzene sections of ligands 4 and minor ligand 3 were restrained to be close to planar. Subject to these conditions, the occupancy ratios refined to 0.717(4) to 0.283(4) for ligand 2, to 0.726(4) to 0.274(4) for ligand 3, to 0.492(9) to 0.508(9) for ligand 4, to 0.635(9) to 0.365(9) for ligand 6, and to 0.936(3) to 0.064(3) for the DMSO of S1. The occupancy rate for the six-fold disordered DMSO (of S3) refined to 0.1413(18).

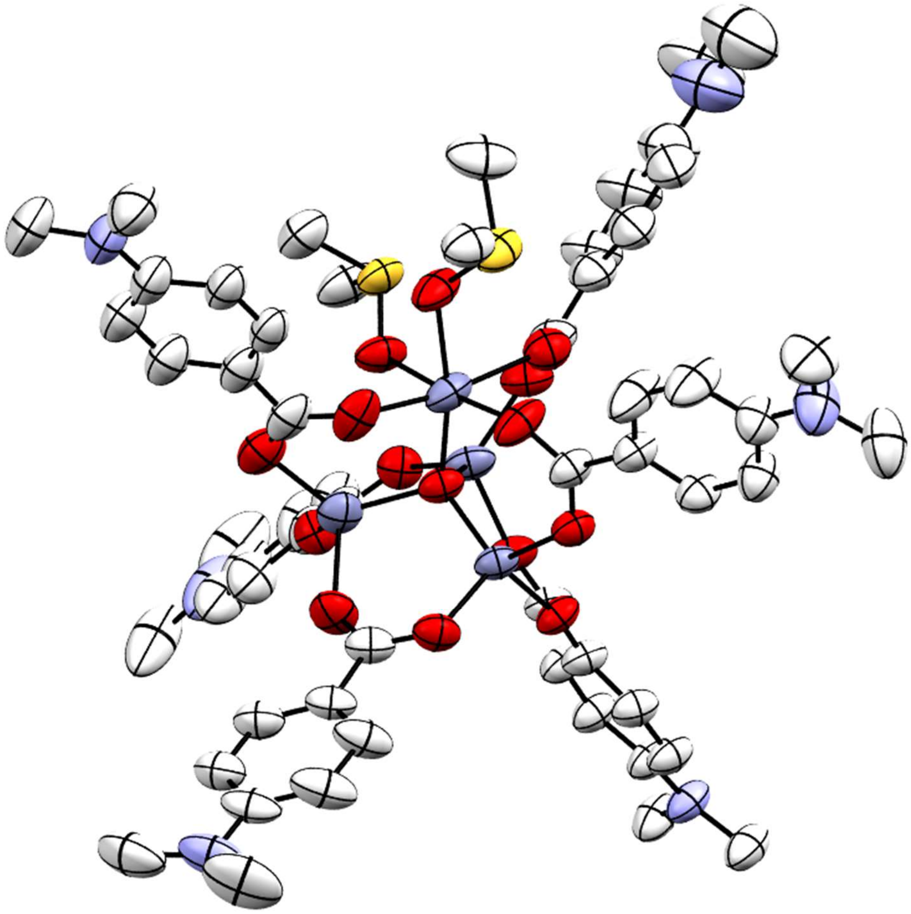

Figure S50. Molecular structure of $Zn_4O(O_2CAr)_6(dmso)_2$ (Ar = *p*-$C_6H_4NMe_2$) displayed with 50% probability ellipsoids. Hydrogen atoms and disordered moieties are omitted for clarity.

Compound: $Zn(O_2CAr)_2(dmf)_2$ (Ar = *p*-$C_6H_4NO_2$)

Local Name: JK-1-21

CCDC Number: 2342381

Table S5. Crystallographic details for $Zn(O_2CAr)_2(dmf)_2$ (Ar = *p*-$C_6H_4NO_2$).

**Crystal data**	
Chemical formula	$C_{20}H_{22}N_4O_{10}Zn$
M_r	543.78
Crystal system, space group	Monoclinic, *C*2/*c*
Temperature (K)	150
a, *b*, *c* (Å)	27.4286 (13), 6.1324 (2), 15.4135 (8)
β (°)	118.710 (2)
V (Å^3)	2273.87 (18)
Z	4
Radiation type	Mo $K\alpha$
μ (mm^{-1})	1.15
Crystal size (mm)	0.32 × 0.18 × 0.11
**Data collection**	
Diffractometer	Bruker AXS D8 Quest
Absorption correction	Multi-scan *SADABS* 2016/2: Krause, L., Herbst-Irmer, R., Sheldrick G.M. & Stalke D., J. Appl. Cryst. 48 (2015) 3-10
T_{min}, T_{max}	0.598, 0.747
No. of measured, independent and observed [$I > 2s(I)$] reflections	25343, 4337, 3693
R_{int}	0.039
$(\sin \theta/\lambda)_{max}$ (Å^{-1})	0.770
**Refinement**	
$R[F^2 > 2\sigma(F^2)]$, $wR(F^2)$, S	0.027, 0.076, 1.09
No. of reflections	4337
No. of parameters	161
H-atom treatment	H-atom parameters constrained
$\Delta\rho_{max}$, $\Delta\rho_{min}$ (e Å^{-3})	0.43, -0.31

Computer programs: Apex4 v2022.1-1 (Bruker, 2022), *SAINT* V8.40B (Bruker, 2020), *SHELXT* (Sheldrick, 2015b), *SHELXL2018*/3 (Sheldrick, 2015a, 2018), SHELXLE Rev1385 (Hübschle *et al.*, 2011).

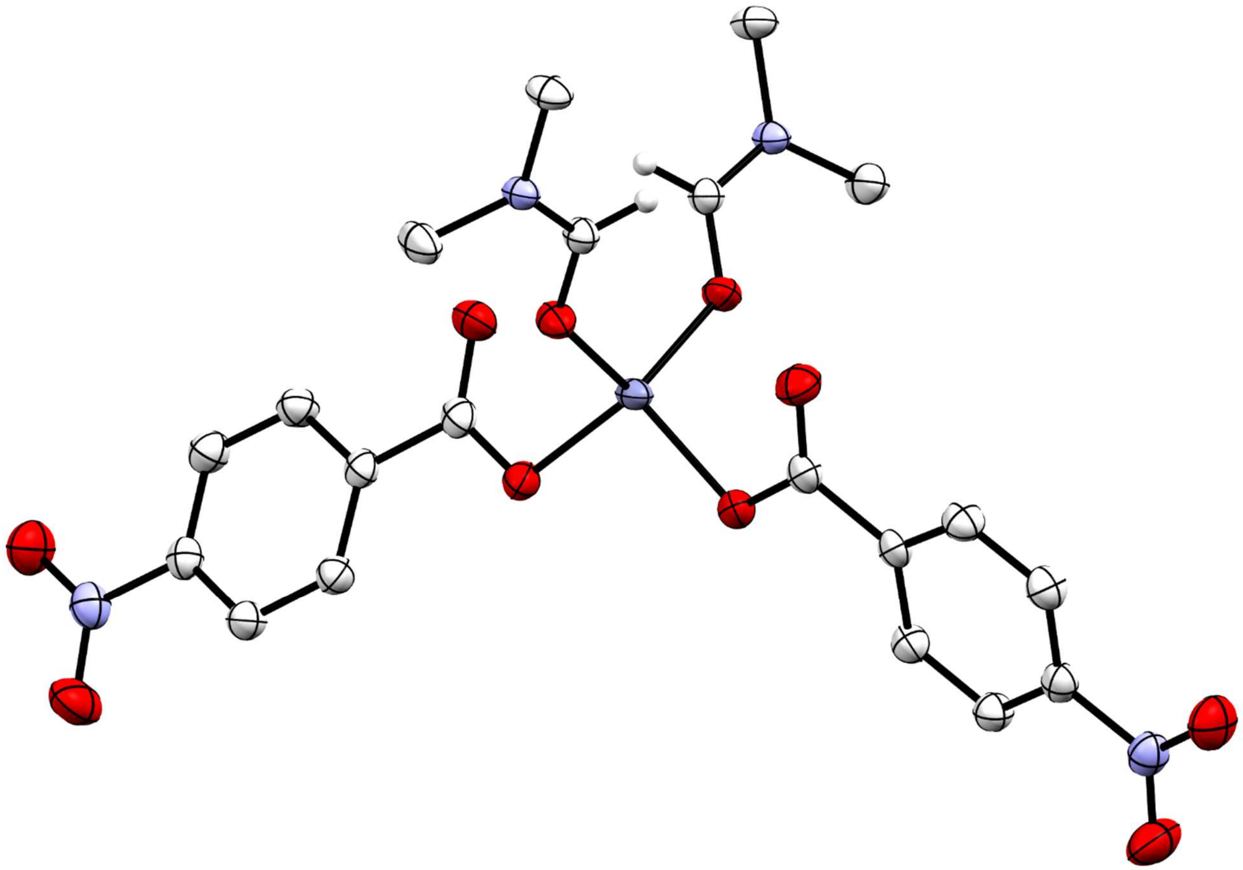

Figure S51. Molecular structure of $Zn(O_2CAr)_2(dmf)_2$ (Ar = *p*-$C_6H_4NO_2$) displayed with 50% probability ellipsoids. All hydrogen atoms except those attached to the N,N-dimethylformamide C=O are omitted for clarity.

Compound: (tmeda)Zn(O_2CAr)$_2$ (Ar = *p*-C_6H_4OMe)

Local Name: JK-1-44

CCDC Number: 2342374

Table S6. Crystallographic details for (tmeda)Zn(O_2CAr)$_2$ (Ar = *p*-C_6H_4OMe).

**Crystal data**	
Chemical formula	$C_{22}H_{30}N_2O_6Zn$
M_r	483.85
Crystal system, space group	Orthorhombic, *Pbca*
Temperature (K)	150
a, *b*, *c* (Å)	7.3016 (3), 21.6604 (12), 29.1943 (13)
V (Å^3)	4617.2 (4)
Z	8
Radiation type	Mo *K*α
μ (mm^{-1})	1.10
Crystal size (mm)	0.26 × 0.25 × 0.11
**Data collection**	
Diffractometer	Bruker AXS D8 Quest
Absorption correction	Multi-scan *SADABS* 2016/2: Krause, L., Herbst-Irmer, R., Sheldrick G.M. & Stalke D., J. Appl. Cryst. 48 (2015) 3-10
T_{min}, T_{max}	0.591, 0.747
No. of measured, independent and observed [I > 2s(I)] reflections	41930, 8809, 7050
R_{int}	0.028
(sin θ/λ)$_{max}$ (Å^{-1})	0.770
**Refinement**	
$R[F^2 > 2\sigma(F^2)]$, $wR(F^2)$, S	0.027, 0.073, 1.04
No. of reflections	8809
No. of parameters	286
H-atom treatment	H-atom parameters constrained
Δρ$_{max}$, Δρ$_{min}$ (e Å^{-3})	0.38, -0.39

Computer programs: Apex4 v2022.1-1 (Bruker, 2022), *SAINT* V8.40B (Bruker, 2020), *SHELXT* (Sheldrick, 2015b), *SHELXL2018*/3 (Sheldrick, 2015a, 2018), SHELXLE Rev1385 (Hübschle *et al.*, 2011).

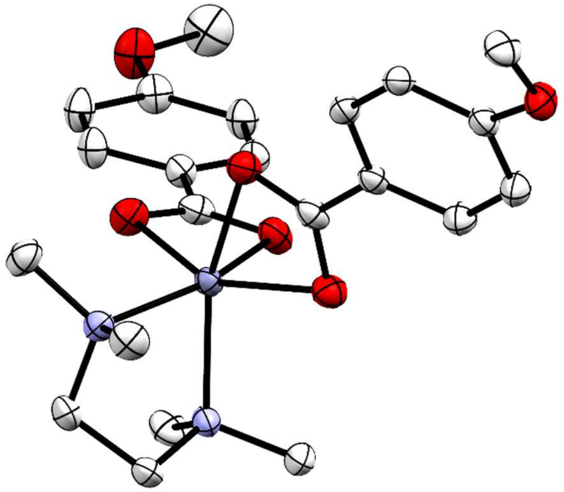

Figure 52. Molecular structure of (tmeda)Zn(O_2CAr)$_2$ (Ar = *p*-C_6H_4OMe) displayed with 50% probability ellipsoids. Hydrogen atoms are omitted for clarity.

Compound: (tmeda)Zn(O_2CAr)$_2$ (Ar = *p*-$C_6H_4NO_2$)

Local Name: JK-1-49

CCDC Number: 2342373

Table S7. Crystallographic details for (tmeda)Zn(O_2CAr)$_2$ (Ar = *p*-$C_6H_4NO_2$)

**Crystal data**	
Chemical formula	$C_{20}H_{24}N_4O_8Zn$
M_r	513.80
Crystal system, space group	Monoclinic, *P*2/*c*
Temperature (K)	150
a, *b*, *c* (Å)	7.1251 (3), 7.4464 (3), 21.1600 (9)
β (°)	92.1050 (16)
V (Å^3)	1121.91 (8)
Z	2
Radiation type	Mo $K\alpha$
μ (mm^{-1})	1.15
Crystal size (mm)	0.19 × 0.12 × 0.11
**Data collection**	
Diffractometer	Bruker AXS D8 Quest
Absorption correction	Multi-scan *SADABS* 2016/2: Krause, L., Herbst-Irmer, R., Sheldrick G.M. & Stalke D., J. Appl. Cryst. 48 (2015) 3-10
T_{min}, T_{max}	0.636, 0.747
No. of measured, independent and observed [I > 2s(I)] reflections	16487, 4237, 3459
R_{int}	0.035
(sin θ/λ)$_{max}$ (Å^{-1})	0.770
**Refinement**	
$R[F^2 > 2\sigma(F^2)]$, $wR(F^2)$, S	0.032, 0.075, 1.03
No. of reflections	4237
No. of parameters	152
H-atom treatment	H-atom parameters constrained
$\Delta\rho_{max}$, $\Delta\rho_{min}$ (e Å^{-3})	0.41, -0.34

Computer programs: Apex4 v2022.1-1 (Bruker, 2022), *SAINT* V8.40B (Bruker, 2020), *SHELXT* (Sheldrick, 2015b), *SHELXL2018*/3 (Sheldrick, 2015a, 2018), SHELXLE Rev1460 (Hübschle *et al.*, 2011).

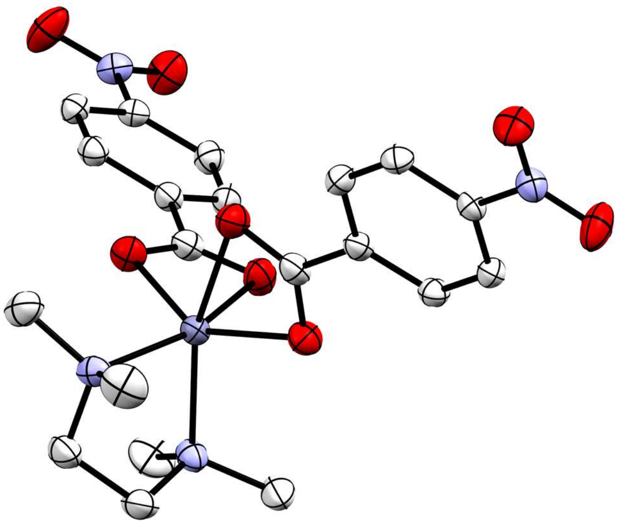

Figure S53. Molecular structure of (tmeda)Zn(O_2CAr)$_2$ (Ar = *p*-$C_6H_4NO_2$) displayed with 50% probability ellipsoids. Hydrogen atoms are omitted for clarity.

Compound: $(tpy^R)Zn(O_2CAr)_2$ (Ar = *p*-C_6H_4OMe; tpy^R = 4'-(4-methylphenyl)-2,2':6',2''-terpyridine)

Local Name: JK-1-42

CCDC Number: 2342378

Table S8. Crystallographic details for $(tpy^R)Zn(O_2CAr)_2$ (Ar = *p*-C_6H_4OMe; tpy^R = 4'-(4-methylphenyl)-2,2':6',2''-terpyridine).

**Crystal data**	
Chemical formula	$3(C_{38}H_{31}N_3O_6Zn)\cdot 2(C_6H_{14})\cdot$[+solvent]
M_r	2245.49
Crystal system, space group	Monoclinic, *C*2/*c*
Temperature (K)	150
a, *b*, *c* (Å)	24.7343 (9), 34.2661 (11), 17.7307 (6)
β (°)	124.536 (2)
V ($Å^3$)	12379.3 (8)
Z	4
Radiation type	Cu $K\alpha$
μ (mm^{-1})	1.21
Crystal size (mm)	0.21 × 0.20 × 0.20
**Data collection**	
Diffractometer	Bruker AXS D8 Quest
Absorption correction	Multi-scan *SADABS* 2016/2: Krause, L., Herbst-Irmer, R., Sheldrick G.M. & Stalke D., J. Appl. Cryst. 48 (2015) 3-10
T_{min}, T_{max}	0.677, 0.754
No. of measured, independent and observed [$I > 2s(I)$] reflections	65384, 12963, 12272
R_{int}	0.033
$(\sin \theta/\lambda)_{max}$ ($Å^{-1}$)	0.638
**Refinement**	
$R[F^2 > 2\sigma(F^2)]$, $wR(F^2)$, S	0.032, 0.091, 1.03
No. of reflections	12963
No. of parameters	896
No. of restraints	706
H-atom treatment	H-atom parameters constrained
$\Delta\rho_{max}$, $\Delta\rho_{min}$ (e $Å^{-3}$)	0.38, -0.44

Computer programs: Apex4 v2022.1-1 (Bruker, 2022), *SAINT* V8.40B (Bruker, 2020), *SHELXT* (Sheldrick, 2015b), *SHELXL2018*/3 (Sheldrick, 2015a, 2018), SHELXLE Rev1385 (Hübschle *et al.*, 2011).

Refinement details:

An anisole ligand was refined as disordered. The two disordered moieties were restrained to have similar geometries. U^{ij} components of ADPs for disordered atoms closer to each other than 2.0 Å were restrained to be similar. Subject to these conditions, the occupancy ratio refined to 0.73(2) to 0.27(2).

A hexane solvate molecule was refined as three-fold disordered. The disordered moieties were restrained to have similar geometries. Bond distances and angles were restrained to target values. U^{ij} components of ADPs for disordered atoms closer to each other than 2.0 Å were restrained to be similar. Subject to these conditions, the occupancy ratio refined to 0.429(3) to 0.290(3) to 0.281(3).

The structure contains additional 1519 $Å^3$ of solvent accessible voids. The residual electron density peaks are not arranged in an interpretable pattern. The structure factors were instead augmented via reverse Fourier transform methods using the SQUEEZE routine (P. van der Sluis & A.L. Spek (1990). Acta Cryst. A46, 194-201) as implemented in the program Platon. The resultant FAB file containing the structure factor contribution from the electron content of the void space was used along with the original hkl file in the further refinement. (The FAB file with details of the Squeeze results is appended to the cif file). The Squeeze procedure corrected for 314 electrons within the solvent accessible voids.

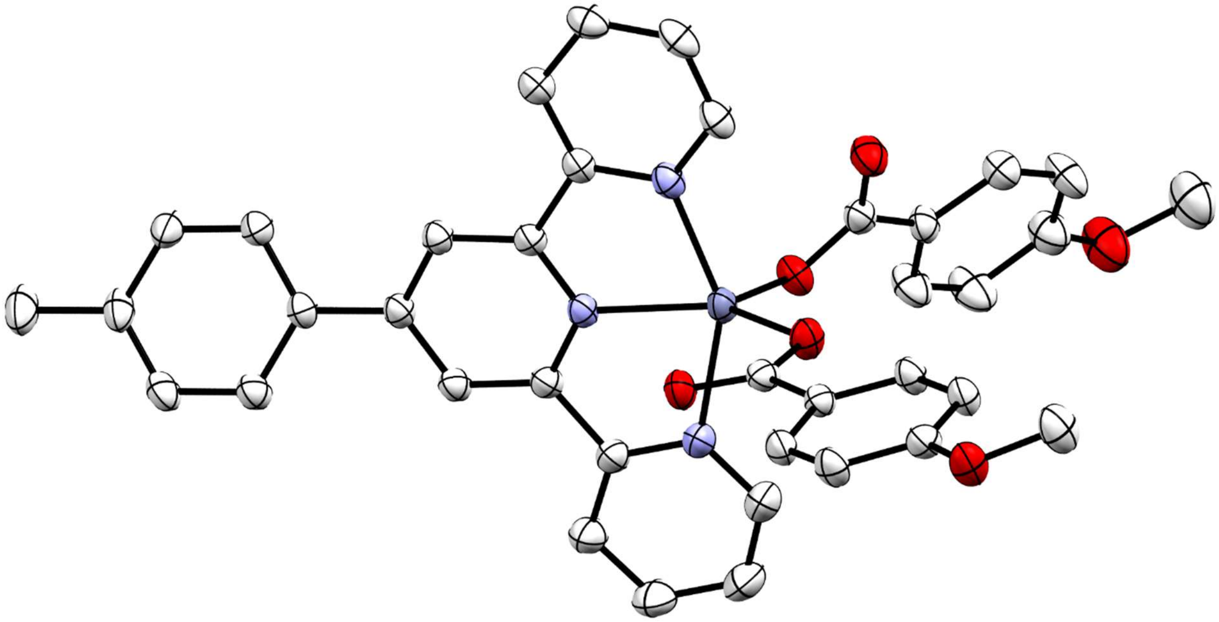

Figure S54. Molecular structure of $(tpy^R)Zn(O_2CAr)_2$ (Ar = *p*-C_6H_4OMe; tpy^R = 4'-(4-methylphenyl)-2,2':6',2''-terpyridine) displayed with 50% probability ellipsoids. Hydrogen atoms, disordered moieties, and solvent molecules are omitted for clarity.

Compound: (phen)Zn(O_2CAr)$_2$(H_2O) (Ar = *p*-C_6H_4OMe)

Local Name: JK-1-43a

CCDC Number: 2342375

Table S9. Crystallographic details for (phen)Zn(O_2CAr)$_2$(H_2O) (Ar = *p*-C_6H_4OMe).

**Crystal data**	
Chemical formula	$C_{28}H_{24}N_2O_7Zn$
M_r	565.86
Crystal system, space group	Monoclinic, $P2_1/c$
Temperature (K)	150
a, *b*, *c* (Å)	13.0707 (5), 15.1521 (4), 12.8504 (5)
β (°)	105.560 (1)
V (Å^3)	2451.73 (15)
Z	4
Radiation type	Mo $K\alpha$
μ (mm^{-1})	1.06
Crystal size (mm)	0.21 × 0.20 × 0.09
**Data collection**	
Diffractometer	Bruker AXS D8 Quest
Absorption correction	Multi-scan *SADABS* 2016/2: Krause, L., Herbst-Irmer, R., Sheldrick G.M. & Stalke D., J. Appl. Cryst. 48 (2015) 3-10
T_{min}, T_{max}	0.642, 0.747
No. of measured, independent and observed [$I > 2s(I)$] reflections	43308, 9373, 7620
R_{int}	0.042
(sin θ/λ)$_{max}$ (Å^{-1})	0.771
**Refinement**	
$R[F^2 > 2\sigma(F^2)]$, $wR(F^2)$, S	0.031, 0.083, 1.02
No. of reflections	9373
No. of parameters	351
H-atom treatment	H atoms treated by a mixture of independent and constrained refinement
$\Delta\rho_{max}$, $\Delta\rho_{min}$ (e Å^{-3})	0.51, -0.35

Computer programs: Apex4 v2022.1-1 (Bruker, 2022), *SAINT* V8.40B (Bruker, 2020), *SHELXT* (Sheldrick, 2015b), *SHELXL2018*/3 (Sheldrick, 2015a, 2018), SHELXLE Rev1385 (Hübschle *et al.*, 2011).

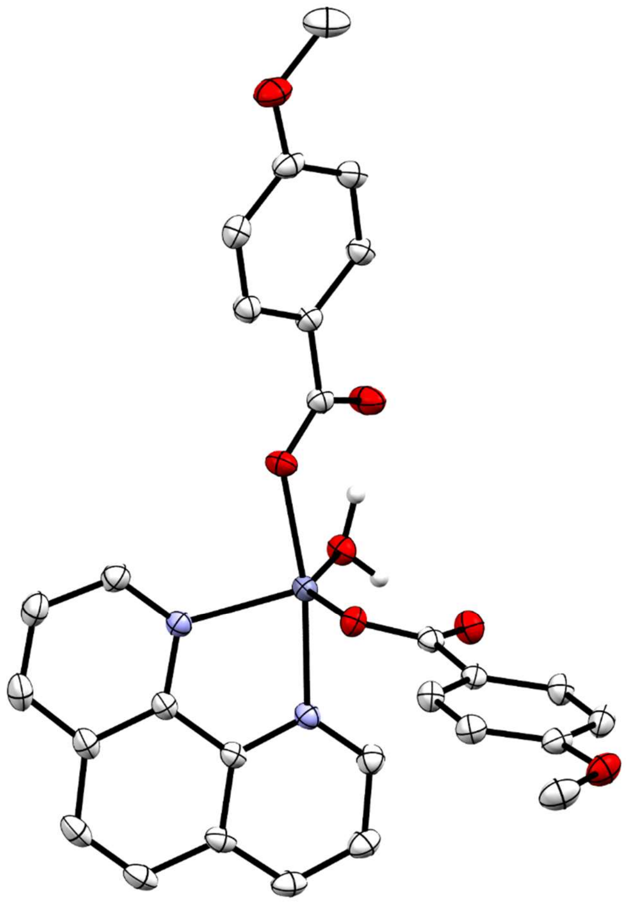

Figure S55. Molecular structure of (phen)Zn(O_2CAr)$_2$(H_2O) (Ar = *p*-C_6H_4OMe) displayed with 50% probability ellipsoids. All hydrogen atoms except those of H_2O are omitted for clarity.

Compound: $[(phen)_2Zn(O_2CAr)]^+$ (Ar = *p*-C_6H_4OMe; phen = 1,10-phenanthroline)

Local Name: JK-1-43b

CCDC Number: 2342380

Table S10. Crystallographic details for $[(phen)_2Zn(O_2CAr)]^+$ (Ar = *p*-C_6H_4OMe; phen = 1,10-phenanthroline).

**Crystal data**	
Chemical formula	$2(C_{32}H_{23}N_4O_3Zn)\cdot C_8H_7O_3\cdot Cl\cdot 6.546(H_2O)$
M_r	1458.39
Crystal system, space group	Triclinic, $P\bar{1}$
Temperature (K)	150
a, *b*, *c* (Å)	14.9145 (17), 15.6856 (17), 15.8134 (17)
α, β, γ (°)	95.662 (4), 116.029 (4), 93.270 (4)
V (Å^3)	3287.1 (6)
Z	2
Radiation type	Mo *K*α
μ (mm^{-1})	0.85
Crystal size (mm)	0.31 × 0.19 × 0.07
**Data collection**	
Diffractometer	Bruker AXS D8 Quest
Absorption correction	Multi-scan *SADABS* 2016/2: Krause, L., Herbst-Irmer, R., Sheldrick G.M. & Stalke D., J. Appl. Cryst. 48 (2015) 3-10
T_{min}, T_{max}	0.661, 0.747
No. of measured, independent and observed [$I > 2s(I)$] reflections	72039, 24092, 13992
R_{int}	0.084
$(\sin θ/λ)_{max}$ (Å^{-1})	0.772
**Refinement**	
$R[F^2 > 2σ(F^2)]$, $wR(F^2)$, S	0.046, 0.120, 1.01
No. of reflections	24092
No. of parameters	986
No. of restraints	35
H-atom treatment	H atoms treated by a mixture of independent and constrained refinement
$Δρ_{max}$, $Δρ_{min}$ (e Å^{-3})	0.50, -0.71

Computer programs: Apex4 v2022.1-1 (Bruker, 2022), *SAINT* V8.40B (Bruker, 2020), *SHELXT* (Sheldrick, 2015b), *SHELXL2018*/3 (Sheldrick, 2015a, 2018), SHELXLE Rev1385 (Hübschle *et al.*, 2011).

Refinement details:

An area of water molecules and chloride anions is disordered over two alternative positions, induced by a different number of water molecules in the two moieties. Water H atom positions were refined and O-H and H...H distances were restrained to 0.84(2) and 1.36(2) Å, respectively. Some water H atom positions were further restrained based on hydrogen bonding considerations.

The positions of H atoms of water molecule O7B were refined while a damping factor was applied. In the final refinement cycles, the damping factor was removed and H7C and H7D were constrained to ride on their carrying atom O7B. Subject to these conditions, the occupancy ratio refined to 0.5460(17) to 0.4540(17).

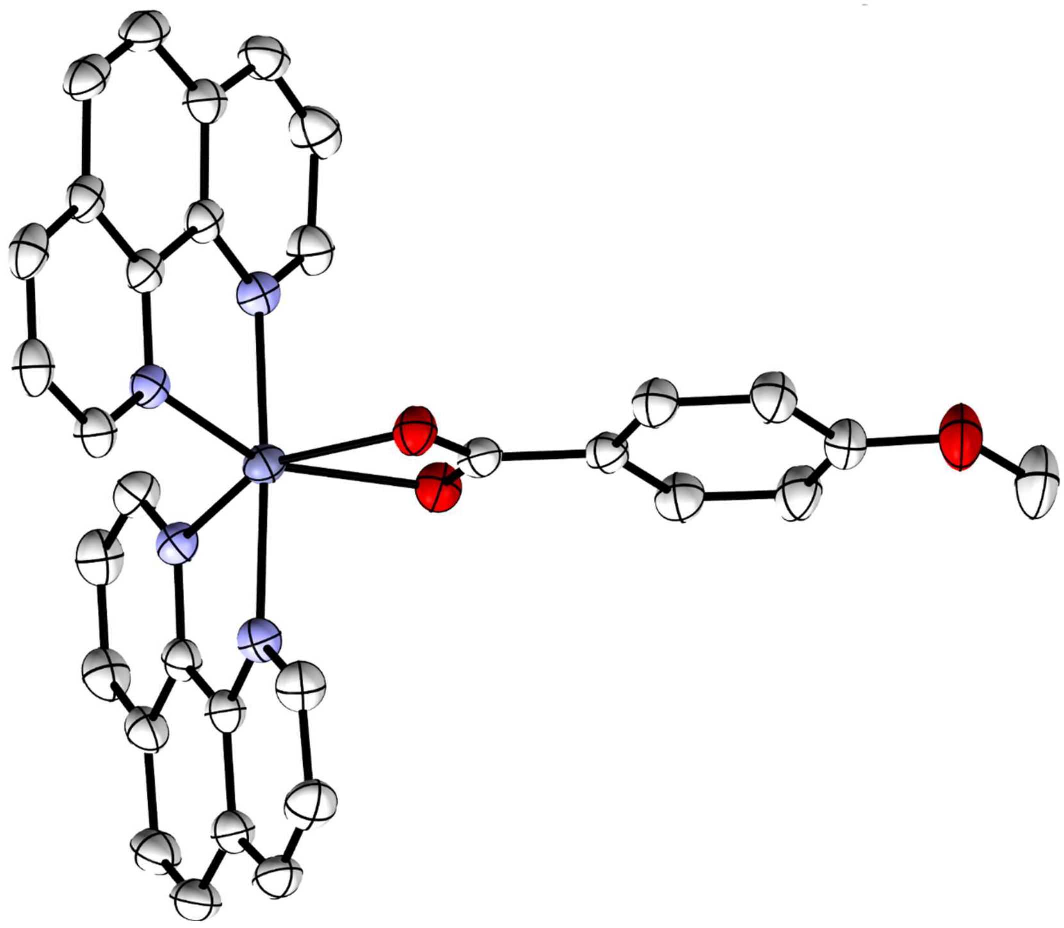

Figure S56. Molecular structure of the cation, $[(phen)_2Zn(O_2CAr)]^+$ (Ar = *p*-C_6H_4OMe; phen = 1,10-phenanthroline) displayed with 50% probability ellipsoids. All hydrogen atoms are omitted for clarity. Solvent molecules and anions are omitted for clarity.

Compound: *N*-(2-hydroxy-1,1-dimethylethyl)-4-(trifluoromethyl)benzamide

Local Name: JK-I-55

CCDC Number: 2342379

Table S11. Crystallographic details for *N*-(2-hydroxy-1,1-dimethylethyl)-4-(trifluoromethyl)benzamide.

**Crystal data**	
Chemical formula	$C_{12}H_{14}F_3NO_2$
M_r	261.24
Crystal system, space group	Monoclinic, $P2_1/c$
Temperature (K)	150
a, *b*, *c* (Å)	11.8997 (13), 12.3844 (15), 8.8409 (9)
β (°)	105.904 (5)
V (Å^3)	1253.0 (2)
Z	4
Radiation type	Mo *K*α
μ (mm^{-1})	0.12
Crystal size (mm)	0.45 × 0.32 × 0.10
**Data collection**	
Diffractometer	Bruker AXS D8 Quest
Absorption correction	Multi-scan *SADABS* 2016/2: Krause, L., Herbst-Irmer, R., Sheldrick G.M. & Stalke D. (2015). J. Appl. Cryst. 48, 3-10.
T_{min}, T_{max}	0.600, 0.747
No. of measured, independent and observed [*I* > 2s(*I*)] reflections	37007, 4794, 3568
R_{int}	0.078
(sin θ/λ)$_{max}$ (Å^{-1})	0.772
**Refinement**	
$R[F^2 > 2\sigma(F^2)]$, $wR(F^2)$, S	0.056, 0.158, 1.04
No. of reflections	4794
No. of parameters	170
H-atom treatment	H atoms treated by a mixture of independent and constrained refinement
Δρ$_{max}$, Δρ$_{min}$ (e Å^{-3})	0.49, -0.59

Computer programs: Apex4 v2022.10-1 (Bruker, 2022), *SAINT* V8.40B (Bruker, 2020), *SHELXT* (Sheldrick, 2015b), *SHELXL2019*/2 (Sheldrick, 2015a, 2019), SHELXLE Rev1573 (Hübschle *et al.*, 2011).

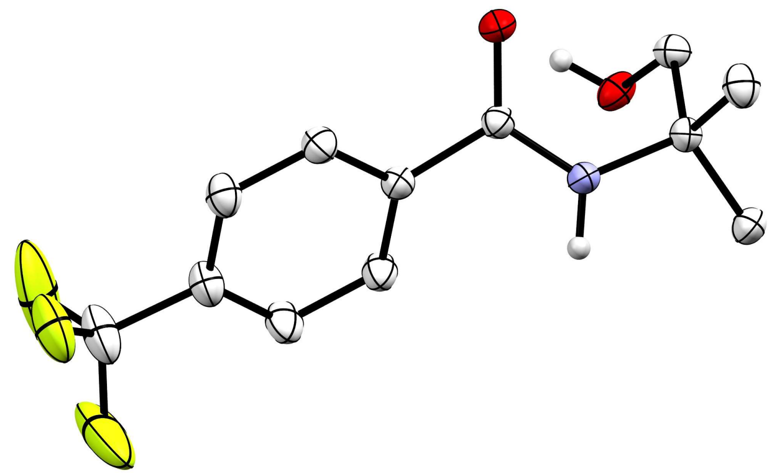

Figure S57. Molecular structure of *N*-(2-hydroxy-1,1-dimethylethyl)-4-(trifluoromethyl)benzamide displayed with 50% probability ellipsoids. All hydrogen atoms except those attached to heteroatoms are omitted for clarity.

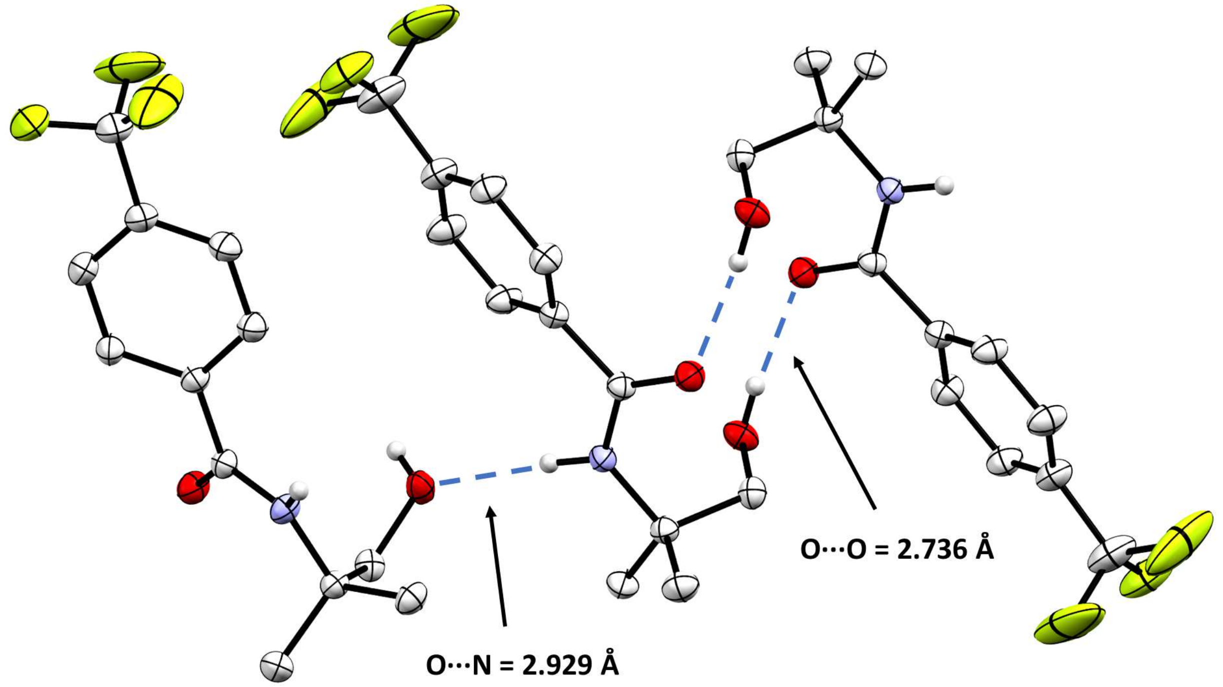

Figure S58. Extended structure of *N*-(2-hydroxy-1,1-dimethylethyl)-4-(trifluoromethyl)benzamide highlighting intermolecular hydrogen bonding interactions (50% probability ellipsoids). All hydrogen atoms except those attached to heteroatoms are omitted for clarity.

Compound: [{κ-N,O-$NH_2C(CH_3)_2CH_2OH$}$_2$Zn(O_2CAr)][O_2CAr] (Ar = *p*-C_6H_4OMe)

Local Name: LD1725

CCDC Number: 2394928

Table S12. [{κ-N,O-$NH_2C(CH_3)_2CH_2OH$}$_2$Zn(O_2CAr)][O_2CAr] (Ar = *p*-C_6H_4OMe).

**Crystal data**	
Chemical formula	$C_{24}H_{36}N_2O_8Zn$
M_r	545.92
Crystal system, space group	Triclinic, $P\bar{1}$
Temperature (K)	150
a, *b*, *c* (Å)	8.2414 (3), 12.5241 (5), 13.4050 (6)
α, β, γ (°)	106.214 (1), 92.498 (1), 104.589 (1)
V (Å^3)	1276.04 (9)
Z	2
Radiation type	Cu *K*α
μ (mm^{-1})	1.76
Crystal size (mm)	0.37 × 0.18 × 0.06
**Data collection**	
Diffractometer	Bruker AXS D8 Quest
Absorption correction	Multi-scan *SADABS* 2016/2: Krause, L., Herbst-Irmer, R., Sheldrick G.M. & Stalke D., J. Appl. Cryst. 48 (2015) 3-10
T_{min}, T_{max}	0.510, 0.754
No. of measured, independent and observed [$I > 2s(I)$] reflections	30387, 5461, 5312
R_{int}	0.039
(sin θ/λ)$_{max}$ (Å^{-1})	0.639
**Refinement**	
$R[F^2 > 2\sigma(F^2)]$, $wR(F^2)$, S	0.028, 0.071, 1.07
No. of reflections	5461
No. of parameters	340
H-atom treatment	H atoms treated by a mixture of independent and constrained refinement
$\Delta\rho_{max}$, $\Delta\rho_{min}$ (e Å^{-3})	0.29, -0.36

Computer programs: Apex5 v2023.9-2 (Bruker, 2023), *SAINT* V8.40B (Bruker, 2020), *SHELXT* (Sheldrick, 2015b), *SHELXL2019*/2 (Sheldrick, 2015a, 2019), SHELXLE Rev1613 (Hübschle *et al.*, 2011).

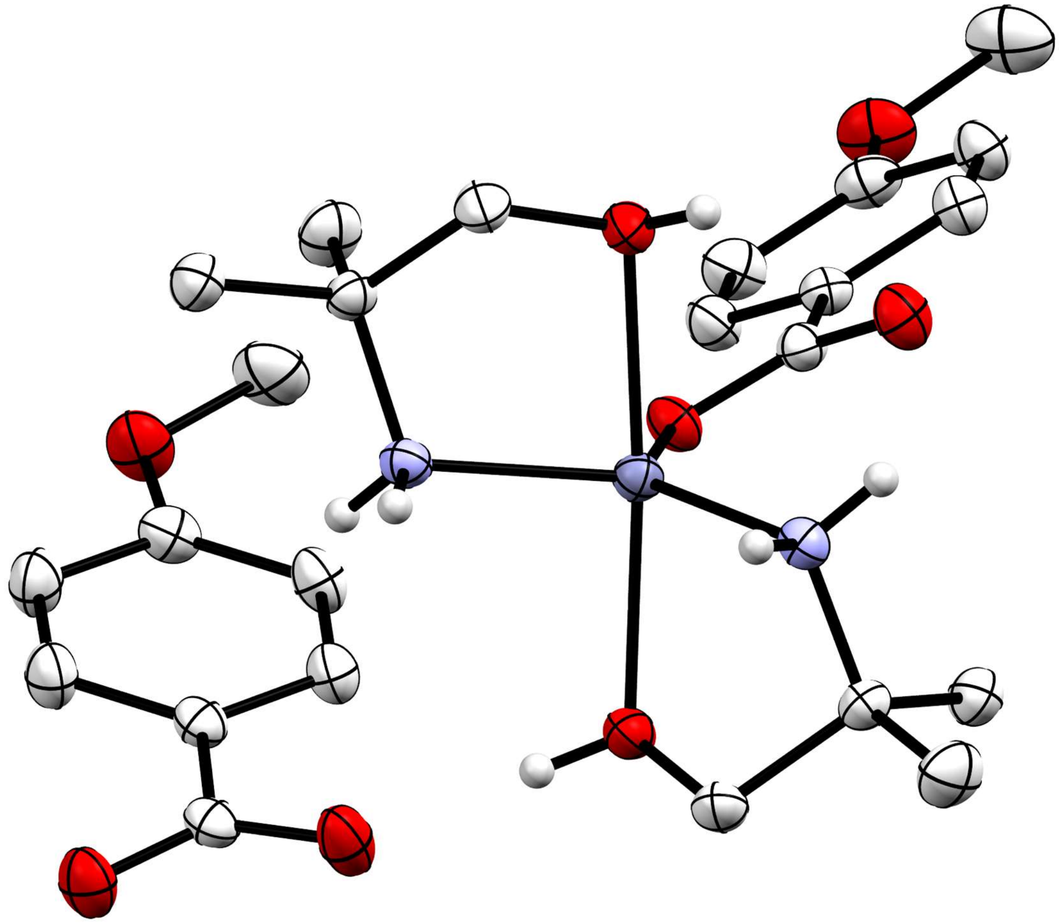

Figure S59. Molecular structure of [{κ-N,O-$NH_2C(CH_3)_2CH_2OH$}$_2$Zn(O_2CAr)][O_2CAr] (Ar = *p*-C_6H_4OMe) displayed with 50% probability ellipsoids. All hydrogen atoms except those attached to heteroatoms are omitted for clarity.

Table S13. Experimentally determined metrical parameters for $Zn_4O(O_2CAr)_6$ (Ar = *p*-$C_6H_4OCH_3$).

**2-OMe**		
**Zn1-Zn4 Cluster**	**Zinc-Carboxylate Distances (Zn-O)**	
	Zn1-O3	1.935(5) Å
	Zn1-O6	1.944(5) Å
	Zn1-O8	1.936(5) Å
	Zn2-O5	1.955(5) Å
	Zn2-O11	1.942(5) Å
	Zn2-O17	1.948(5) Å
	Zn3-O9	1.946(5) Å
	Zn3-O15	1.953(5) Å
	Zn3-O18	1.945(5) Å
	Zn4-O2	1.940(5) Å
	Zn4-O12	1.942(6) Å
	Zn4-O14	1.942(5) Å
	**Zinc-μ_4-oxo distances (Zn-O)**	
	Zn1-O1	1.953(4) Å
	Zn2-O1	1.935(5) Å
	Zn3-O1	1.947(5) Å
	Zn4-O1	1.954(5) Å
	**Geometry Index (τ_4 value)**	
	Zn1	0.966
	Zn2	0.965
	Zn3	0.974
	Zn4	0.968
**Zn5-Zn8 Cluster**	**Zinc-Carboxylate Distances (Zn-O)**	
	Zn5-O21	1.957(5) Å
	Zn5-O24	1.942(5) Å
	Zn5-O27	1.933(5) Å
	Zn6-O28	1.942(5) Å
	Zn6-O31	1.949(5) Å
	Zn6-O34	1.946(5) Å
	Zn7-O25	1.946(5) Å
	Zn7-O30	1.950(5) Å
	Zn7-O37	1.962(5) Å
	Zn8-O22	1.928(5) Å
	Zn8-O33	1.943(6) Å
	Zn8-O36	1.932(5) Å
	**Zinc-μ_4-oxo distances (Zn-O)**	
	Zn5-O20	1.940(5) Å
	Zn6-O20	1.953(5) Å
	Zn7-O20	1.938(5) Å
	Zn8-O20	1.940(4) Å
	**Geometry Index (τ_4 value)**	
	Zn5	0.960

	Zn6	0.955
	Zn7	0.967
	Zn8	0.976

Table S14. Experimentally determined metrical parameters for $Zn_4O(O_2CAr)_6(dmso)_2$ (Ar = *p*-$C_6H_4NMe_2$).

**2-NMe$_2$**		
	**Zinc-Carboxylate Distances (Zn-O)**	
	Zn1-O2_1	1.952(3) Å
	Zn1-O2_4	2.037(19) Å
	Zn1-O2_5	1.968(3) Å
	Zn2-O1_1	2.083(3) Å
	Zn2-O2_2	2.314(8) Å
	Zn2-O2_6	1.994(13) Å
	Zn3-O1_2	1.934(12) Å
	Zn3-O1_3	1.891(6) Å
	Zn3-O1_4	1.936(12) Å
	Zn4-O1_5	1.988(3) Å
	Zn4-O1_6	2.014(16) Å
	Zn4-O2_3	2.015(5) Å
	**Zinc-μ_4-oxo distances (Zn-O)**	
	Zn1-O1	1.912(2) Å
	Zn2-O1	1.988(3) Å
	Zn3-O1	1.891(6) Å
	Zn4-O1	1.925(3) Å
	**Geometry Index (τ_4 value)[a]**	
	Zn1	0.943
	Zn2	n/a
	Zn3	0.818
	Zn4	0.904

[a]τ_4 values were calculated for the major moieties when disorder was present.

Table S15. Experimentally determined metrical parameters for $Zn(O_2CAr)_2(dmf)_2$ (Ar = *p*-$C_6H_4NO_2$).

**2-NO$_2$**		
	**Zinc-Carboxylate Distances (Zn-O)**	
	Zn1-O1	1.9647(8) Å
	Zn1-O2	2.725 Å
	**Zinc-DMF distances (Zn-O)**	
	Zn1-O5	1.9681(7) Å
	**Geometry Index (τ_4 value)**	
	Zn1	0.827

Table S16. Experimentally determined metrical parameters for both (tmeda)Zn(O_2CAr)$_2$ compounds (Ar = *p*-$C_6H_4NO_2$ and *p*-C_6H_4OMe).

**3-NO$_2$**		
	**Zinc-Carboxylate Distances (Zn-O)**	
	Zn1-O1	2.3532(11) Å
	Zn1-O2	2.0404(10) Å
	**Zinc-tmeda distances (Zn-N)**	
	Zn1-N1	2.1243(11) Å
	**Geometry Index (τ_4 value)[a]**	
	Zn1	0.773
**3-OMe**		
	**Zinc-Carboxylate Distances (Zn-O)**	
	Zn1-O1	2.2005(8) Å
	Zn1-O2	2.1143(9) Å
	Zn1-O4	2.2573(9) Å
	Zn1-O5	2.0918(9) Å
	**Zinc-tmeda distances (Zn-N)**	
	Zn1-N1	2.1605(9) Å
	Zn1-N2	2.1379(9) Å
	**Geometry Index (τ_4 value)[a]**	
	Zn1	0.724

[a]τ_4 value calculated from atoms O2 and N1 for **3-NO$_2$** and atoms O2, O5, N1, and N2 for **3-OMe**.

Table S17. Experimentally determined metrical parameters for (tpy^R)Zn(O_2CAr)$_2$ (Ar = *p*-C_6H_4OMe; tpy^R = 4'-(4-methylphenyl)-2,2':6',2''-terpyridine).

**3-tpy^R**		
**Molecule containing Zn1**	**Zinc-Carboxylate Distances (Zn-O)**	
	Zn1-O1_1	1.9618(10) Å
	Zn1-O2_1	3.160 Å
	Zn1-O1_2	1.9710(9) Å
	Zn1-O2_2	2.792 Å
	**Zinc-tpy^R distances (Zn-N)**	
	Zn1-N1_4	2.2244(12) Å
	Zn1-N2_4	2.0816(10) Å
	Zn1-N3_4	2.1715(12) Å
	**Geometry Index (τ_5 value)**	
	Zn1	0.196
**Molecule containing Zn2[a]**	**Zinc-Carboxylate Distances (Zn-O)**	
	Zn2-O1_3	1.9831(10) Å
	Zn2-O2_3	2.864 Å
	**Zinc-tpy^R distances (Zn-N)**	
	Zn2-N1_5	2.1787(11) Å
	Zn2-N2_5	2.0872(15) Å
	**Geometry Index (τ_5 value)**	
	Zn2	0.310

[a]Molecule contains a C_2 rotation axis relating each half of tpy^R and each benzoate.

Table S18. Experimentally determined metrical parameters for $[(phen)_2Zn(O_2CAr)]^+$ (Ar = *p*-C_6H_4OMe; phen = 1,10-phenanthroline).

**3-phen’**		
**Molecule containing Zn1_2**	**Zinc-Carboxylate Distances (Zn-O)**	
	Zn1_2-O1_2	2.0227(14) Å
	Zn1_2-O2_2	2.457(2) Å
	**Zinc-phen distances (Zn-N)**	
	Zn1_2-N1_2	2.0943(16) Å
	Zn1_2-N2_2	2.1809(15) Å
	Zn1_2-N3_2	2.1531(17) Å
	Zn1_2-N4_2	2.1500(15) Å
**Molecule containing Zn1_3**	**Zinc-Carboxylate Distances (Zn-O)**	
	Zn1_3-O1_3	2.2202(13) Å
	Zn1_3-O2_3	2.1672(12) Å
	**Zinc-phen distances (Zn-N)**	
	Zn1_3-N1_3	2.1210(15) Å
	Zn1_3-N2_3	2.1217(14) Å
	Zn1_3-N3_3	2.1393(16) Å
	Zn1_3-N4_3	2.1308(14) Å

Table S19. Experimentally determined metrical parameters for $(phen)Zn(O_2CAr)_2(H_2O)$ (Ar = *p*-C_6H_4OMe; phen = 1,10-phenanthroline).

**3-phen’’**		
	**Zinc-Carboxylate Distances (Zn-O)**	
	Zn1_1-O1_2	2.0067(9) Å
	Zn1_1-O2_2	2.767 Å
	Zn1_1-O1_3	2.0909(9) Å
	Zn1_1-O2_3[a]	3.326 Å
	**Zinc-phen distances (Zn-N)**	
	Zn1_1-N1_4	2.1692(10) Å
	Zn1_1-N2_4	2.1083(10) Å
	**Zinc-H_2O distances (Zn-O)**	
	Zn1_1-O1_1	2.0297(9) Å
	**Geometry Index (τ_5 value)[b]**	
	Zn1_1	0.476

[a]Oxygen is hydrogen bonding with the zinc-bound water (O····H-O = 2.577 Å for O-O distance).
[b]Calculated assuming O2_2 is not bonded.

Table S20. Experimentally determined metrical parameters for [{κ-N,O-$NH_2C(CH_3)_2CH_2OH$}$_2$Zn(O_2CAr)][O_2CAr] (Ar = *p*-C_6H_4OMe).

Zn-NH_2R	2.0334(11) Å
Zn-NH_2R	2.0543(11) Å
Zn-O(H)R	2.1774(10) Å
Zn-O(H)R	2.2221(10) Å
Zn-O (benzoate)	1.9570(9) Å
τ_5 value	0.680

References

(1) Zhang, H.; Xu, C.; Zhan, X.; Yu, Y.; Zhang, K.; Luo, Q.; Gao, S.; Yang, J.; Xie, Y. Mechanistic insights into CO_2 conversion chemistry of copper bis-(terpyridine) molecular electrocatalyst using accessible operando spectrochemistry. *Nat. Commun.* **2022**, *13*, 6029.

(2) *Bruker Advanced X-ray Solution, Apex3, SAINT, SADABS, Bruker AXS Inc.: Madison (WI), USA, 2018.*

(3) *SHELXTL suite of programs, Version 6.14, 2000-2003, Bruker Advanced X-ray Solutions, Bruker AXS Inc., Madison, Wisconsin: USA*.

(4) Sheldrick, G. A short history of SHELX. *Acta Cryst.* **2008**, *A64*, 112-122.

(5) Sheldrick, G. Crystal structure refinement with SHELXL. *Acta Cryst.* **2015**, *C71*, 3-8.

(6) Hubschle, C. B.; Sheldrick, G. M.; Dittrich, B. ShelXle: a Qt graphical user interface for SHELXL. *J. Appl. Crystallogr.* **2011**, *44*, 1281-1284.

(7) Göbel, D.; Rusch, P.; Duvinage, D.; Stauch, T.; Bigall, N.-C.; Nachtsheim, B. J. Substitution Effect on 2-(Oxazolinyl)-phenols and 1,2,5-Chalcogenadiazole-Annulated Derivatives: Emission-Color-Tunable, Minimalistic Excited-State Intramolecular Proton Transfer (ESIPT)-Based Luminophores. *J. Org. Chem.* **2021**, *86*, 14333-14355.

(8) Takaya, J.; Sangu, K.; Iwasawa, N. Molybdenum(0)-Promoted Carbonylative Cyclization of o-Haloaryl- and β-Haloalkenylimine Derivatives by Oxidative Addition of a Carbon(sp2)-Halogen Bond: Preparation of Two Types of γ-Lactams. *Angew. Chem. Int. Ed.* **2009**, *48*, 7090-7093.

(9) Necefoglu, H.; Clegg, W.; Scott, A. J. Diaquabis(4-nitrobenzoato)zinc(II): a redetermination. *Acta Cryst.* **2001**, *E57*, m472-m474.

(10) Deng, Z.-P.; Gao, S.; Huo, L.-H.; Ng, S. W. Diaquabis(4-formylbenzoato-κ*O*)zinc(II). *Acta Cryst.* **2008**, *E64*, m447.

(11) Deng, Z.-P.; Gao, S.; Huo, L.-H.; Zhao, H. Diaquabis(4-formylbenzoato-κ*O*)zinc(II) monohydrate. *Acta Cryst.* **2006**, *E62*, m3524-m3526.

(12) Potocnak, I.; Dunaj-Jurco, M.; Cernak, J. Structure of diaquabis(4-chlorobenzoato)zinc(II). *Acta Cryst.* **1993**, *C49*, 1496-1498.

(13) Hokelek, T.; Caylak, N.; Necefoglu, H. Diaquabis(4-bromobenzoato-κ^2*O*,*O'*)zinc(II). *Acta Cryst.* **2008**, *E64*, m458-m459.

(14) Bury, W.; Justyniak, I.; Prochowicz, D.; Rola-Noworyta, A.; Lewiński, J. Oxozinc Carboxylate Complexes: A New Synthetic Approach and the Carboxylate Ligand Effect on the Noncovalent-Interactions-Driven Self-Assembly. *Inorg. Chem.* **2012**, *51*, 7410-7414.

(15) Clark, G. L.; Kao, H. A Versatile Technique for X-Ray Single Crystal Structural Analysis Applied to Benzaldehyde 2,4-Dinitrophenylhydrazone and Zinc Salts of Salicylic and Benzoic Acids. *J. Am. Chem. Soc.* **1948**, *70*, 2151-2154.

(16) Clegg, W.; Harbron, D. R.; Homan, C. D.; Hunt, P. A.; Little, I. R.; Straughan, B. P. Crystal structures of three basic zinc carboxylates together with infrared and FAB mass spectrometry studies in solution. *Inorg. Chim. Acta.* **1991**, *186*, 51-60.

(17) Lewiński, J.; Bury, W.; Dutkiewicz, M.; Maurin, M.; Justyniak, I.; Lipkowski, J. Alkylzinc Carboxylates as Efficient Precursors for Zinc Oxocarboxylates and Sulfidocarboxylates. *Angew. Chem. Int. Ed.* **2008**, *47*, 573-576.

(18) Ma, D.-Y.; Deng, G.-H.; Song, W.-D. Diaquabis(4-methylbenzoato-κ*O*)zinc(II). *Acta Cryst.* **2008**, *E64*, m32.

(19) Karmakar, A.; Baruah, J. B. Synthesis and characterization of zinc benzoate complexes through combined solid and solution phase reactions. *Polyhedron* **2008**, *27*, 3409-3416.

(20) Cimen, E.; Gumus, I.; Arslan, H. The role of intermolecular interactions in the assembly of Zinc(II) and Lead(II) complexes containing carboxylate ligand and their conversion to metal oxides. *J. Mol. Struct.* **2018**, *1166*, 397-406.

(21) Zhang, L.; Qin, Y.-Y.; Li, Z.-J.; Lin, Q.-P.; Cheng, J.-K.; Zhang, J.; Yao, Y.-G. Topology Analysis and Nonlinear-Optical-Active Properties of Luminescent Metal−Organic Framework Materials Based on Zinc/Lead Isophthalates. *Inorg. Chem.* **2008**, *47*, 8286-8293.
